# Reducing the Inner Filter Effect in Microplates by Increasing Absorbance? Linear Fluorescence in Highly Concentrated Fluorophore Solutions in the Presence of an Added Absorber

**DOI:** 10.1021/acs.analchem.3c01295

**Published:** 2023-08-22

**Authors:** Tomislav Friganović, Tin Weitner

**Affiliations:** Faculty of Pharmacy and Biochemistry, University of Zagreb, Ante Kovačića 1, 10000 Zagreb, Croatia

## Abstract

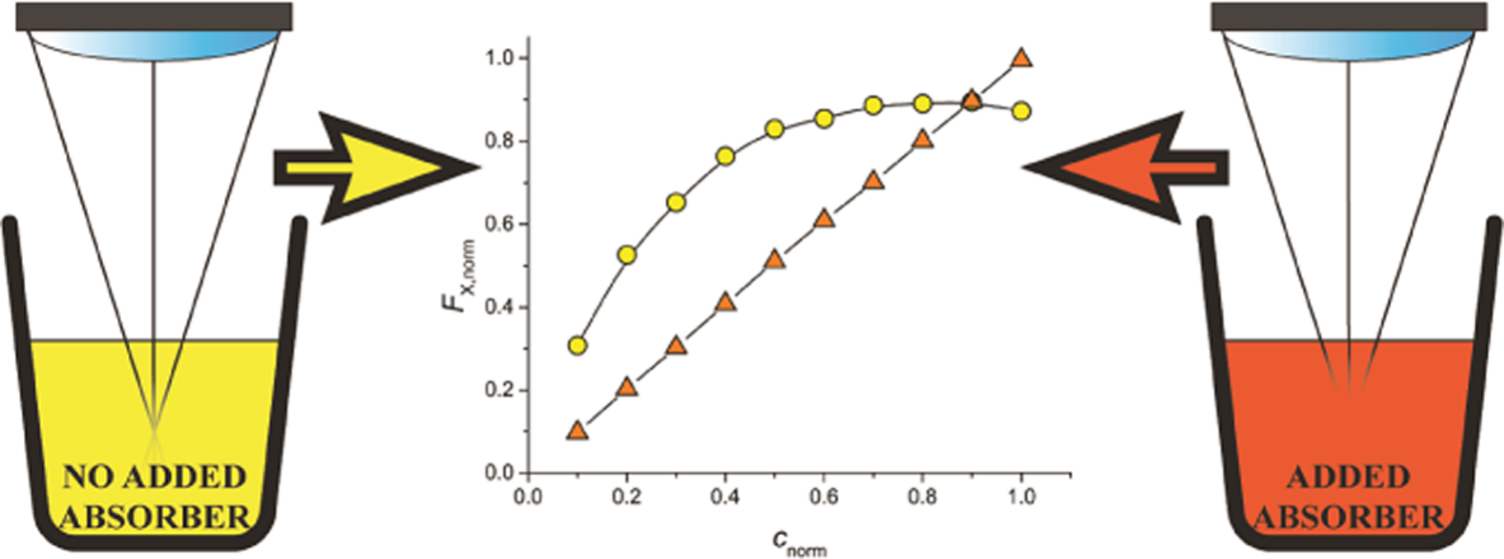

The fluorescence attenuation caused by the absorption of the excitation and/or emission light is called the Inner Filter Effect (IFE) and can lead to a nonlinear fluorescence concentration response. In this article, we propose the AddAbs (Added Absorber) method, which counterintuitively corrects IFE by increasing the absorbance of the sample. In this method, an equal amount of a highly absorbing chromophore is added to each sample to compensate for the nonuniform quenching caused by different fluorophore concentrations. The AddAbs method was able to provide a linear fluorescence response (*R*^2^ > 0.999) for very concentrated fluorophore solutions with extreme IFE over more than 97% of the concentration range with less than 1% deviation in calibration slope. The true limit for the AddAbs method with respect to fluorophore concentration was apparently not reached and could be higher than measured (*A*_ex,1cm_ > 33.94). The IFE-corrected data are obtained by a single fluorescence measurement per sample without additional mathematical procedures. The method also does not require absorbance measurements, so it can be performed in non-transparent microplates with similar results. In addition, preliminary measurements indicate that the method is also suitable for measurements in standard cuvettes using a fluorimeter with a 90° angle setup.

## Introduction

### Fluorescence Spectroscopy and the Inner Filter Effect

Fluorescence spectroscopy has proven to be a very powerful tool for the study of various chemical systems, with numerous applications in medical diagnostics and imaging, as well as in biological, chemical, material, engineering, and other sciences.^1−3^ One of the greatest advantages of fluorescence spectroscopy is its high sensitivity, meaning that low concentrations of analytes can usually be accurately determined. Recent advances in this field have contributed significantly to speed and simplicity while reducing the cost of measurements.^4^ High-throughput measurements can be performed using microplate readers and liquid handling devices with low sample volume.

A major problem with various fluorescence measurements is the nonlinear dependence of the relative fluorescence intensity signal on analyte concentration.^2^ The fluorescence quenching effect caused by the absorption of excitation and/or emission light is called the Inner Filter Effect (IFE). This effect can be divided into two separate phenomena: the primary and secondary inner filter effect (pIFE and sIFE, respectively).^3^ The pIFE is caused by absorption of radiation at the excitation wavelength (λ_ex_), while the sIFE is caused by absorption at the emission wavelength (λ_em_). Both effects lead to decreased measured fluorescence, and both can occur individually or together contribute to the overall loss of signal. The extent of IFE depends on both the spectral properties of the sample and the geometrical parameters of illumination. Solutions with high optical densities at the excitation and/or emission wavelength(s) should exhibit significant IFE.^4,5^

### Common IFE Correction Strategies

Many different laboratory techniques and mathematical methods are used to correct the nonlinear fluorescence concentration response caused by IFE.^6^ One very simple and commonly used method to reduce IFE relies on sample dilution.^7^ Sufficiently diluted samples have a much lower optical density, so IFE is greatly reduced (preferably negligible). The main disadvantage of this method is the fact that the dilution procedure is never perfect. It introduces additional errors in the concentration(s) of the investigated compound(s). In addition, severe dilution may disturb the system under study in unexpected and undesirable ways (e.g., it may disrupt colloidal stability or shift chemical equilibrium). The advantage of the dilution method is the fact that no complicated mathematical procedure needs to be applied, only the total dilution factor needs to be considered to estimate the IFE-corrected fluorescence of the undiluted sample.

Another common strategy for IFE correction takes the sample absorbance into consideration, and there are plenty of absorbance-based IFE correction methods in the literature.^5−7^ Probably the most commonly used method by other researchers is the one proposed by Lakowicz in his popular textbook on fluorescence spectroscopy.^4^ This correction is shown in eq 1, where *F*_A_ is the IFE-corrected fluorescence; *F*_0_ is the IFE-uncorrected fluorescence; and *A*_ex_ and *A*_em_ are the absorbance values at the excitation and emission wavelengths, respectively.
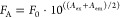
1A major weakness of this method is that reasonably good corrections can be obtained only in a relatively narrow absorbance interval. For example, Panigrahi and Mishra note that the Lakowicz model loses its efficiency at absorbance values of 0.7, a claim we confirm both in this work and in our earlier publication.^5,8^ Another obvious drawback is that both fluorescence and absorbance must be measured for each sample. This can be especially important for measurements in microplates, where the measurement of absorbance and fluorescence can be much more expensive if the use of UV–vis–transparent microplates is required.

Yet another strategy for IFE correction takes advantage of the fact that by changing the geometric parameters and thus the effective path lengths of the sample illumination, the difference in measured fluorescence can be used to correct IFE. For measurements in the cuvette, a cell shift method was developed, while for microplate readers with adjustable vertical axis focus, we developed the ZINFE method according to eq 2, where *F*_Z_ is the ZINFE-corrected fluorescence intensity, *F*_0(*z*1)_ and *F*_0(*z*2)_ are measured fluorescence values at different *z*-positions, *z*_1_ and *z*_2_, whereas *k* is a geometric parameter specific for a particular sample volume, microplate, and microplate reader type.^8,9^
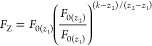
2This equation can be simplified by including a single-exponential term *N* corresponding to a particular combination of *k*, *z*_1_, and *z*_2_, according to eq 3, where *F*_N_ is the NINFE-corrected fluorescence intensity based on fluorescence measurements at different *z*-positions (*F*_0(*z*1)_ and *F*_0(*z*2)_), and the exponential term *N* is obtained by brute-force optimization.
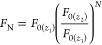
3We have shown that both the ZINFE and NINFE methods are suitable for simultaneous correction of pIFE and sIFE to within 1.3% for a maximum sample absorbance of at least *A*_ex_ ≈ 2 and *A*_em_ ≈ 0.5, with possible applicability at higher absorbance values (normalized to 1 cm optical path length, the corresponding values are *A*_ex,1cm_ ≈ 4 and *A*_em,1cm_ ≈ 1).^8^

### Increasing IFE on Purpose—The Added Absorber Method

In our previous work on ZINFE/NINFE correction methods, we observed less curvature of the fluorescence signal in the presence of an additional constant background absorbance compared to samples with either the fluorophore alone or with a proportional amount of added absorber (i.e., with a fixed ratio of fluorophore and absorber concentration). In the experiments with constant background absorbance, the concentration of the added absorber corresponded to *A*_ex_ ≈ 1 and therefore the observed lower curvature was attributed to a lower variability of the total absorbance at the excitation wavelength for this concentration series compared to others. Nevertheless, this observation prompted us to further investigate the effects of impurities on the fluorescence signal. For additional consideration of IFE in the presence of an added absorber, see Section 4.2 in the Supporting Information.

In a typical calibration experiment, the molar absorbance coefficients can be considered constant with invariant solvent composition, as can the optical path with invariant spatial dimensions of the cuvette or equal total volume pipetted per microplate well. In this case, the total absorbance at the excitation or emission wavelength is equal to the absorbance of the fluorophore at that wavelength if no additional absorbers are present in the solution. When a solution contains only a pure fluorophore dissolved in a nonabsorbing solvent, the absorbances of that solution at the emission and excitation wavelengths are proportional to the concentration of the fluorophore, the molar absorbance coefficients, and the optical path length. The nonuniform illumination of the sample caused by IFE again leads to a deviation from the ideal linear fluorescence concentration response.

If a fixed amount of a (nonfluorescent) UV–vis–absorbing compound is present in the solution, the increase in total absorbance of the solution will correspond to the absorbance component of this added absorber, assuming that these compounds do not react. Since the IFE is a function of the total absorbance on both excitation (pIFE) and emission (sIFE), the solution containing only the strong added nonfluorescent absorber and the solution containing both the absorber and the fluorescent compound will both have a similar IFE. Consequently, a smaller deviation from the ideal linear fluorescence concentration response can be expected. This property allows us to use the “fight fire with fire” strategy to reduce the nonlinearity of the fluorescence signal caused by IFE by intentionally increasing IFE. At first glance, this principle may seem counterintuitive, considering that the standard strategy for reducing nonlinearity caused by IFE is primarily to reduce IFE rather than the other way around.

A similar concept of combining fluorophores and chromophores for analytical purposes is known as IFE-based sensing and numerous examples can be found in the literature. This principle of tuning the absorber concentration to develop a fluorescence-based assay for a selective analyte usually involves a turn-off method in which the absorbance of the analyte increases at either the excitation or emission wavelength of the fluorophore, resulting in a decrease in the fluorescence emission intensity of the fluorophore. Other, less common, variations of the IFE-based measurement include the turn-on method, in which the absorbance of the absorber decreases with the addition of the analyte, and the ratiometric method, in which the assay is determined by the ratio of the two emission intensity curves of the titration experiment.^5^ However, the main difference with the AddAbs method is that in the IFE-based sensing methods, the concentration of the fluorophore is kept constant, while the change in absorbance of the absorber (and thus IFE) is related to the analytical signal.

### Objectives and Scope

**Figure 1:**
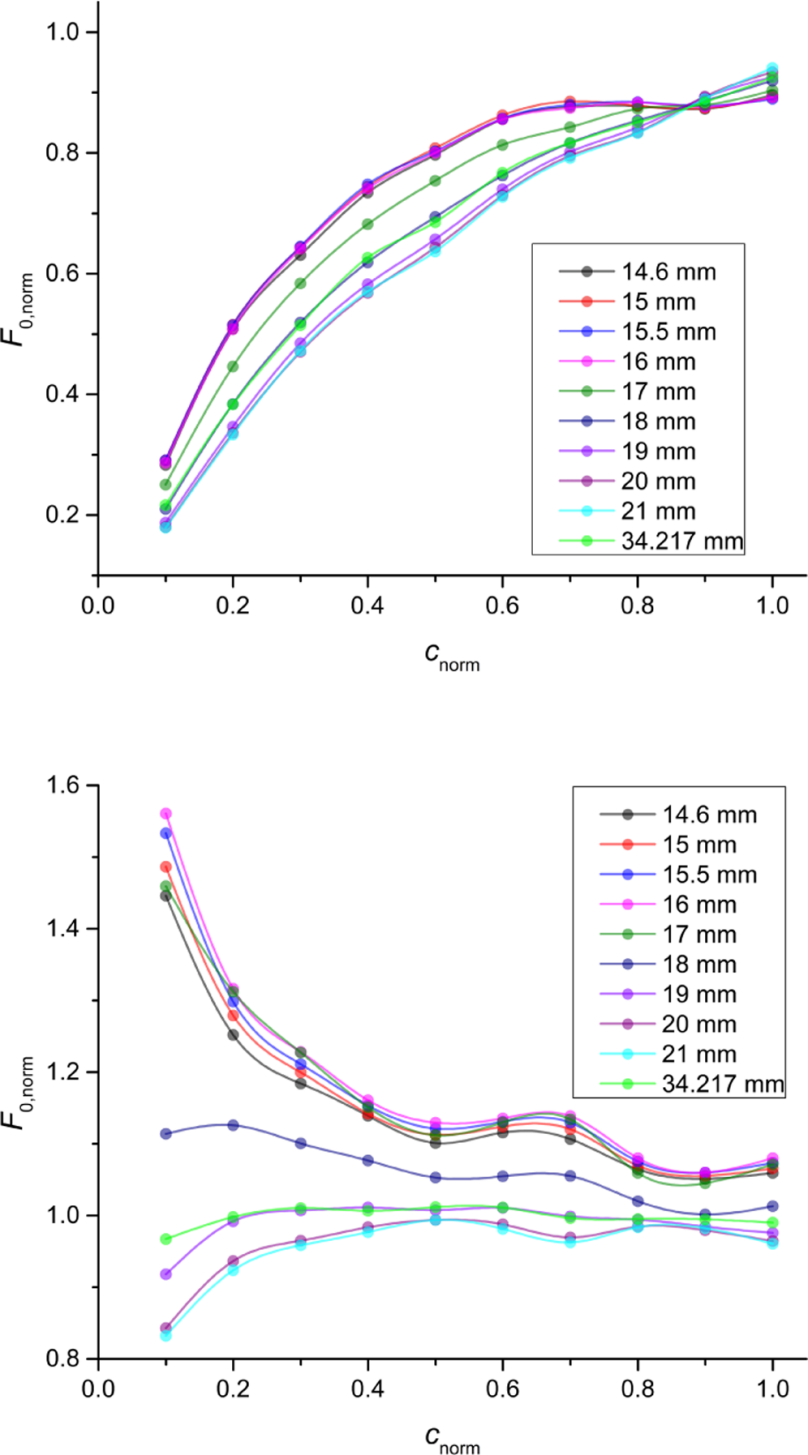
Normalized uncorrected fluorescence intensity values, *F*_0,norm_, recorded at 10 different *z*-positions and plotted as a function of scaled QS concentration, *c*_norm_. **Top:** L_1_ titration in UV–vis–transparent (T) microplate. **Bottom:** H_1_ titration in non-transparent (NT) microplate.

**Figure 2:**
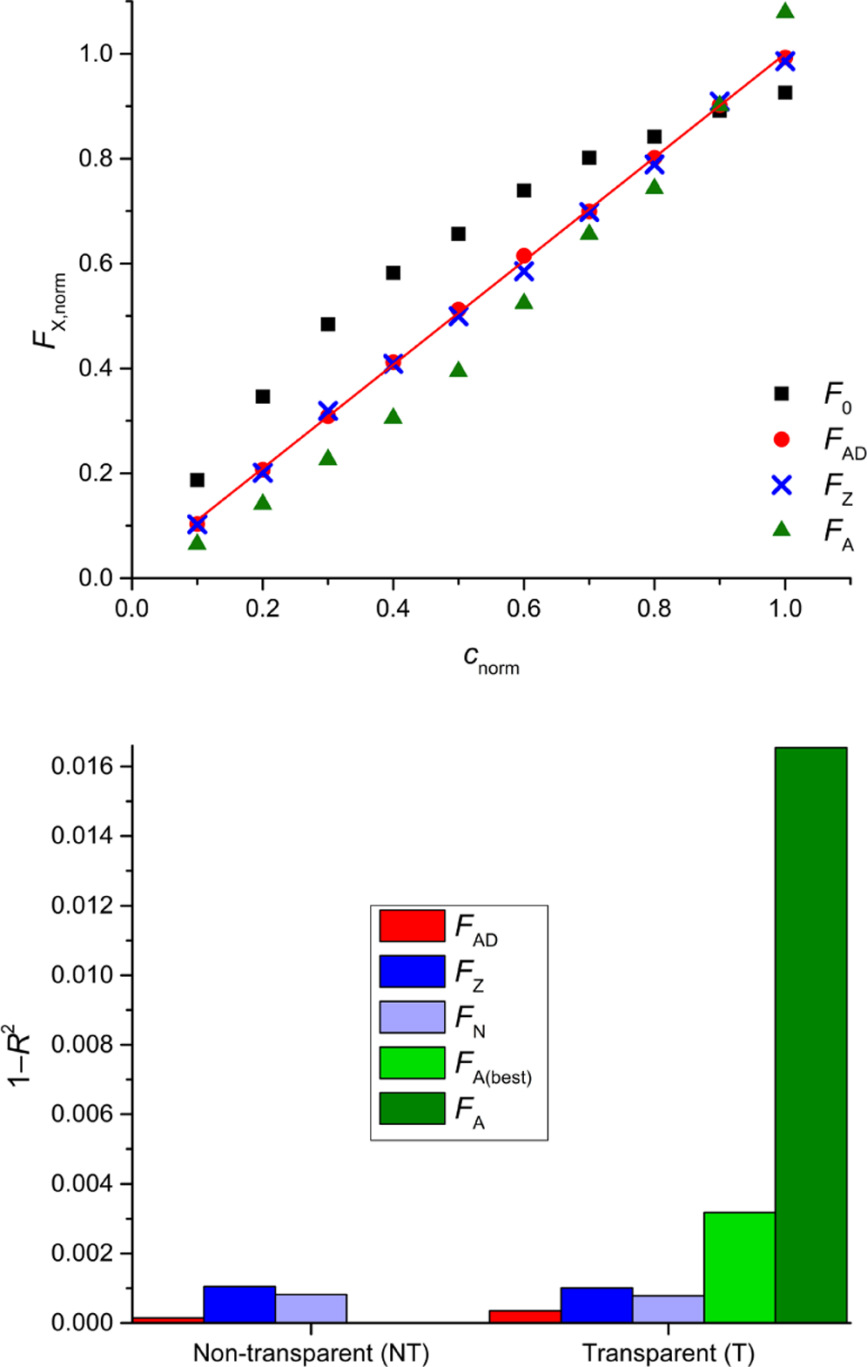
Overview of the different IFE corrections for low fluorophore concentrations, corresponding to the results in Table 1 (T microplates) and Table S4 (NT microplates). The values of *F*_AD_, *F*_Z_, *F*_N_, *F*_A(best)_, and *F*_A_ correspond to AddAbs correction, ZINFE correction, NINFE correction, best Lakowicz correction from the whole set of *z*-positions, and Lakowicz correction obtained at the same *z*-position as *F*_AD_, respectively. **Top:** Normalized IFE-corrected fluorescence data for L_8_ titration in T microplate. Normalized uncorrected fluorescence *F*_0_ (L_1_ titration) is shown for comparison, and the values of *F*_N_ are omitted for clarity due to the high similarity with the *F*_Z_ values. **Bottom:** 1 – *R*^2^ values obtained for the different types of IFE corrections in T and NT microplates. Lower 1 – *R*^2^ values indicate better linearity.

The aim of this work is to validate the proposed principle of Added Absorber IFE correction (AddAbs) by using different amounts of an added absorber to samples with known amounts of pure fluorophore in microplates and comparing the obtained fluorescence with data collected for the fluorophore alone. The added absorber should absorb significantly at either emission and/or excitation wavelengths, causing the additional IFE, while not quenching the fluorescence of the analyte by mechanisms other than IFE. If such an absorber is present at a constant concentration for all samples, it is expected that the intrinsic IFE caused by the fluorophore will be enhanced so that the IFE-uncorrected fluorescence concentration response curves shown in Figure 1 will have less curvature, like the data shown in Figure 2 (top).

We present evidence for the efficiency of the AddAbs method using fluorescence measurements performed for 2 different fluorophore concentration ranges (low and high) in two different types of microplates (UV–vis–transparent and non-transparent). We also investigated the possible influence of sample geometry by performing measurements at different vertical axis positions of the optical element of the microplate reader (*z*-positions). To perform benchmarking, we compared the proposed AddAbs method with values obtained using the Lakowicz method and the ZINFE/NINFE method described previously.^8^ A tentative combination of the AddAbs method with the previously described ZINFE/NINFE method was also demonstrated.

## Experimental Section

### Reagents and Instrumentation

Similar to our earlier work,^8^ the IFE correction was first evaluated using a concentration series of a known fluorophore quinine sulfate (QS), while potassium dichromate (PD) was chosen as an added absorber since it is known to absorb light at both the excitation and emission wavelengths of QS (thus allowing correction of both pIFE and sIFE) without itself exhibiting fluorescence (Figure S1). A total of 15 different titrations were performed for two different ranges of QS concentrations: (i) low QS concentration range (titrations L_1_–L_12_), and (ii) high QS concentration range (titrations H_1_–H_3_). Full details of sample preparation and titration procedures are given in Sections 2 and 3 in the Supporting Information. Since the concentration is directly proportional to the absorbance, we find it more convenient to express the amounts of QS and PD in terms of their estimated absorbance at *l* = 1 cm.

All titrations were performed in triplicate, and the averaged baseline-corrected values of each triplicate were used for data analysis. Titration experiments were performed in parallel in two different types of 96-well microtiter plates: (i) the UV–vis–transparent (T) microplates (black, 96-well, μ-clear, flat bottom, chimney well, cat. no. 655097, Greiner, USA) and (ii) the non-transparent (NT) microplates (black, 96-well, flat bottom, cat. no. 30122298, Tecan, Austria). The total volume of liquid per microplate well was set to 200 μL.

Samples for each concentration series were prepared using the Tecan Spark M10 multimode microplate reader titrator module (Tecan, Austria). Fluorescence intensity measurements were performed in fluorescence top-reading mode with a single excitation and emission wavelength: λ_ex_ = 345 nm (ε(QS) = 5700 M^–1^ cm^–1^, ε(PD) = 2939 M^–1^ cm^–1^), λ_em_ = 390 nm (ε(QS) = 348 M^–1^ cm^–1^, ε(PD) = 1049 M^–1^ cm^–1^).^8^ Fluorescence was measured at 10 different vertical positions of the microplate reader optical element (*z*-positions, Table S3) for each sample in both T and NT microplates. The values of the *z*-positions were in the range of 14.6–34.217 mm, which corresponds to the maximum range of adjustable *z*-positions that depends on the dimensions of the microplates. Absorbance spectra were recorded in the range of 200–1000 nm in steps of 1 nm for T microplates only. The temperature variations (i.e., the difference between maximum and minimum temperature) during the measurement of fluorescence intensity for multiple *z*-positions did not exceed 0.5 °C.

### Data Presentation and Evaluation

Uncorrected fluorescence measurements are given as *F*_0_, whereas we use the following notation throughout the text for the various IFE correction methods: *F*_AD_ (AddAbs method proposed in this work), *F*_A_ (absorbance-based Lakowicz method, eq 1), *F*_Z_ and *F*_N_ (ZINFE and NINFE methods described by eqs 2 and 3, respectively).^8^ ZINFE/NINFE corrections were made using a dedicated online service available at https://ninfe.science.^10^ All original and averaged triplicate data were archived for analysis and reference.^11^

The absolute values of the measured fluorescence intensities in the experiments differ considerably, which can lead to problems in the graphical presentation and interpretation of the data. Therefore, normalization of data was performed for both measured and IFE-corrected data. As a primary measure of linearity, we decided to use *R*^2^ (coefficient of determination) as the main criterion, i.e., data sets with *R*^2^ closest to 1 were considered the best IFE-corrected data sets and further evaluated for values of *b* % (percent error of the slope of the normalized data) and LOD % (percent error of the Limit Of Detection, LOD) for each titration and for each correction procedure.^8^ Full details on data normalization and evaluation are given in Section 4.5 in the Supporting Information. The validity of the proposed AddAbs IFE correction method was further verified by evaluating residuals (differences between the measured and predicted values) and by performing Leave-One-Out Cross-Validation (LOOCV, see details in Section 4.6 in the Supporting Information).

## Results and Discussion

### Uncorrected Fluorescence

Fluorescence data for the pure QS fluorophore without addition of PD (i.e., uncorrected fluorescence, *F*_0_) are shown in Figures 1, S2, and S3. Upon visual inspection, the normalized data deviate significantly from the ideal case in which *y* = *x* is expected. This deviation is largely due to the strong IFE and to a lesser extent a consequence of small experimental errors. Significant differences between the fluorescence curves are observed for the different *z*-positions, as noted and described in more detail in our earlier work.^8^ The type of microplate (T or NT) also affects the fluorescence intensity profiles, but to a much lesser extent.

Systematic trends can be identified. In L_1_ titration, each fluorescence set shows a clear downward curvature. This is a consequence of the nonuniform IFE quenching effect caused by the fluorophore (QS) itself. The quenching effect is much more pronounced in the H_1_ series. This is not surprising since the H_1_ series contains about 17 times higher QS concentration than the L_1_ series (*A*_345nm,max,1cm_ = 2.02 for L_1_ and 33.94 for H_1_). Due to the extreme IFE in H_1_, an immediate downward trend can be seen for the first and second points of titration, depending on the *z*-position (Figure 1, bottom). This means that the IFE is so strong that the more concentrated second titration point produces a weaker signal than the less concentrated first. With such an extreme IFE for the H_1_ titration, the change in fluorescence with the change in concentration can hardly be interpolated with a meaningful mathematical function.

For the L_1_ series, it seems reasonable to estimate the concentration of the fluorophore by nonlinear interpolation. For illustrative examples of nonlinear fitting, see Section 6 in the Supporting Information. Briefly, using a single-exponential fit with the formula *F*_exp,norm_ = *a* + *b* · ln(*c*_norm_) for the L_1_ titration (T microplates, *z* = 18 mm, Figure S37) yielded the values *R*^2^ = 0.9966, *s*_*y*_ = 0.0145 (defined by eq S4) and the approximate minimal normalized concentration of *c*_norm,min_ = 0.0559 (defined by eq S14). We propose these values of *c*_norm,min_ and *s*_*y*_ as surrogates for the lower bounds of the corresponding values of LOD % = 5.59% and *b* % = 1.45% to compare the values obtained by linear fitting of the IFE-corrected data. No nonlinear fitting was attempted for the H_1_ series because the curves obtained are very irregular and contain several local minima and maxima as well as plateau regions. It is difficult to imagine that a typical IFE correction procedure (apart from extreme dilution) could be successfully applied to such saturated samples. An overview of the calibration parameters for uncorrected data and all IFE corrections discussed in the following sections can be found in Table 1. For clarity, only the results for T microplates are given, while the corresponding results for NT microplates can be found in Table S4.

**Table 1:** Overview of the Least-Squares Linear Fit Results for Normalized Background-Corrected Fluorescence and Absorbance Data Obtained in UV–Vis–Transparent (T) Microplates

range^a^	series^b^	correction type^c^	*R*^2^	*b* %^d^	LOD %^e^	*z*_1_, mm	*z*_2_, mm	*A*_max,1cm_^f^ (λ_ex_, λ_em_)	*c*_max_^g^, mM
low	L_8_	*F*_AD_	0.99964	1.199	2.001	19		21.67, 7.14	0.353
	L_1_	*F*_0_	0.94631	21.266	25.241	19		2.01, 0.12	
		*F*_exp_^h^	0.99657	1.453	5.559	18			
		*F*_Z_	0.99899	0.969	3.374	18	16		
		*F*_N_	0.99899	1.035	3.373	18	16		
		*F*_N(best)_	0.99922	0.949	2.965	18	15.5		
		*F*_A_	0.98346	–10.352	13.742	19			
		*F*_A(best)_	0.99683	–0.394	5.979	15.5			
high	H_3_	*F*_AD_	0.99952	0.365	2.329	18		111.92, 29.91	5.954
	H_1_	*F*_0_	0.94031	123.963	–26.699	18		33.94, 2.07	
		*F*_Z_	0.70901	61.922	67.891	20	18		
		*F*_N_	0.97041	116.262	–18.504	20	18		
		*F*_N(best)_	0.99739	109.295	–5.419	21	14.6		
		*F*_A_	0.36191	–55.944	140.714	18			
		*F*_A(best)_	0.36293	–55.924	140.404	16			

a Range corresponds to either lower (L_1_–L_12_) or higher (H_1_–H_3_) concentration series of QS.

b The L_1_ and H_1_ series contain no added absorber (QS only), while the L_8_ and H_3_ series also contain added absorber (QS and PD).

c *F*_AD_ is the best correction (in terms of *R*^2^ values) performed by the AddAbs method within all measured fluorescence values at variable *z*-positions. *F*_0_ is the uncorrected fluorescence data (technically not a “correction type”) measured at the same *z*-position as *F*_AD_. *F*_Z_, *F*_N_, and *F*_N(best)_ are ZINFE, NINFE, and best NINFE correction, respectively. The ZINFE corrections shown are the best corrections (in terms of *R*^2^ values) with a positive slope. The NINFE correction is performed using the same pair of *z*-positions as for the ZINFE method. For H_1_ series, the slope changes in the NINFE method as a result of the numerical optimization of the exponent *N*. The best NINFE correction is the NINFE correction that gives the highest *R*^2^ value out of all pairs of possible *z*-position combinations (also resulting in a negative slope). *F*_A_ is the Lakowicz correction obtained using the uncorrected values (*F*_0_) with the same *z*-position as for *F*_AD_. *F*_A(best)_ is the *F*_A_ correction that gives the best linearity (in terms of *R*^2^) from the fluorescence data sets at all measured *z*-positions.

d Percentage deviation of the slope from the ideal value, defined as *b* % = (1 – *b*) · 100%. Values closer to zero indicate a smaller deviation from the ideal value (*b* = 1).

e Limit of detection (α = β = 0.05); the values were normalized as percentage of *c*_max_. Values closer to zero indicate higher sensitivity. Negative LOD values are physically meaningless and are the result of a negative slope obtained after numerical optimization of the exponent *N* (NINFE method).

f Absorbance values at excitation (λ_ex_) and emission (λ_em_) wavelengths normalized to optical path length *l* = 1 cm. Values were estimated from the measured absorbance values of the stock solutions or their diluted aliquots.

g Concentrations of QS were estimated from the absorbance at excitation λ_ex_ = 345 nm, *ε*(QS, 345 nm)= 5700 M^–1^.

h Illustrative example of nonlinear fitting using a single-exponential fit with the formula *F*_exp,norm_ = *a* + *b* · ln(*c*_norm_) for the L_1_ titration (see Section 6 in the Supporting Information).

### Low Fluorophore Concentrations (L_1_–L_12_)

For the L_1_–L_12_ concentration series (*A*_345nm,QS,1cm_ = 0.201–2.01), the global *R*^2^ optimum was obtained for the L_8_ titration which contained *A*_345nm,PD,1cm_ = 19.66, as shown for T plates in Figure 2, top, and for NT plates in Figure S8. The best AddAbs IFE correction (*F*_AD_ data, red symbols) gave excellent results: *R*^2^ = 0.9996, *b* % = 1.20%, LOD % = 2.00% for T plates and *R*^2^ = 0.9999, *b* % = 0.30%, LOD % = 1.25% for NT plates. These values are better than the benchmark nonlinear fit of uncorrected fluorescence (*R*^2^ = 0.9966, *b* % = 1.45%, and LOD % = 5.56, T microplates only, Figure S37). For comparison, the uncorrected fluorescence parameters values are *R*^2^ = 0.9463, *b* % = 21.27%, LOD % = 25.24% for T plates and *R*^2^ = 0.7464, *b* % = 43.95%, LOD % = 61.76% for NT plates.

The best ZINFE correction (Figure 2, *F*_Z_ data, blue) and the companion NINFE correction (Figure 2, *F*_N_, light blue), with the numerically optimized exponential term also provided satisfactory linearity (*R*^2^ > 0.998, *b* % ≈ 1% and LOD % < 3.38%) and outperformed nonlinear fit (*F*_exp_ in Table 1) in terms of accuracy (*b* %) but resulted in slightly lower sensitivity (LOD %). However, the *F*_N(best)_ correction obtained by the NINFE method using all available pairs of *z*-positions completely outperformed the nonlinear fit (*R*^2^ = 0.9992, *b* % = 0.95% and LOD % = 2.96%, Table 1). The Lakowicz method (*F*_A_, dark green) gave significantly worse results than the other correction methods or the nonlinear fit. A typical upward curvature corresponding to an overcorrection can be seen in Figure 2, top, indicating that this method is not suitable for the fluorophore concentration range studied.^8^ A comparison of the relevant parameters describing the results of the different IFE corrections is presented in Figure 2, bottom, and in Section 5 in the Supporting Information.

A look at the residual plots shows that for both the T and NT microplates (Figures S32 and S33), the most significant deviation from the linear model is for the IFE-uncorrected titration L_1_. This is to be expected since the nonuniform IFE, which increases with fluorophore concentration, is likely the predominant source of nonlinearity. With the addition of the absorber (PD), both this downward curvature and the absolute values of the residuals decrease. When the amount of absorber is close to the optimum (L_8_), the downward curvature of the data is no longer visible, and the absolute values of the residuals are significantly reduced. Very high concentrations of PD degrade the quality of the obtained corrections and the obtained fluorescence data are again far from linear. We attribute this effect to the significant reduction of the fluorescence signal in extremely absorbing solutions, which in turn leads to a low signal-to-noise ratio. This is evident in the increased residuals for the L_10_ titration (Figure S32, *A*_ex,PD,1cm_ = 38.99), with an additional increase for the L_11_ and L_12_ titrations.

The LOOCV results for the best AddAbs IFE correction are shown in Figures S28 and S29 and Table S8. The obtained variability of *b* % = 0.30% for T plates and *b* % = 0.14% for NT plates shows the robustness and stability of the proposed AddAbs IFE correction method. The results could be further improved by additional optimization of the *z*-position and concentration of the added absorber, as well as by increasing the number of data points and replicates in each titration. Nevertheless, we find these results more than satisfactory to prove the concept of the proposed IFE correction method.

### High Fluorophore Concentrations (H_1_–H_3_)

The IFE corrections for the higher concentration titrations (H_1_–H_3_, *A*_345nm,QS,1cm_ = 3.394–33.94) were performed in the same way as for the lower concentration titrations (L_1_–L_12_), except that the fixed amount of added absorber (PD) was *A*_345nm,PD,1cm_ = 38.99 for H_2_ and *A*_345nm,PD,1cm_ = 77.98 for H_3_. For these titrations, the Lakowicz correction cannot be performed in the usual way due to the extremely high absorbance values at the excitation wavelength, which are not directly measurable in this concentration range. For this IFE correction, we used the estimated values of absorbance at 345 nm to obtain the values of *F*_A_ and *F*_A(best)_ (see details in Section 5.6 in the Supporting Information).

**Figure 3:**
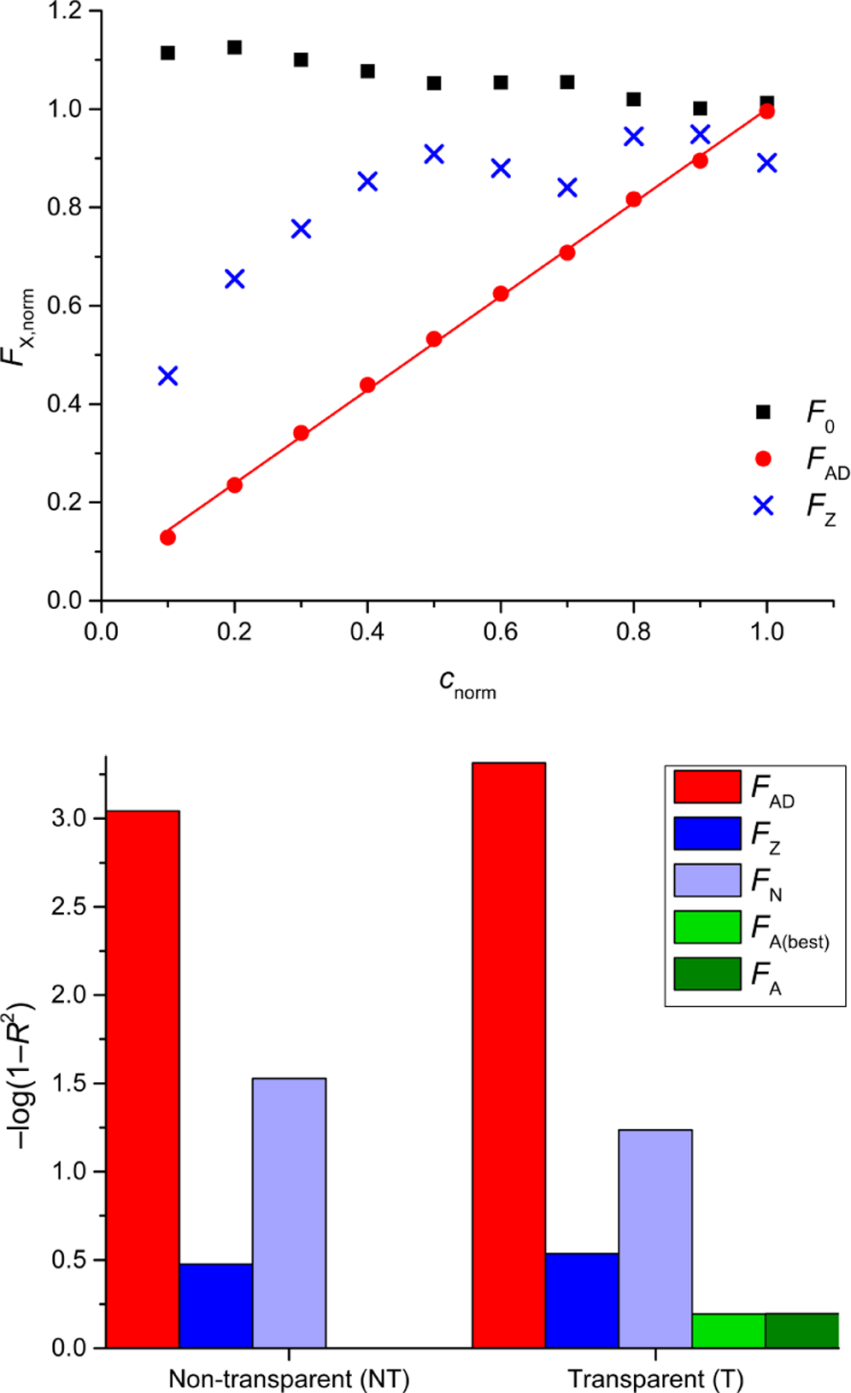
Overview of the different IFE corrections for high fluorophore concentrations, corresponding to the results in Table 1 (T microplates) and Table S4 (NT microplates). The values of *F*_AD_, *F*_Z_, *F*_N_, *F*_A(best)_, and *F*_A_ correspond to AddAbs correction, ZINFE correction, NINFE correction, best Lakowicz correction from the whole set of *z*-positions, and Lakowicz correction obtained at the same *z*-position as *F*_AD_, respectively. **Top:** Normalized IFE-corrected fluorescence data for H_3_ titration in NT microplates. Normalized uncorrected fluorescence *F*_0_ (H_1_ titration) is shown for comparison and the values of *F*_N_ are omitted for clarity. **Bottom:** Values of −log(1 – *R*^2^) obtained for the different types of IFE corrections in T and NT microplates. The logarithmic plot was chosen because the values of 1 – *R*^2^ for the different corrections differ by several orders of magnitude. Greater −log(1 – *R*^2^) values indicate better linearity. Because of the very high absorbance values (not directly measurable) in the solution, the Lakowicz method was performed with the estimated absorbance to obtain the values of *F*_A_ and *F*_A(best)_.

A global *R*^2^ optimum was obtained for the H_3_ titration with *A*_345nm,PD,1cm_ = 77.98, as shown in Figure 3, top, and Table 1. For the T plates, the AddAbs IFE-corrected titration (*F*_AD_ data, red symbols) gave the following parameters: *R*^2^ = 0.9995, *b* % = 0.36%, LOD % = 2.33%, while the parameters for NT plates were *R*^2^ = 0.9991, *b* % = 4.83%, LOD % = 3.19%. The results of the LOOCV analysis (Table S8 and Figures S30 and S31) reconfirmed the remarkable robustness and stability of the proposed AddAbs IFE correction method with an achieved variability of *b* % = 0.35% for T plates and *b* % = 0.49% for NT plates. These results are comparable to the results for the low-concentration series and more than satisfactory to prove the concept of the proposed IFE correction method for the highly concentrated fluorophore solutions.

Some caution is needed in interpreting the results for the H_1_-H_3_ titrations in Table 1. As can be seen in Figure 3, top, the plot of uncorrected fluorescence (*F*_0_ data, black symbols) has a negative slope due to the extreme IFE, which in turn leads to negative (physically meaningless) LOD % values and a very high value of *b* % (>100%). A negative slope can be observed for the *F*_N_ and *F*_N(best)_ corrections, and none of these calibrations have any practical significance. However, looking at *R*^2^ values alone to assess calibration quality could lead to erroneous conclusions, since *F*_0_, *F*_N_, and *F*_N(best)_ all give apparently reasonable values of *R*^2^ > 0.94 and even *R*^2^ > 0.99 for the *F*_N(best)_ correction.

Not surprisingly, the Lakowicz method does not provide a meaningful IFE correction in this concentration range, and the limit for using the ZINFE method also seems to have been reached. Nevertheless, the ZINFE-corrected values in Table 1 are certainly closer to the ideal signal response, but in practice, such inadequately corrected fluorescence signals are not useful. The *F*_Z_ correction shown in Figure 3 has the greatest *R*^2^ value with the positive slope value criterion. A comparison of the relevant parameters describing the results of the different IFE corrections is shown in Figure 3, bottom, and in Section 5 in the Supporting Information, analogous to the results presented previously for the titrations with lower concentrations (L_1_–L_12_).

### Effect of the Added Absorber Concentration and the Sample Geometry (*z*-Position)

Not surprisingly, the ideal amount of added absorber (PD) appears to be a function of the concentration range of the fluorophore (QS). Ideally, the more absorber added, the more linear the results since the contribution of variable absorbance (caused by variable concentrations of the fluorophore) is less significant compared to the contribution of fixed absorbance (caused by a fixed amount of added absorber). The fluorescence is strongly attenuated above a certain amount of the added absorber, which hinders the measurements, as already noted for the titration L_10_ and above. For the entire *z*-position set (except for the highest position, *z* = 34.217 mm), the values of *R*^2^ > 0.99 were obtained for titrations L_5_–L_9_ (for both T and NT microplates). The effect of the amount of added absorber on the shape of the fluorescence titration measurement curves can be seen in Figures S10–S13.

The strongest fluorescence intensities are obtained at a *z*-position interval of 17–19 mm, regardless of the type of microplate used, as shown in Figures S4–S7. On the other hand, the fluorescence signal measured at the highest *z*-position (*z* = 34.217 mm) is about 2–3 orders of magnitude weaker than the corresponding signal obtained at *z* = 18 mm. At very high *z*-positions, the focal point of the incident light beam is relatively high above the surface of the liquid in the microplate well. Presumably, this means that a significant portion of the excitation light does not reach the microplate well in the first place, which in turn leads to a reduction in the intensity of the emitted light. The cumulative effect of a high amount of absorber and a high *z*-position can lead to a significant decrease in the measured signals. Based on these observations, it seems that the best (widest) applicable absorber concentration interval is achieved for the *z*-position that gives the strongest fluorescence signal. Optimization of the *z*-position can be achieved by measuring the fluorescence of the pure fluorophore solution as a function of *z*-position and then estimating the maximum of this function. Modern microplate readers, including the one used in this work, allow automatic optimization of the *z*-position. The data for all titrations are shown in Table S5, and the comparison of all results obtained with the AddAbs correction is shown in Figures S16–S27.

### Advantages and Possible Drawbacks

A major advantage of the proposed AddAbs IFE correction method is that the IFE-corrected data are measured directly, i.e., the data are obtained by a single fluorescence measurement per sample. Most other correction methods require the application of some kind of mathematical procedure to obtain the IFE-corrected values, resulting in more complex error propagation (see details in Section 7 in the Supporting Information). The proposed AddAbs method should also be able to remove the adverse (and likely variable) effects of other potential chromophoric substances present as impurities in the samples during fluorescence measurements. If such impurities are not excessively absorbing, their contribution to the final IFE-based quenching will be insignificant.

**Figure 4:**
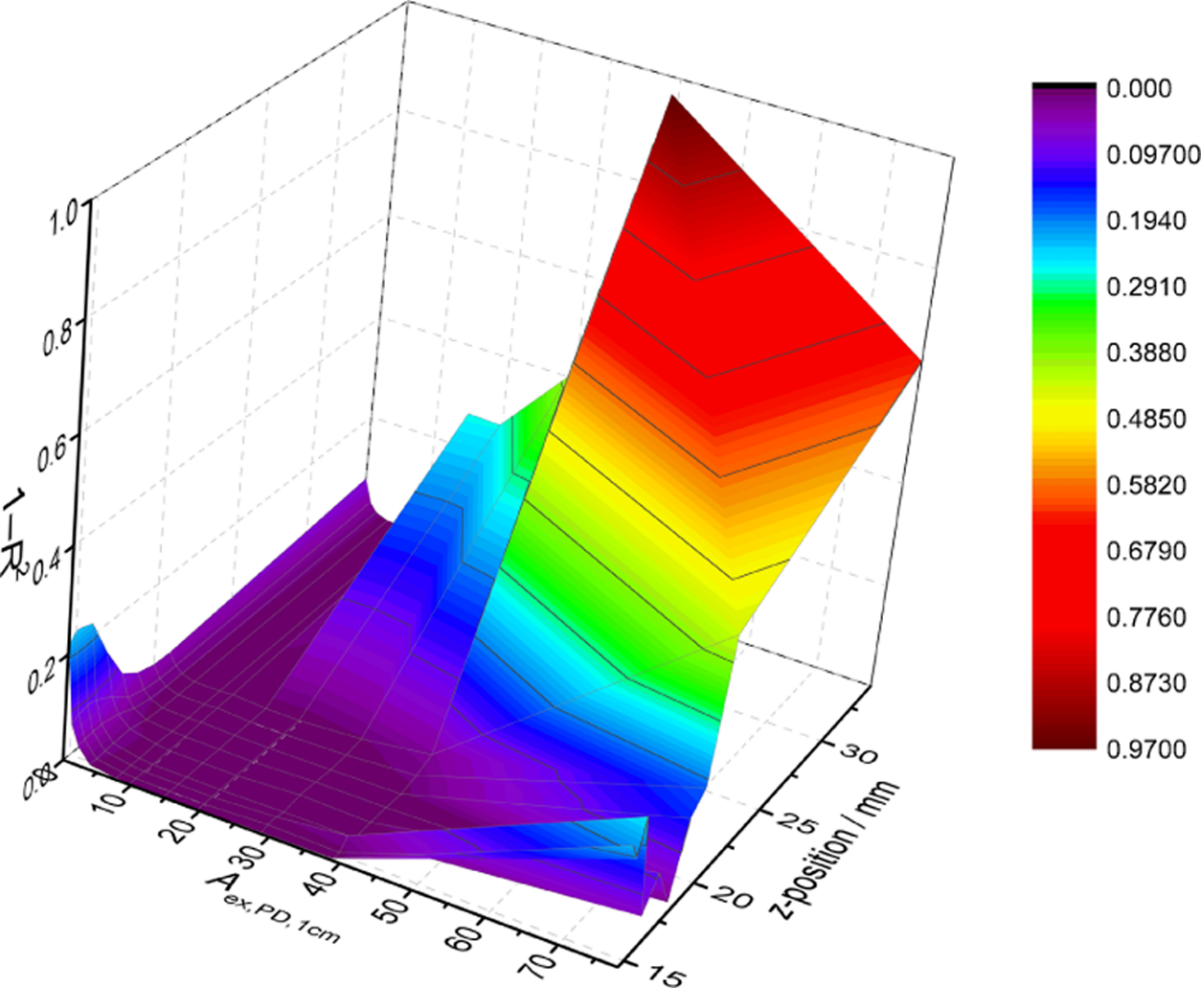
3D representation of the obtained 1 – *R*^2^ results for the titrations (L_1_–L_11_) in the T microplate. For clarity, titration L_12_ was removed from the plot due to the very high concentration of added PD (*A*_ex,PD,1cm_ = 811.7). The dependent variable 1 – *R*^2^ is a function of two independent variables: the *z*-position and the absorbance of the added PD. Deep purple zones indicate a region of very linear IFE corrections.

Another advantageous feature of the AddAbs method is that satisfactory corrections are obtained over a wide range of absorber concentrations. This can be easily observed by looking at the 3D plots (Figures 4 and S20), where the 1 – *R*^2^ values are plotted as a function of *z*-position and absorber concentration (absorbance). The deep purple zones in Figure 4 represent regions that provide high-quality IFE corrections. The fact that we obtained acceptable corrections over the maximum adjustable *z*-position range of the instrument is a strong indication that the acceptable corrections should be obtained with any microplate reader, including those with fixed *z*-position. If possible, the *z*-position should be optimized for the desired sample volume by measuring fluorescence as a function of *z*-position and estimating the approximate maximum.

The AddAbs method can find its potential use in the quantification of proteins in biochemical systems using strongly absorbing detergent-based buffers with SDS and/or NP-40 and commonly used compounds such as NDSB and Triton X-100.^12,13^ These compounds have high molar absorbance coefficients at 280 nm, the typical wavelength for protein excitation. Such a high optical density resulting from the sample matrix itself may make quantification based on absorbance impossible. Nevertheless, in this scenario, the highly absorbing sample matrix can be considered as an inherent added absorber contributing to a linear fluorescence response. This strategy of an inherent added absorber can potentially be used in a variety of fluorimetry experiments to study chemical equilibrium and/or kinetics. Such experiments should be designed to primarily vary the amount of the less absorbing reactant(s) while keeping the concentration of the strongly absorbing reactant(s) approximately constant. This approach minimizes the variability of the optical density of the sample and effectively attenuates IFE.

The major drawback of the AddAbs method is probably the possibility of a chemical reaction between the fluorophore and the absorber. The simplest solution to the problem of reactivity would be to select another (nonreactive) absorber from the plethora of possible candidate chemicals. The ideal absorber should itself be a nonfluorescent substance and should not suppress the fluorescence of the analyte by the IFE-independent mechanisms such as collisional, static, or FRET quenching. In our experience, such unwanted quenching can often be caused by various transition-metal absorbers, such as Cu^2+^ and Co^3+^. A practical review of the principles and differentiation methods for various types of fluorescence quenching mechanisms can be found in a comprehensive review by Panigrahi and Mishra.^5^

Good absorber candidates should also exhibit high absorbance at the excitation and/or emission wavelength(s) of the fluorescent analyte, which depends primarily on good solubility in the chosen solvent and a high molar absorbance coefficient. If both the pIFE and sIFE in the analyte solutions are significant, the ideal absorber solution should have high absorbance in both the excitation and emission regions of the spectrum. This can be achieved by using a single chemical (preferably) or by premixing the two different compounds (one compound to enhance the pIFE, another to enhance the sIFE). The use of multiple absorbers can probably often be avoided because the overlap between the emission spectrum of the fluorophore and the absorber spectrum counteracts this effect by reducing absorbance variability and thus eliminating the sIFE, depending on the magnitude of the Stokes shift. For fluorophores with a relatively large Stokes shift, such as QS in this work, the possible strategy might be to measure at lower emission wavelengths. For pure QS, there is virtually no sIFE at the emission maximum around 450 nm, but if the measured samples contain variable absorbing impurities in this range, a shift to a lower wavelength where an absorber makes a significant spectral contribution might be a good strategy.

Another potential disadvantage arises from the fact that a certain volume of absorber solution must be added to the samples (both for calibration and for measurement of the unknown concentration). It is undeniable that each addition of an additional volume also introduces an additional experimental error. In the experiments we performed, this effect seems to be insignificant due to the high precision of the titrator module used and the relatively low dilution factor (maximum 37 μL of added absorber in 200 μL of sample). However, such dilution of the analyzed fluorophore should not be a problem since the same amount of absorber is added to each solution used to generate the calibration curve and the measured IFE-corrected values can be used directly to estimate the concentration of the fluorophore.

When IFE is corrected by diluting highly concentrated samples with significant IFE, dilution factors must be very high to properly correct IFE. Extrapolation to the zero-dilution volume is likely to be inappropriate in this case, as the extrapolation would extend very far beyond the collected data range. However, the proposed AddAbs method can be used to dilute aliquots of the unknown sample at different dilution ratios but achieving the same final absorber concentration and total volume of final solutions. In this case, IFE should be approximately the same for all samples due to the same amount of absorber present, and extrapolation to the zero-dilution ratio could be performed as shown in eq 4, where *V*_AD_ is the volume of added absorber; *V*_I_ is the initial volume of aliquot sample; *F*_D_ is the IFE-corrected fluorescence measured in a sample diluted with the added absorber; and *F*_UD_ is the estimated IFE-corrected relative fluorescence in the undiluted sample.
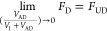
4

### Combining the AddAbs and ZINFE/NINFE Correction Methods

We applied the ZINFE/NINFE methods to make additional IFE corrections for titrations containing a small amount of added absorber (PD) to try to correct for residual nonlinearity. As can be seen from the data in Figures S14 and S15, the ZINFE/NINFE method is able to provide an acceptable linearization for the titrations with an insufficient amount of PD absorber. This demonstrates the robustness of the ZINFE/NINFE methods and proves that an additional correction can be successfully performed when the AddAbs method is performed inadequately for some reason, i.e., when a lower amount of absorber is added than is required for an optimal correction. There may be a physical limit, such as a lower solubility of an absorber combined with an extreme concentration of the fluorophore (analyte), that makes it impossible to fully correct the nonlinearity with the AddAbs method alone. In such cases, a combination of two methods may be required.

ZINFE/NINFE corrections (eqs 2 and 3, respectively) were performed for the L_1_–L_9_ and for the H_1_–H_3_ titrations only. In the case of L_10_–L_12_, negative baseline-corrected fluorescence values were obtained for some *z*-positions, probably because of a low signal-to-noise ratio due to the high amount of added absorber and nonoptimal *z*-positions. If either *F*_0(*z*1)_ or *F*_0(*z*2)_ is negative, the *F*_0(*z*1)_/*F*_0(*z*2)_ ratio will be negative. In this case, eqs 2 and 3 contain a negative base of the exponential function raised to a noninteger exponent, leading to errors in numerical operations. As shown in an earlier section of this work, the ZINFE/NINFE method does not provide quality corrections for the highly concentrated fluorophore without an added absorber (results shown in Table 1 for H_1_ titration). However, the addition of a small to moderate amount of absorber could provide data suitable for the application of the ZINFE/NINFE corrections and extend their applicability and the concentration range of applicable absorbers. Numerical results of the combined corrections can be found in Table S7.

### Applicability of the AddAbs Method to Conventional Spectrofluorometers with Detection at 90° Angle in Rectangular Cuvettes

A notable difference between fluorescence measurements made with conventional 90° angle configuration and those made with a microplate reader is that for fluorescence measurements in rectangular cuvettes, the excitation light must first reach the approximate center of the cuvette and the emitted light must then travel from that region to the detector. Since IFE-based quenching is exponential, it can be assumed that for optically very dense samples, the amount of light reaching the detector may be insufficient to obtain a measurable fluorescence signal.

To investigate this, we performed preliminary measurements with a 90° angle sample geometry using a spectrofluorometer with an actinic LED light source (Olis, USA; see details in Section 8.1 in the Supporting Information). The LED peak wavelength of λ_ex_ ≈ 360 nm proved to be suitable, even though it provided excitation light in the spectral region with an optical density about 20% lower compared to the excitation maximum for QS (345 nm). With this setup, the AddAbs IFE correction method provided satisfactory results for both the low (*R*^2^ > 0.992, *A*_345nm,max,1cm_ = 1.841, *A*_360nm,max,1cm_ = 1.466) and high (*R*^2^ > 0.981, *A*_345nm,max,1cm_ = 24.35, *A*_360nm,max,1cm_ = 19.39) QS concentration series, as shown in Figures S43 and S44. Rotating the cuvette with an optical path length of 10 mm × 2 mm (Hellma, Germany) by 90° increased the sensitivity of the measurements by reducing the optical path of the excitation light needed to reach the center of the cuvette from ≈5 mm (for low QS concentration series) to ≈1 mm (for high QS concentration series).

The observed loss of measurement sensitivity caused by the addition of the absorber is less pronounced when measurements are made with the microplate reader. This is likely because the most optically dense regions are able to produce a measurable fluorescence response in the top-reading mode (i.e., when the optical element used for both excitation and emission is located above the sample), which contributes significantly to the overall signal obtained in the microplates. Since the regions around the spectral maxima exhibit the largest concentration-dependent IFE variability, the IFE-uncorrected fluorescence profiles obtained in microplates show a larger deviation from linearity than the profiles obtained in the cuvette (Figures S43–S46). Based on this observation, we consider that front-face fluorescence spectroscopy^6,14^ is likely to be more effective than the traditional 90° angle setup when using the AddAbs method in rectangular cuvettes. This is mainly because the geometry of the front-face fluorescence spectroscopy is very similar to the measurements in the microplate reader (Figures S40–S42).

## Conclusions

The introduced AddAbs method successfully corrected the nonlinear fluorescence concentration response caused by both pIFE and sIFE by increasing IFE over the entire calibration range to compensate for the nonuniform quenching caused by varying fluorophore concentration. For the lower fluorophore concentration range (*A*_345nm,max,1cm_ = 2.02), both the AddAbs and ZINFE/NINFE methods gave very good results (*R*^2^ > 0.998), whereas the commonly used Lakowicz correction method did not, although it offered a significant improvement over the uncorrected data. However, only the AddAbs method was able to provide satisfactory corrections (*R*^2^ > 0.999) for very concentrated fluorophore solutions with extreme pIFE. The linear fluorescence response was extended to over 97% of the concentration range (LOD % = 2.33%) with *b* % = 0.37% deviation of the calibration slope (T microplates), demonstrating the sensitivity, accuracy, and robustness of the method. Slightly lower sensitivity and accuracy were obtained for NT microplates (LOD % = 3.19% and *b* % = 4.82%), but overall, the results were satisfactory for both types of microplates (*R*^2^ > 0.999, in both the lower and higher concentration ranges). This could be a potentially cost-saving solution because, unlike various IFE correction strategies, the AddAbs method does not require absorbance measurements that might require much more expensive UV–vis–transparent microplates. The proposed method should also be applicable in cases where fluorophore solutions are contaminated with variable amounts of other chromophores since the variability of IFE caused by such contaminants should also be significantly reduced. The method can also be used for measurements in cuvettes on a conventional fluorimeter with a 90° angle configuration. However, for successful measurements, it may be necessary to adjust the excitation wavelength to a range with lower optical density and/or to reduce the effective optical path length by selecting a suitable cuvette.

The optimal amount of absorber added appears to be a function of the concentration of the fluorophore and the *z*-position. However, a wide range of absorber concentrations gave satisfactory results, suggesting that only a rough approximation of the absorber concentration is required before using this method. We obtained satisfactory results for a wide range of adjustable *z*-positions, which is a strong indication that the proposed method can also be used for measurements utilizing microplate readers with fixed *z*-positions, which could be an advantage over the ZINFE/NINFE methods that require an instrument with adjustable *z*-position. We also show that the ZINFE/NINFE method can be used to further correct fluorescence data obtained by the AddAbs method with an insufficient amount of added absorber.

The true limit for the AddAbs method in terms of fluorophore concentration was apparently not reached and may be higher than *A*_ex,1cm_ = 33.94. As far as we know, the highest upper limit for the IFE correction was given by Gu and Kenny, who reported a linear IFE-corrected fluorescence up to *A*_ex,1cm_ = 5.3 for solutions exhibiting only the pIFE (*R*^2^ = 0.9998) and up to (*A*_ex,1cm_ + *A*_em,1cm_) = 6.7 for systems exhibiting both the pIFE and the sIFE (*R*^2^ = 0.9991).^15^ This particular method is only applicable to conventional spectrofluorometers with detection at a 90° angle in rectangular cuvettes and requires a dedicated stage for cell shift experiments, separate measurement of sample absorbance, and optional numerical optimization of geometric parameters. The authors reported an accuracy of about 1.5% for their experiments with QS, while additional numerical optimization gave an accuracy of about 0.2%, which is comparable to the accuracy of the AddAbs method.

## Supporting Information Available

Additional experimental details including instrumental parameters, sample preparation, statistical considerations, and results for all data sets (PDF)
