# Supplementary material for: Reducing the Inner Filter Effect in Microplates by Increasing Absorbance? Linear Fluorescence in Highly Concentrated Fluorophore Solutions in the Presence of an Added Absorber
Source: Anal Chem. 2023 Aug 22;95(35):13036–45. doi: 10.1021/acs.analchem.3c01295 (PMC10483458; doi:10.1021/acs.analchem.3c01295)
Supplement: Supplementary file 1 — ac3c01295_si_001.pdf [file ac3c01295_si_001.pdf]

Supporting Information

Reducing the Inner Filter Effect in Microplates by Increasing Absorbance? Linear Fluorescence in Highly Concentrated Fluorophore Solutions in the Presence of an Added Absorber

Tomislav Friganović* and Tin Weitner

Faculty of Pharmacy and Biochemistry, University of Zagreb, Ante Kovačića 1, 10000 Zagreb, Croatia

Corresponding author:

Tomislav Friganović

*E-mail: tomislav.friganovic@pharma.unizg.hr

1 Table of contents

1 Table of contents 2
2 Preparation of the samples 4
2.1 Chemicals used for the experiments 4
2.2 Preparation of the working solutions 4
2.2.1 0.05 M H_2SO_4 4
2.2.2 Quinine sulfate (QS) stock solution in 0.05 M H_2SO_4 4
2.2.3 Potassium dichromate (PD) stock solutions in 0.05 M H_2SO_4 4
2.2.4 Measured absorbances of the titration stock solutions 4
3 Titrations 6
3.1 Preparation of the titration tables 6
3.2 Randomization of the samples in microplates 8
3.3 Generation the method files for the Tecan Spark microplate reader 8
3.4 Description of the titration procedures 8
3.5 Centrifugation of the microplates 9
4 Measurements 10
4.1 General remarks 10
4.2 IFE in the presence of an added absorber 10
4.3 Absorbance measurements 12
4.4 Fluorescence intensity measurements 13
4.5 Data handling and evaluation 14
4.5.1 Data normalization 14
4.5.2 Quality of fit and linearity measures 14
4.6 Method validation 16
5 Results 17
5.1 Uncorrected fluorescence 17
5.2 Uncorrected fluorescence as a function of z-position 19
5.3 IFE-corrected fluorescence 23
5.3.1 Effect of the added absorber concentration 25
5.4 Comparison of IFE correction methods 32
5.4.1 Coefficient of determination, R^2 34

5.4.2 Percent error of the slope of the normalized data, *b* % 42
5.4.3 Percent error of the Limit Of Detection for normalized data, LOD % 44
5.5 AddAbs IFE correction - overview of results for all *z*-positions 46
5.6 Lakowicz IFE correction - overview of results for all *z*-positions 51
5.7 ZINFE/NINFE correction - overview of best results 57
5.8 LOOCV results and residual plots 59
6 Nonlinear fitting examples 66
7 Propagation of error 74
7.2 Addabs IFE correction 74
7.3 Lakowicz IFE correction 74
7.4 ZINFE/NINFE correction 75
8 Preliminary tests of the AddAbs method using the 90-degree angle fluorimeter setup 77
8.1 Testing method 77
8.2 AddAbs IFE-correction results 77
9 References 86
10 Conflicts of interest 86
11 Acknowledgements 86

2 Preparation of the samples

2.1 Chemicals used for the experiments

Concentrated H_2SO_4 (96 %, cat. no. 410261, Carlo Erba Reagents, France), quinine sulfate (QS, 99.0-101.0 %, cat. no. 22640, Sigma-Aldrich, USA), potassium dichromate (PD, 99 %, cat. no. 1112907, Kemika, Croatia), water (double distilled in an all-glass apparatus). All these chemicals were used without further purification.

2.2 Preparation of the working solutions

2.2.1 0.05 M H_2SO_4

0.05 M sulfuric acid was used as solvent in all titration experiments. This solution was prepared by mixing the appropriate amount of concentrated sulfuric acid with water. The required amount of H_2SO_4(conc) was calculated using the values (density and percentage content) declared by the manufacturer on the bottle of the reagent.

2.2.2 Quinine sulfate (QS) stock solution in 0.05 M H_2SO_4

An arbitrary amount of QS was dissolved in 0.05 M H_2SO_4 to give a solution with high absorbance at λ = 345 nm (excitation wavelength used in our fluorescence measurements). Aliquots of this solution were diluted in 0.05 M H_2SO_4 to prepare QS starting solutions for the titration experiments. Absorbance of the resulting stock solution for the low QS concentration series (L) was estimated a to be approximately $A \approx 4$ at $l = 1$ cm, whereas for the high concentration series (H) it was $A \approx 68$, $l = 1$ cm.

2.2.3 Potassium dichromate (PD) stock solutions in 0.05 M H_2SO_4

Saturated potassium dichromate (PD) stock solution (used in the L_{12} series) was prepared by adding the excess amount of solid $K_2Cr_2O_7$ into the 0.05 M H_2SO_4. The mixture was treated in an ultrasonic bath for approximately 30 minutes and the solid residue was removed by filtration. Other PD stock solutions (used in L_2-L_{11}, H_2, H_3) were prepared by diluting the appropriate amount of the saturated PD solution in the 0.05 M H_2SO_4.

2.2.4 Measured absorbances of the titration stock solutions

The absorbance values of the QS and PD stock solutions were measured in a quartz cuvette (l = 1 mm) using a Varian Cary 50 spectrophotometer (Varian, Australia). For the highly concentrated PD and QS stock solutions, diluted aliquots were used for the measurements (dilutions were performed in a single step, except for the saturated L_{12} stock solution, which was diluted x100 in 2 consecutive x10 dilution steps). The absorbances of the stock solutions (λ = 345 nm) were converted to the path length l = 1 cm,

taking into account the dilution factors (dilution of the stock solution in the titration experiments and any dilutions of the stock solution aliquots performed for the purpose of accurate absorbance measurement of highly concentrated solutions). The results are listed in Table S1.

Table S1. Measured absorbance values are shown for each titration stock solution (λ = 345 nm, l = 1 mm). Highly concentrated stock solutions were diluted prior to absorbance measurements.

Titration no.	Measured stock solution A^a λ = 345 nm, l = 1 mm	Dilution factor[b] c_{stock} / $c_{aliquot}$	Volume ratio[c] V_{added} / V_{total}	Recalculated titration A^d λ = 345 nm, l = 1 cm
L_1 / H_1			0	0
L_2	0.406	NO	0.25	1.02
L_3			0.5	2.03
L_4			0.2	3.90
L_5	1.948	NO	0.4	7.79
L_6			0.5	9.74
L_7	1.966	x2	0.375	14.74
L_8			0.5	19.66
L_9			0.185	28.85
L_{10} / H_2	1.560	x10	0.25	38.99
L_{11} / H_3			0.5	77.98
L_{12}	1.623	x100	0.5	811.7
Q_L[e]	0.402	NO	0.05-0.5	0.201-2.01
Q_H[e]	1.697[f]	40	0.05-0.5	3.39-33.94

[a] Measured absorbance at λ = 345 nm. Measurements were performed in a 1 mm quartz cuvette. The baseline (0.05 M H_2SO_4) was subtracted from the spectral data.
[b] Numerical values of the dilution factors are displayed for the PD stock solutions and the concentrated QS solution, which exhibited very high absorbances at λ = 345 nm. These solutions were diluted prior to absorbance measurements to obtain reliable measurements.
[c] Volume ratio is the dilution ratio of the stock solutions in the titration experiment (i.e., titrations L_4, L_5, L_6 were performed using the: 40, 80, and 100 µL of the same PD solution, resulting in volume ratios of: 0.2, 0.4, and 0.5, respectively, for a total volume of 200 µL).
[d] Absorbance values were recalculated to the values corresponding to the optical path l = 1 cm. This is done by multiplying the measured absorbance of the stock solution by the dilution factor (which equals one if no dilution was performed) and by the volume ratio.
[e] QS was present in the same concentration range in each titration with $A_{max} \approx 2$ (l = 1cm) and $A_{min} \approx$ 0.2 (10 % A_{max}) for the lower concentration series (L_1-L_{12}); $A_{max} \approx 34$ (l = 1cm) and $A_{min} \approx 3.4$ for the higher concentration series (H_1-H_3), respectively.
[f] Absorbance was measured in the 1 cm quartz cuvette.

3 Titrations

3.1 Preparation of the titration tables

A total of 15 different titrations were performed for two different ranges of QS concentrations: (i) low QS concentration range (titrations L_1-L_{12}), and (ii) high QS concentration range (titrations H_1-H_3). QS absorbance values at the excitation wavelength (λ_{ex} = 345 nm) for the most concentrated point in each titration are 2.02 for the L_1-L_{12} and 33.94 for the H_1-H_3 titration sets (normalized to 1 cm optical pathlength). The amount of QS in each titration concentration series ranged from 10 to 100% (based on the data point of highest QS concentration). The concentration values were set to be equidistant ($c_{n+1} - c_n = c_{n+2} - c_{n+1}$), therefore for each titration there are 10 points with the variable amount of QS and 1 point containing no QS (blank). The first titrations, L_1 and H_1, did not contain PD, whereas the amount of PD added increases with the titration number (i.e., L_2 contains the least and L_{12} the most PD). The same amount of PD was added to each point in a given titration.

Titration tables with the required volumes to be pipetted were prepared manually in MS Excel (Table S2). 15 titration experiments (L_1-L_{12} and H_1-H_3) were grouped into clusters of 3 titration experiments each. This was done to measure 3 titration experiments (each in triplicate) in a single 96-well plate. Titrations were clustered as follows: **cluster 1** (L_1, L_2, L_3), **cluster 2** (L_3, L_4, L_5), **cluster 3** (L_9, L_{10}, L_{11}), **cluster 4** (L_7, L_8, L_{12}) and **cluster 5** (H_1, H_2, H_3). For each cluster, the titration table contained 3 columns of data for the volumes of QS, 0.05 H_2SO_4, and PD. The titration table for cluster 4 contained 4 columns because 2 different PD stock solutions were used in this experiment.

Each titration point was performed in triplicate, except for the blank sample (performed in duplicate). The number of data points per replicate was 11: 10 points for the QS concentration range and 1 point for the blank sample. For the QS concentration range, which was measured in triplicate and the blank sample in duplicate, the total number is 10 x 3 + 1 x 2 = 32. For a group of 3 titrations, the total number of microplate wells used is 32 x 3 = 96 (full microplate). The blank samples contained 0.05 M H_2SO_4 (L_1, H_1) or PD dissolved in 0.05 M H_2SO_4 (L_2-L_{12}, H_2, H_3). Under the given measurement conditions, these solutions give a much weaker signal than the samples containing only QS. For this reason, we consider it reasonable to record the values of the blank samples in duplicates only.

Table S2. Titration table showing the volumes of all solutions mixed in the titration experiments. The same volume series of the QS stock solutions were used for each titration experiment (the stock solution with the lower concentration for series L_1-L_{12} and the stock solution with the higher concentration for series H_1-H_3). Potassium dichromate (PD) was added in constant volume for each titration (L_2-L_{12}, H_2, H_3), but this volume was not the same for each titration. 0.05 M H_2SO_4 was added to achieve a total volume of 200 μL for each point in each titration.

Titration no.		*V* / μL										
	Quinine sulfate (QS)	0	10	20	30	40	50	60	70	80	90	100
L_1 / H_1	$K_2Cr_2O_7$ (PD)	0	0	0	0	0	0	0	0	0	0	0
	0.05 M H_2SO_4	200	190	180	170	160	150	140	130	120	110	100
$L_{3,6,8,11,12}$ / H_3	$K_2Cr_2O_7$ (PD)	100	100	100	100	100	100	100	100	100	100	100
	0.05 M H_2SO_4	100	90	80	70	60	50	40	30	20	10	0
L_5	$K_2Cr_2O_7$ (PD)	80	80	80	80	80	80	80	80	80	80	80
	0.05 M H_2SO_4	120	110	100	90	80	70	60	50	40	30	20
L_7	$K_2Cr_2O_7$ (PD)	75	75	75	75	75	75	75	75	75	75	75
	0.05 M H_2SO_4	125	115	105	95	85	75	65	55	45	35	25
$L_{2,10}$ / H_2	$K_2Cr_2O_7$ (PD)	50	50	50	50	50	50	50	50	50	50	50
	0.05 M H_2SO_4	150	140	130	120	110	100	90	80	70	60	50
L_4	$K_2Cr_2O_7$ (PD)	40	40	40	40	40	40	40	40	40	40	40
	0.05 M H_2SO_4	160	150	140	130	120	110	100	90	80	70	60
L_9	$K_2Cr_2O_7$ (PD)	37	37	37	37	37	37	37	37	37	37	37
	0.05 M H_2SO_4	163	153	143	133	123	113	103	93	83	73	63

3.2 Randomization of the samples in microplates

The relative positions of samples placed in microplate wells were (pseudo)randomized using the custom Python script. The original titration tables contained the volume of QS in order of ascending concentration. By randomizing the positions, there is no longer a clear concentration trend within the microplate. We consider this a good practice, especially since the titration procedures used are fully automated with a titrator module accessory for Tecan Spark 10M microplate reader. Randomization was performed to reduce potential systematic errors that could arise from (i) irregularities in the microplate (scratches or errors in the production process), (ii) anisotropy of positions (wells in the center and at the edge of the microplate have a slightly different environment), (iii) possible chemical contamination and/or other effects that could have a greater impact on the adjacent positions of the microplate. A total of 5 unique randomization keys (.txt files) were generated, one for each titration cluster. For the duplicated experiments in the UV-vis-transparent (T) and non-transparent (NT) plates, the same randomization key was used for each pair of plates. We believe that for comparison of sets between plates, it is better to have directly transferable spatial positions (i.e., no variability in relative positions of samples between plate pairs).

3.3 Generation the method files for the Tecan Spark microplate reader

Method files generated by the Tecan Spark 10M microplate reader can be exported as .XML files. This great feature allows manipulation of these files with the programming tools available to the researcher. We have developed a Python script that generates the titration experiment by modifying the .XML template file. Since the titrator module for the Tecan microplate reader contains 2 syringes, only 2 different components can be added per experiment. Titration clusters 1, 2, 3, and 5 required the addition of 3 different stock solutions, while cluster 4 required the addition of 4 different stock solutions. For this reason, each titration cluster was divided into 2 separate titration methods: (i) in the first procedure, the volumes of QS and 0.05 M H_2SO_4 are added, and (ii) in the second procedure, PD is added. The Python script loads the .XML template and .xlsx file with up to 2 columns, each column containing the volumes of the compounds to be pipetted (in random order in this case), and then returns the new method file. The generated new method files can then be easily uploaded to the Tecan microplate reader. The alternative to this procedure would be to manually set the volumes of the individual components for each titration - a tedious task that we wanted to avoid.

3.4 Description of the titration procedures

Before performing a particular titration method, the titrator was thoroughly rinsed with the required working solution(s). As mentioned in Section 3.2, SI, each titration cluster was divided into 2 separate titration methods that were loaded into the instrument. For a given plate pair, these method pairs are identical (since the same randomization key was used for each pair). For this reason, the first titration method sequence was performed sequentially for the T and NT plates, and the second sequence was performed thereafter (e.g., cluster 2 was titrated in the first step by adding QS and 0.05 M H_2SO_4, which was performed sequentially for the T and NT plates, after which PD was added to both plates in the same manner in the second step).

Between the different titration steps, the titrator was thoroughly rinsed, first with distilled water and then with the stock solutions for the titration. Rinsing of the titrator is performed as a predefined method in the Tecan microplate reader software. Rinsing with distilled water was always performed several times in succession; the water in the wash compartment was changed after each step. Rinsing with the working solutions was performed twice in succession. Each titration sequence was performed within 5-10 minutes, so that a given titration cluster was titrated in both T and NT plates in about 40 minutes (taking into account the time required for rinsing the titrator).

3.5 Centrifugation of the microplates

After completion of the titration procedure, each pair of plates was centrifuged at 2550 rpm for several minutes using a PlateFuge™ microplate centrifuge (Benchmark Scientific, USA). Visual inspection confirmed the adequacy of this procedure when no bubbles were seen in the solution and no drops were seen at the edges of the microplate wells.

4 Measurements

4.1 General remarks

Absorbance measurements were performed as a single (unmodified) method for all samples in the UV-vis-transparent (T) plates. Fluorescence intensity measurements were always performed with the same *z*-positions and the same excitation and emission wavelengths, but modified with respect to the gain parameter, which is the amplification factor for the photomultiplier tube of the detector. The gain parameter was the same for each pair of microplates (T and NT). For titration clusters 1-4, it was: 70, 85, 110, and 127, respectively. For cluster 5, 2 gain values were used in the measurements, 65 for H_1 and 100 for H_2 and H_3. The maximum adjustable gain on the Tecan Spark M10 is 255, which is significantly higher than the values used in our experiments. Differences in the gain parameters within the titrations should not affect the linearity of the data sets, but only the absolute values of the measured fluorescence intensity (i.e., higher gain results in a stronger fluorescence intensity signal). Temperature variations did not exceed 0.5 °C within the measured titration cluster. Data were exported using a custom Python script that reads in an .xlsx data file containing the measurements and a unique randomization key (.txt file created as described in Section 3.2, SI). The data are reset to the original positions before randomization using the inverse permutation operation. The measured fluorescence data in the unmodified .xlsx output (which is in the form of a matrix of microplate dimensions) are vectorized and the vectors for each *z*-position of the fluorescence measurements are merged into the matrix.

4.2 IFE in the presence of an added absorber

For a pure fluorophore dissolved in a pure solvent and having a strong IFE, the concentration of the unknown sample can be determined either by correcting the IFE and performing a linear interpolation or by applying a nonlinear interpolation to the IFE-uncorrected calibration curve. If the IFE is caused solely by the fluorophore, the concentration of this fluorophore can theoretically be determined by nonlinear interpolation without IFE correction. However, when IFE is enhanced by added chromophores (i.e., impurities), the concentration of the fluorophore is difficult to determine by nonlinear interpolation because the ratio of fluorophore to chromophore is unknown and inconsistent. This means that the quenching caused by IFE is a combined contribution from the self-quenching of the fluorophore and the quenching caused by the chromophore. Obtaining a pure fluorophore spectrum by spectral deconvolution can be problematic if the spectra of the chromophores in solution are not known or if there is significant overlap with the fluorophore spectrum. When analyzing IFE-uncorrected data by nonlinear interpolation, an unknown amount of absorbing impurities can be a problem, since a different nonlinear fluorescence concentration-response curve is obtained for each ratio of fluorophore to impurity. Only if this ratio is known, the appropriate calibration curve can be used.

If you consider the IFE correction in the presence of a fixed amount of additional chromophores (absorbers) using the Lakowicz method (eq 1), the contribution to A_{ex} or A_{em} can be split into 2 terms: (i) $A_{ex/em,I}$ is the intrinsic absorbance of the fluorophore, which is a function of fluorophore concentration

and thus a variable, and (ii) $A_{\mathrm{ex/em,AD}}$ is the absorbance contribution of the additional chromophore (absorber) and thus a constant, since the amount of chromophore is the same for each sample. Since the values of $A_{\mathrm{ex/em,AD}}$ are declared as constants, the exponential part containing these values (C) is also a constant and can be separated by transforming eq S1 into eq S2.

$$F_{\mathrm{A}} = F_0 \cdot 10^{\left(\frac{A_{\mathrm{ex,I}}+A_{\mathrm{em,I}}+A_{\mathrm{ex,AD}}+A_{\mathrm{em,AD}}}{2}\right)} \quad \text{(S1)}$$

$$F_{\mathrm{A}} = F_0 \cdot 10^{\left(\frac{A_{\mathrm{ex,I}}+A_{\mathrm{em,I}}}{2}\right)} \cdot C \quad \text{(S2)}$$

eq S2 shows that the constant amount of absorber acts equally on each point of a fluorophore concentration series and therefore should have no effect on the correction function. This can be represented as multiplication of a vector by a scalar.

What happens if each sample analyzed contains a variable and unknown amount of the light-absorbing impurity(ies)? In this case, both $A_{\mathrm{ex/em,AD}}$ and $A_{\mathrm{ex/em,I}}$ are unknown and different for each sample and only their sum can be directly determined, as opposed to their ratios. Fortunately, their sum is the factor that determines the total IFE, and the intensity of fluorescence is only proportional to the amount of fluorophore. In general, it should be possible to obtain the correct concentration of fluorophore even in such systems by using the various IFE correction methods and subsequent linear interpolations.

4.3 Absorbance measurements

Absorbance spectra were recorded in the UV-vis-transparent (T) plates (black, 96-well, μ-clear, flat bottom, chimney well, cat. no. 655097, Greiner, USA) in the range of 200-1000 nm, in 1 nm increments. Measurements were performed at room temperature using the Tecan Spark M10 multimode microplate reader.

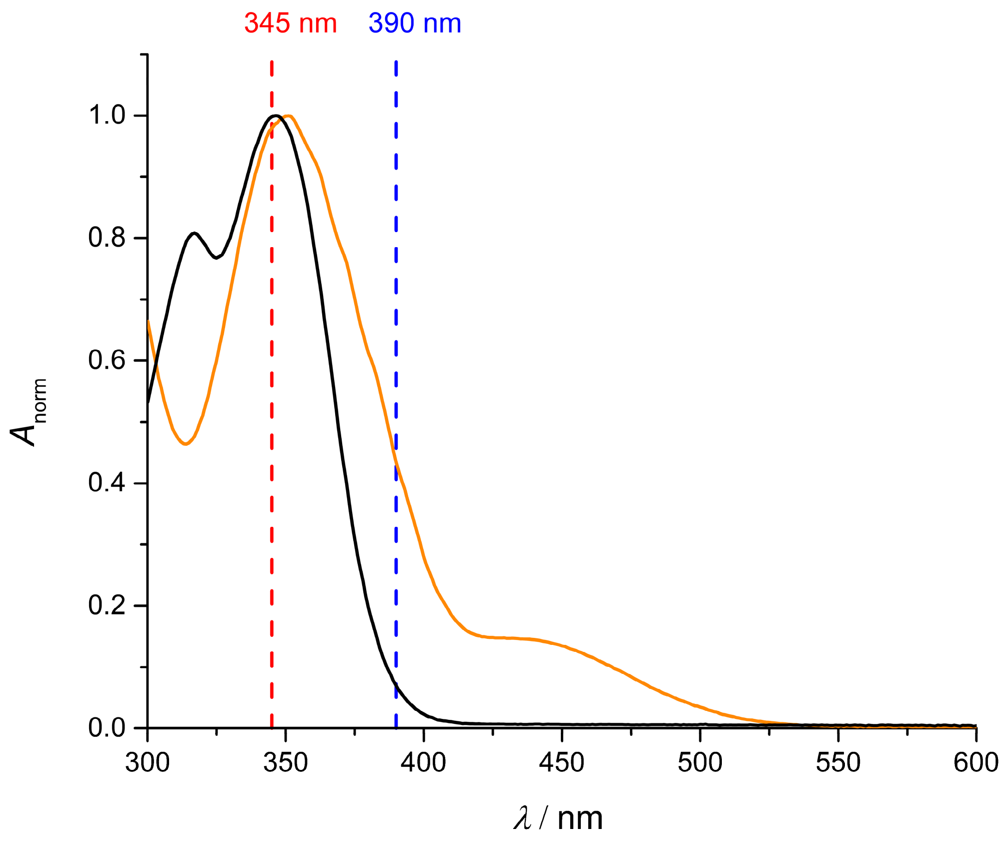

Figure S1. Normalized ($A_{norm,i} = A_i / A_{max}$) absorbance spectra for the solutions of QS (marked in black) and potassium dichromate PD (marked in orange) dissolved in 0.05 M H_2SO_4(aq). The excitation wavelength λ_{ex} = 345 nm and the emission wavelength λ_{em} = 390 nm are marked with red and blue dashed lines, respectively.

4.4 Fluorescence intensity measurements

Fluorescence intensity measurements were performed at room temperature using the Tecan Spark M10 multimode microplate reader. Measurements were performed in fluorescence top-reading mode with a single excitation and emission wavelength (λ_{ex} = 345 nm and λ_{em} = 390 nm). Fluorescence was measured at 10 different *z*-positions for each sample (Table S3), both in the UV-vis-transparent (T) (black, 96-well, μ-clear, flat bottom, chimney well, cat. no. 655097, Greiner, USA) and non-transparent (NT) (black, 96-well, flat bottom, cat. no. 30122298, Tecan, Austria) microplates. The *z*-position value 14.6 mm is the lowest adjustable value for the microplates used, while 34.217 mm is the highest allowable *z*-position.

Although λ_{em} = 390 nm is not the maximum of the QS emission spectrum ($\lambda_{em,max}$ ≈ 450 nm), this wavelength was chosen due to the fact that both QS and PD have non-negligible molar absorbance coefficients at this wavelength, allowing the correction of both pIFE and sIFE to be tested (Figure S1, SI). Emission wavelengths closer to λ_{ex} = 345 nm were not selected because of possible crosstalk interference (i.e., measurement of excitation light as emission fluorescence signal).

Table S3. Values of *z*-positions used for fluorescence intensity measurements and subsequent IFE corrections. Each *z*-position value can be set with an accuracy of up to 1 μm.

***z*-position number**	***z* / mm**
1	14.600
2	15.000
3	15.500
4	16.000
5	17.000
6	18.000
7	19.000
8	20.000
9	21.000
10	34.217

4.5 Data handling and evaluation

4.5.1 Data normalization

The concentration of the fluorophore (independent variable, x-axis) is normalized such that $c_{\text{norm},i} = c_i / c_{\text{max}}$ equals the concentration in a given solution divided by the maximum concentration within the titration (these normalized values range from 0.1 to 1 in all experiments). Some correction procedures can significantly increase the absolute values of fluorescence (e.g., multiplication by the exponential function with a large exponent). Therefore, fluorescence intensity values (dependent variable, y-axis) were normalized by linear interpolation. For each set of data points (each titration), the normalized fluorescence is calculated as $y_i = F_{\text{norm},i} = F_i / (b' + a')$, where b' is the slope and a' is the intercept obtained by linear regression of the baseline-corrected fluorescence (F_i) against the normalized concentration values ($c_{\text{norm},i}$).

Plotting the obtained values of $F_{\text{norm},i}$ against $c_{\text{norm},i}$ gives the new values of slope (b) and intercept (a), which are relevant for assessing of the quality of the correction. We consider this very convenient because the normalized ideal fluorescence signal response (perfectly correlated, no IFE) would have parameters $b = 1$ and $a = 0$, so any significant deviation from the ideal case is easily observed. Interestingly, the parameters a and b for the normalized data are linearly dependent, i.e., $a + b = 1$, so that deviations in both slope and intercept can be expressed by a single parameter.

4.5.2 Quality of fit and linearity measures

(a) Coefficient of determination, R^2

This represents the proportion of the variance of the dependent variable that is explained by the independent variable(s) in a regression model, and is defined as:[1]

$$R^2 = \frac{(\text{cov}(x,y))^2}{\text{var}(x)\text{var}(y)} = \frac{(\text{SS}_{xy})^2}{\text{SS}_{xx}\text{SS}_{yy}} \tag{S3}$$

values closer to 1 indicate a better fit.

(b) Standard error of the estimate, s_y

This represents the measure of variation used to check the accuracy of the predictions made with the regression line, and is defined as:[2]

$$s_y = \sqrt{\frac{1}{(n-2)}\left[\sum_{i=1}^{n}(y_i - \bar{y})^2 - \frac{\left[\sum_{i=1}^{n}(x_i-\bar{x})(y_i-\bar{y})\right]^2}{\sum_{i=1}^{n}(x_i-\bar{x})^2}\right]} \tag{S4}$$

where n is the number of data points for linear interpolation. Values closer to 0 indicate a better fit.

(c) Limit of detection, LOD

LOD is defined as the least amount of a substance that can be distinguished from the blank (i.e. absence of the substance) at a given confidence level, i.e. probability of false positive error (α) or false negative error (β).

The background-corrected signal, $y_{SAMPLE} - y_{BLANK}$, is proportional to the sample concentration c:

$$y_{\mathrm{SAMPLE}} - y_{\mathrm{BLANK}} = b \cdot c \tag{S5}$$

where y_{BLANK} is the signal from the blank sample and b is the slope of the calibration line.

Limit of detection is defined as:[3]

$$\mathrm{LOD} = \frac{ns_y}{b} \tag{S6}$$

where s_y is the standard error of the estimate (eq S4) and n is chosen depending on the required confidence level.

For a chosen confidence level of 5 % (i.e., $\alpha = \beta = 0.05$), the eq S6 is equal to:[1,3]

$$\mathrm{LOD} = \frac{3.3s_y}{b} \tag{S7}$$

For convenient comparison of results, the LOD values obtained for the raw data were normalized as a percentage of the highest concentration of the analyte in the corresponding series (c_{max}), resulting in LOD % values (Table 1 and Table S4), as shown below:

$$\mathrm{LOD}\ \% = 100\ \% \cdot \mathrm{LOD} = 100\ \% \cdot \frac{ns_y}{b} = 100\ \% \cdot \frac{3.3s_y}{b} \tag{S8}$$

(d) Percent error of the slope of the normalized data, *b* %

Considering that the ideal fluorescence signal, which corresponds to the linear relationship between F and A in the absence of IFE, is a line with slope $b = 1$ and intercept $a = 0$ for normalized data, b % value is defined as:

$$b\ \% = (1 - b) \cdot 100\ \% \tag{S9}$$

where b is the slope of the linear regression line for normalized data (see section 4.5.1). Values closer to 0 indicate a better fit.

4.6 Method validation

In a typical quantitative analysis of the fluorophore, a calibration curve is first constructed using a series of measurements made with fluorophore samples of various known concentrations. An interpolation is performed, and the concentration of the unknown sample is estimated using this interpolated mathematical function and the measured fluorescence of the unknown sample. The validity of the linear model describing IFE-corrected fluorescence for a given fluorophore concentration series does not need to be additionally tested based on its firm theoretical background. However, if a fluorophore exhibits concentration-dependent equilibria, it is possible that a nonlinear signal-concentration response is present even in the absence of the IFE. In the case of QS and PD, this does not appear to be the case, as this effect has not been observed by us or by other investigators using the same model compounds.[4,5,6] If an appropriate interpolation model is chosen to describe the system, no significant trends in the residuals should be observed. Plotting the residuals is a very convenient way to visually check whether there are systematic deviations from the model and their magnitude. The residual (r) is defined as the difference between the observed and predicted (expected) values, as shown in eq S10.

$$r_i = y_i - \hat{y}_i \tag{S10}$$

In the case of the ideal IFE-corrected (normalized) data, the parameters of the linear interpolation equation are $b = 1$ and $a = 0$, and therefore eq S10 can be written as eq S11, showing that the residuals can be calculated by subtracting the normalized x_i values from the normalized y_i values.

$$r_i = y_i - bx_i + a \ = \ y_i - 1x_i + 0 \ = y_i - x_i \tag{S11}$$

To test the validity of the proposed AddAbs IFE correction method, we performed the Leave-One-Out Cross-Validation (LOOCV) analysis on 4 data sets corresponding to the best correction obtained for each titration set (L_1-L_{12} and H_1-H_3) in T and NT microplates. The original data for each titration contain $n = 10$ points (averaged triplicate values) and LOOCV is performed for each of the n_i points. Linear least squares interpolation is performed for the n_i-th point, and the remaining $n-1$ points are used for interpolation. The expected value $\hat{y}_i$ is estimated for each point and compared to the measured value y_i.

5 Results

5.1 Uncorrected fluorescence

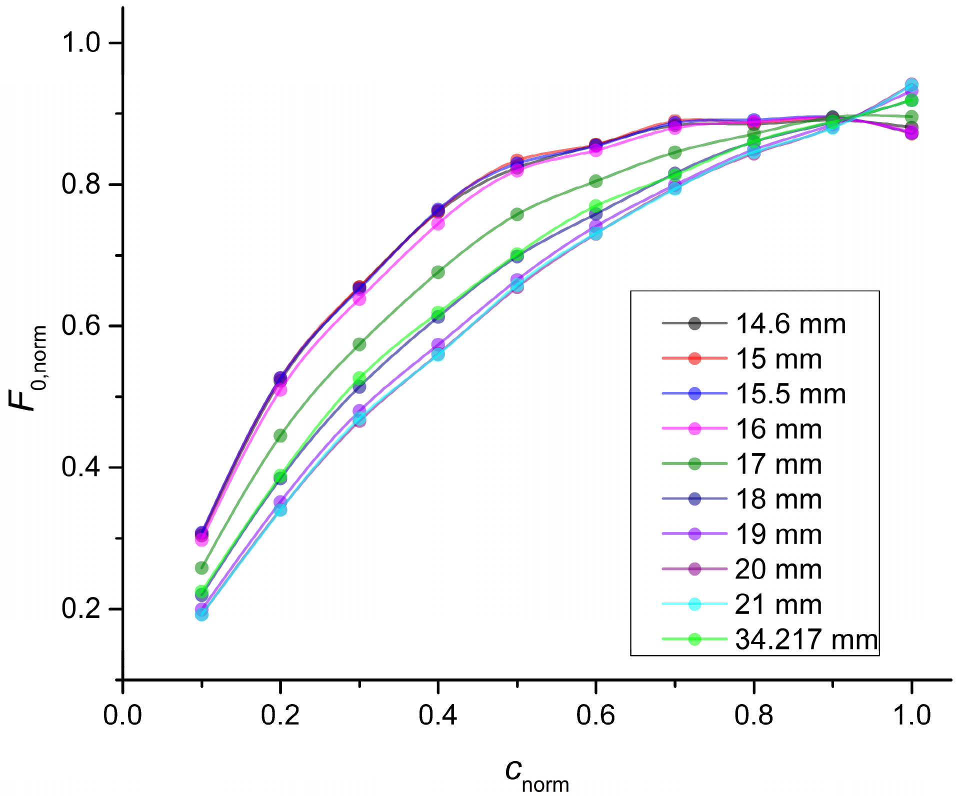

Figure S2. Normalized uncorrected fluorescence intensity values, $F_{0,\mathrm{norm}}$, recorded at 10 different z-positions and plotted as a function of scaled QS concentration, c_{norm}: L_1 titration in non-transparent (NT) microplate. The corresponding data for measurements in UV-vis-transparent (T) microplate are shown in the manuscript, Figure 1, Top.

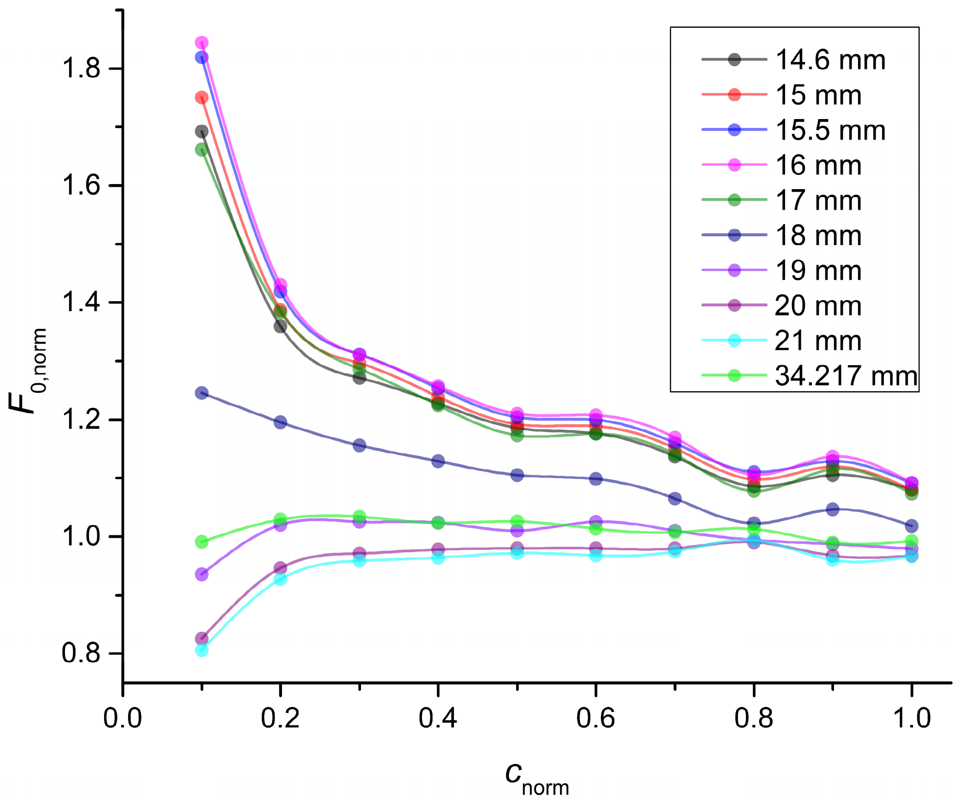

Figure S3. Normalized uncorrected fluorescence intensity values, $F_{0,norm}$, recorded at 10 different z-positions and plotted as a function of scaled QS concentration, c_{norm}: H_1 titration in UV-vis-transparent (T) microplate. The corresponding data for measurements in non-transparent (NT) microplate are shown in the manuscript, Figure 1, Bottom.

5.2 Uncorrected fluorescence as a function of *z*-position

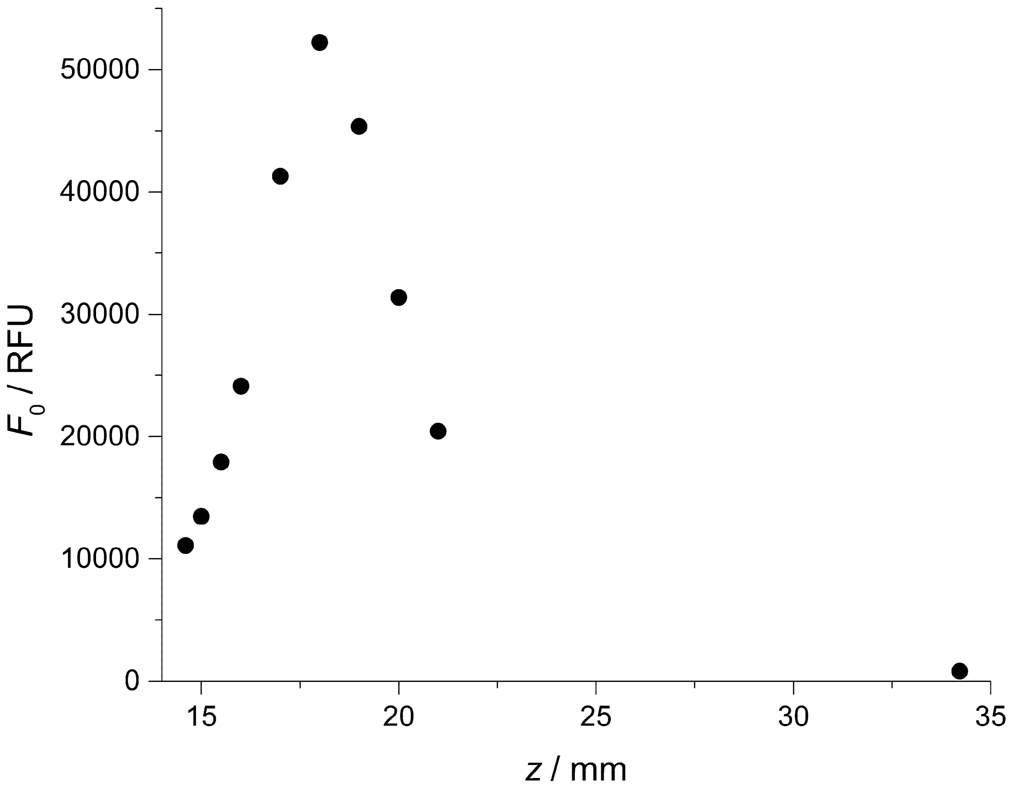

Figure S4. Fluorescence as a function of *z*-position measured for a titration point with the highest fluorophore concentration within the L_1 set in the T microplate (gain = 70).

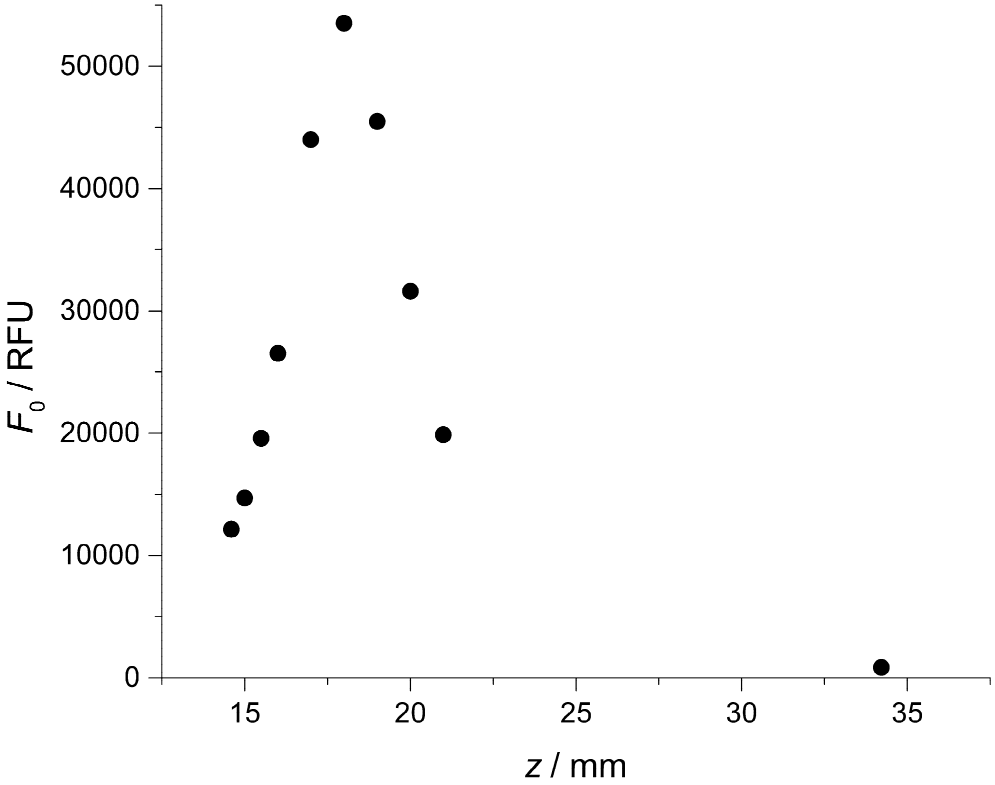

Figure S5. Fluorescence as a function of *z*-position measured for a titration point with the highest fluorophore concentration within the L_1 set in the NT microplate (gain = 70).

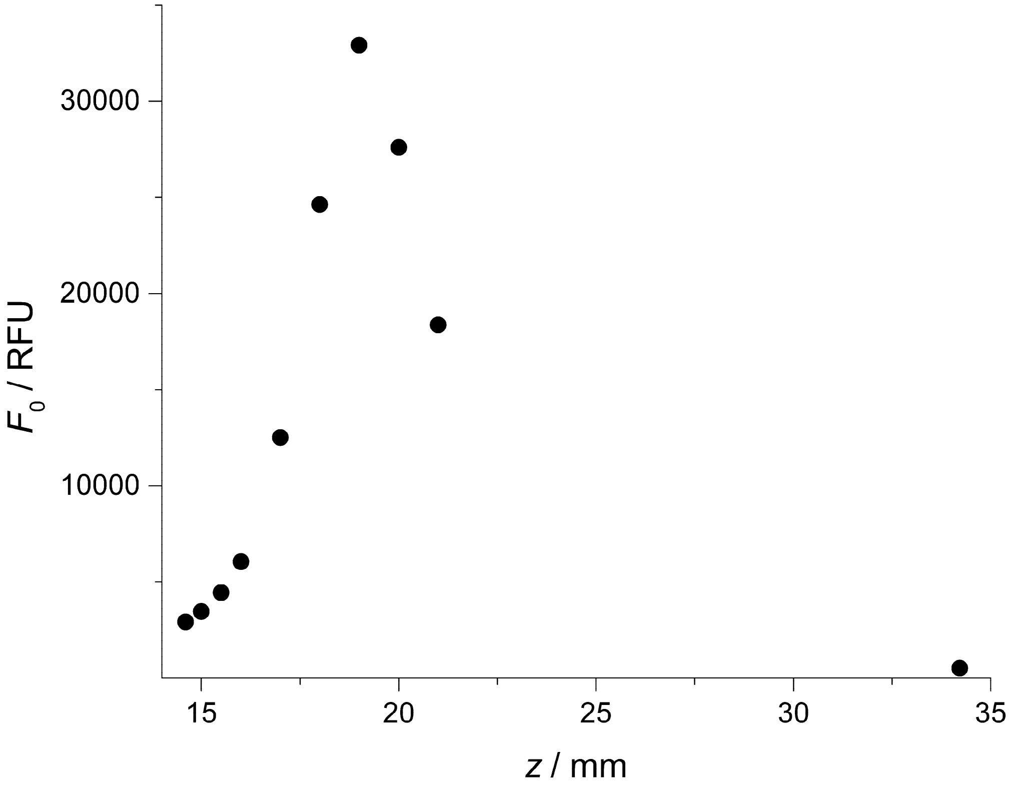

Figure S6. Fluorescence as a function of *z*-position measured for a titration point with the highest fluorophore concentration within the H_1 set in the T microplate (gain = 100).

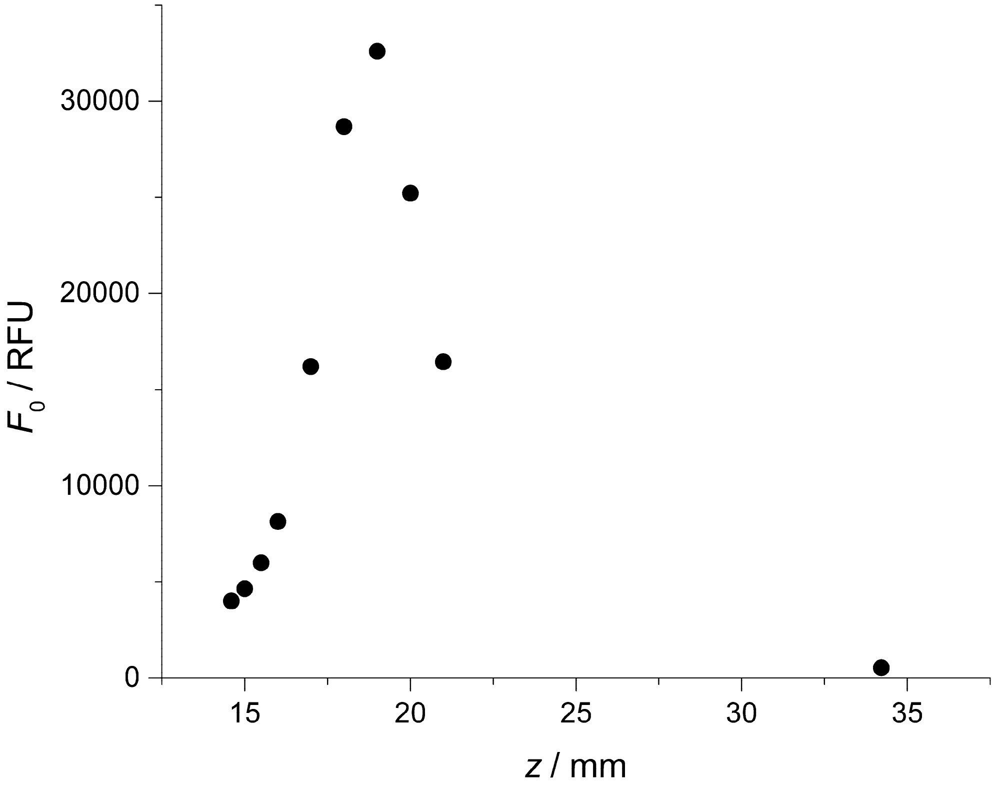

Figure S7. Fluorescence as a function of *z*-position measured for a titration point with the highest fluorophore concentration within the H_1 set in the NT microplate (gain = 100).

5.3 IFE-corrected fluorescence

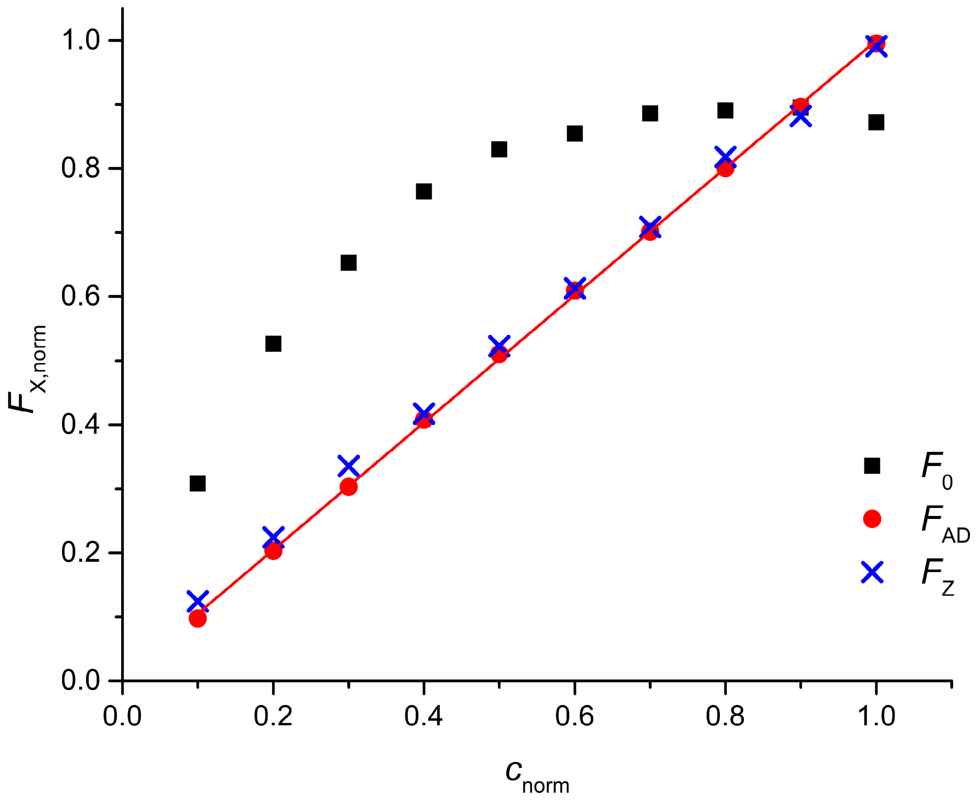

Figure S8. Overview of the different IFE corrections for low fluorophore concentrations corresponding to the results in Table S4 (NT microplates). Normalized IFE-corrected fluorescence data for: AddAbs correction (F_{AD}, L_8 titration), ZINFE correction (F_Z, L_1 titration) and normalized uncorrected fluorescence (F_0, L_1 titration) also shown for comparison. The values of F_N (NINFE correction) were omitted for clarity due to the high similarity with the F_Z values. The corresponding data for measurements in UV-vis-transparent (T) microplate are shown in the manuscript, Table 1 and Figure 2, Top.

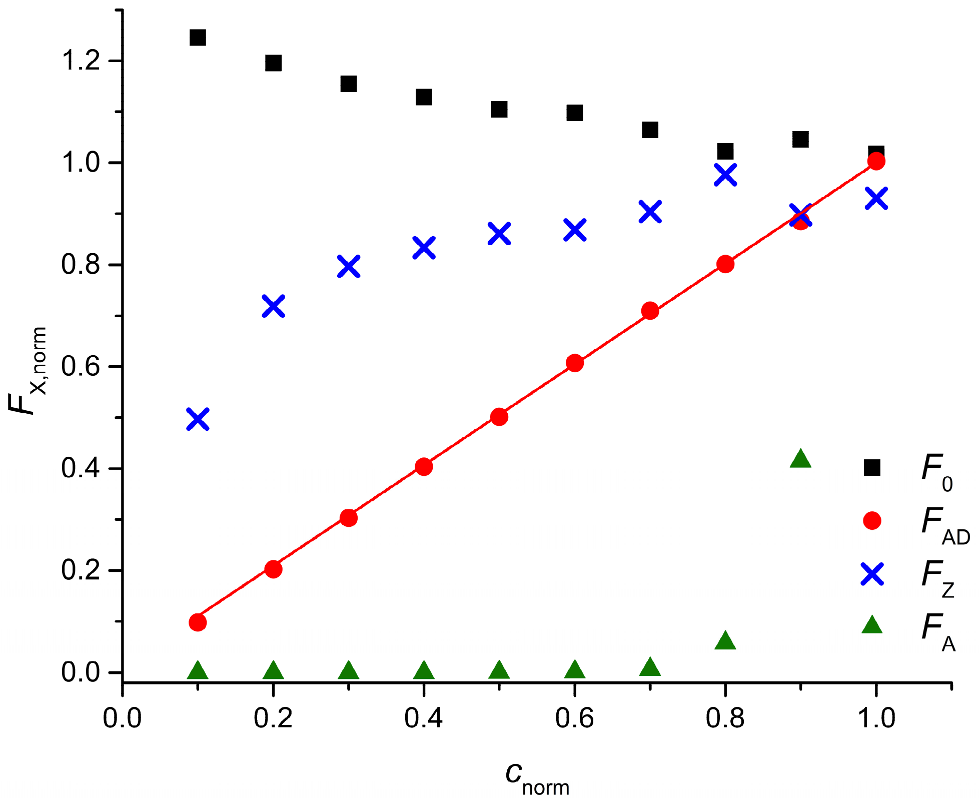

Figure S9. Overview of the different IFE corrections for low fluorophore concentrations corresponding to the results in the manuscript, Table 1 (T microplates). Normalized IFE-corrected fluorescence data for: AddAbs correction (F_{AD}, H_3 titration), ZINFE correction (F_Z, H_1 titration), Lakowicz correction (F_A, H_1 titration) and normalized uncorrected fluorescence (F_0, H_1 titration) also shown for comparison. Values for F_A are based on the estimated absorbance values (see Section 5.6, SI for details), and the point for $c_{norm} = 1$, $F_A = 2.5$ was omitted for better visibility of the remaining data points. The values of F_N (NINFE correction) were omitted for clarity due to the high similarity with the F_Z values. The corresponding data for measurements in UV-vis-transparent (NT) microplate are shown in the manuscript, Table 1, and Figure 3, Top.

5.3.1 Effect of the added absorber concentration

For titrations with low QS concentration, optimal corrections are obtained for L_8 titration and further addition of absorber decreases the quality of corrections. For the titrations with high QS concentration, H_3 titration gives significantly better results than H_2 titration (except for z = 34.217 mm). For both the H_2 and H_3 experiments, the amount of added PD exceeds the optimal amount of PD for the titrations with lower QS concentration (corresponding to the L_{10} and L_{11} series, respectively), confirming that the optimal amount of added absorber is a function of fluorophore concentration.

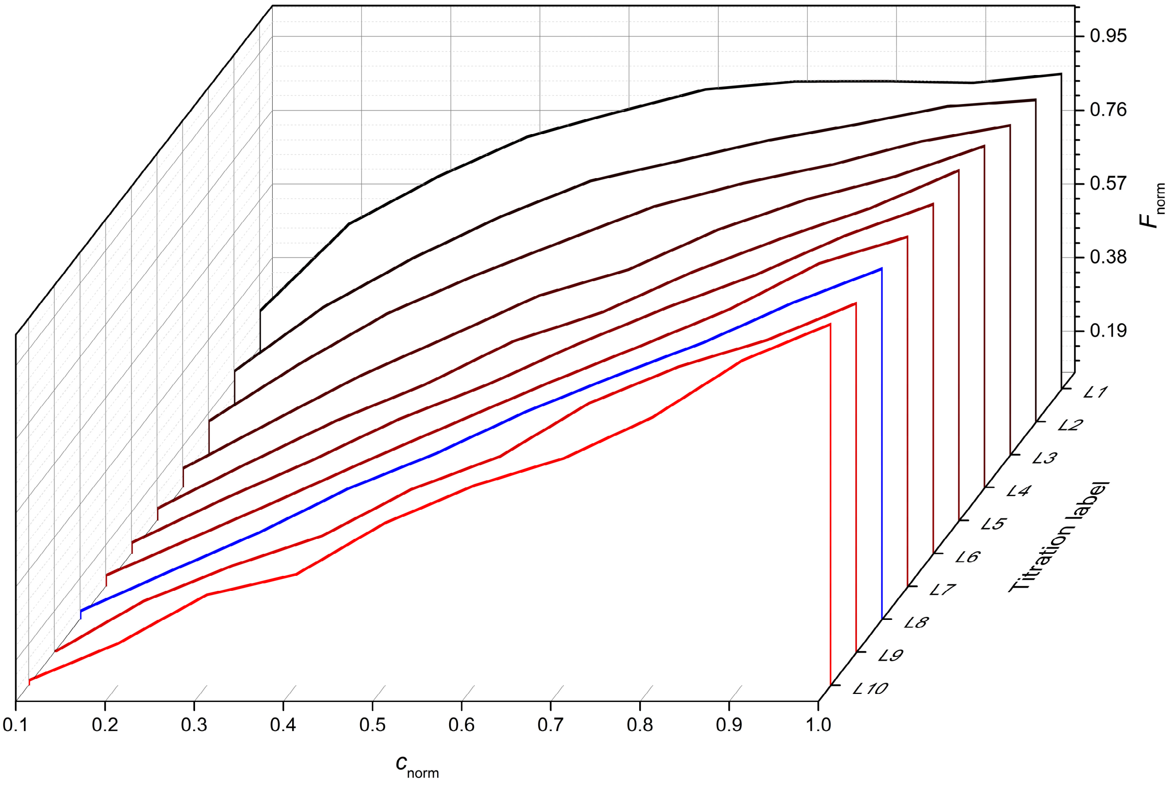

Figure S10. Normalized fluorescence as a function of normalized concentration for the low QS concentration titrations (L_1-L_{12}) in the T microplate. The most linear set is highlighted in blue.

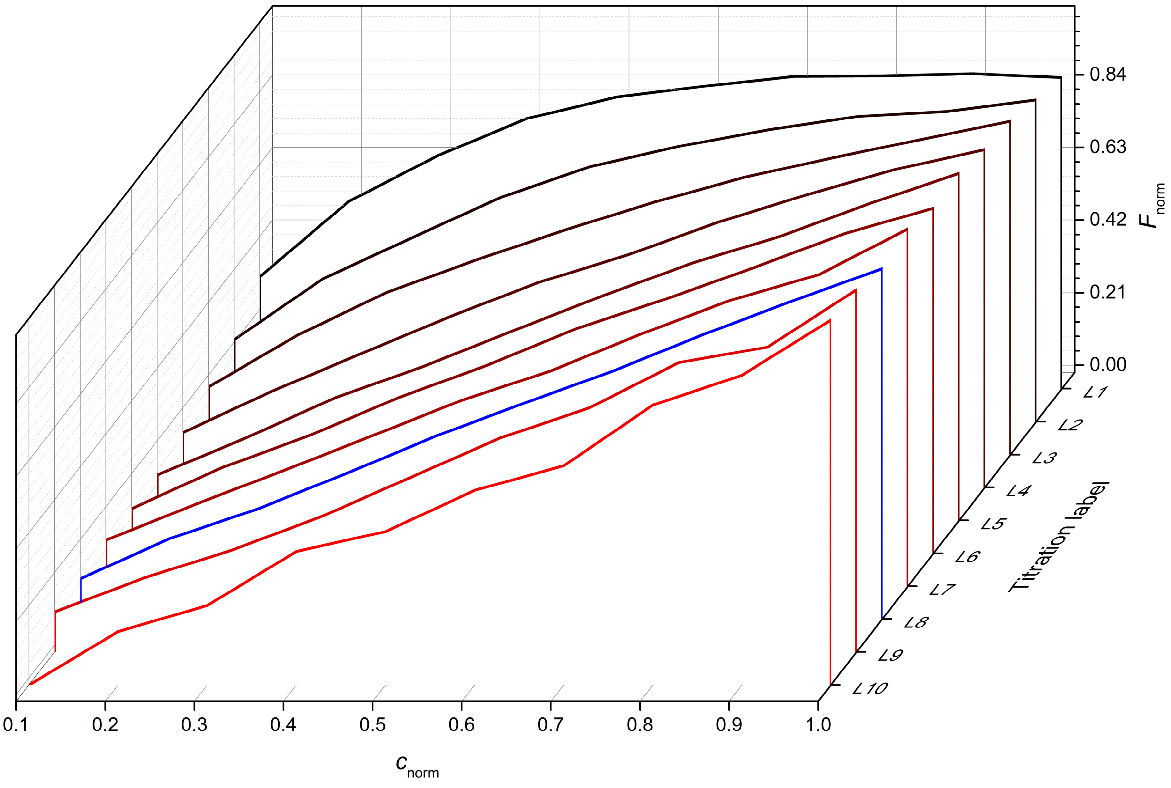

Figure S11. Normalized fluorescence as a function of normalized concentration for the low QS concentration titrations (L_1-L_{12}) in the NT microplate. The most linear set is highlighted in blue.

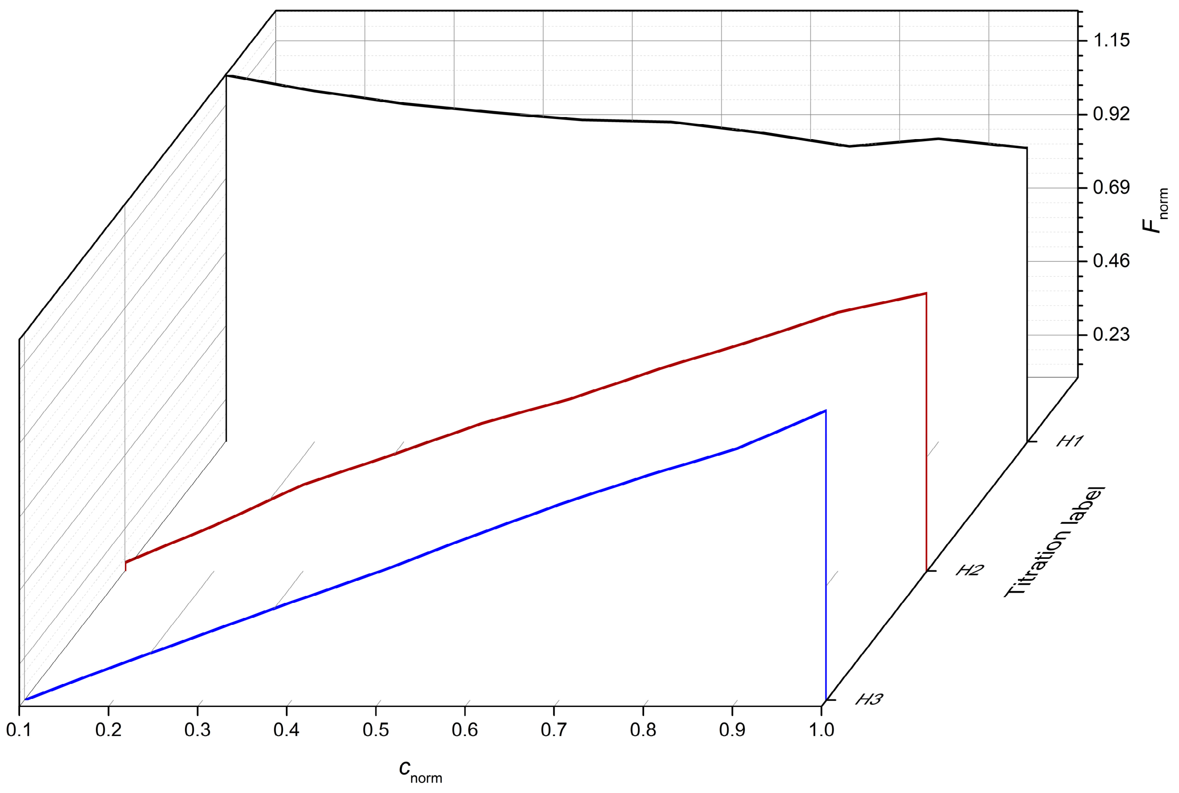

Figure S12. Normalized fluorescence as a function of normalized concentration for the high QS concentration titrations (H_1-H_3) in the T microplate. The most linear set is highlighted in blue.

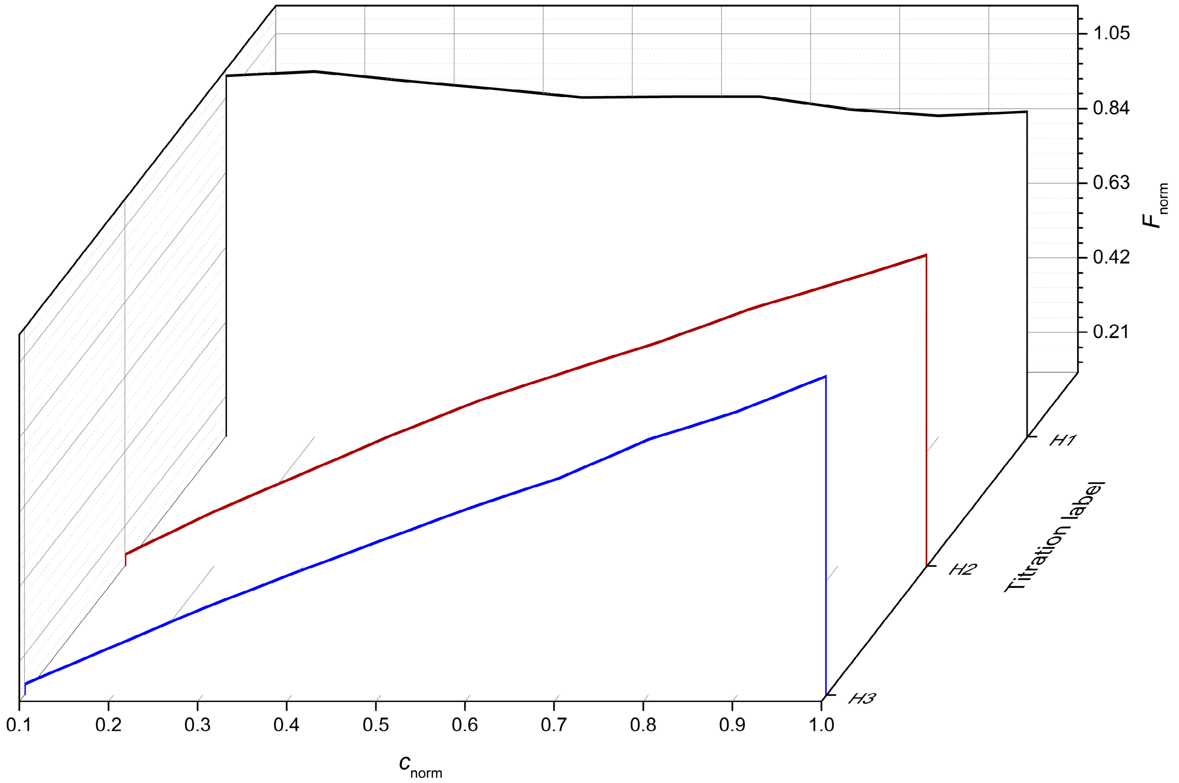

Figure S13. Normalized fluorescence as a function of normalized concentration for the high QS concentration titrations (H_1-H_3) in the NT microplate. The most linear set is highlighted in blue.

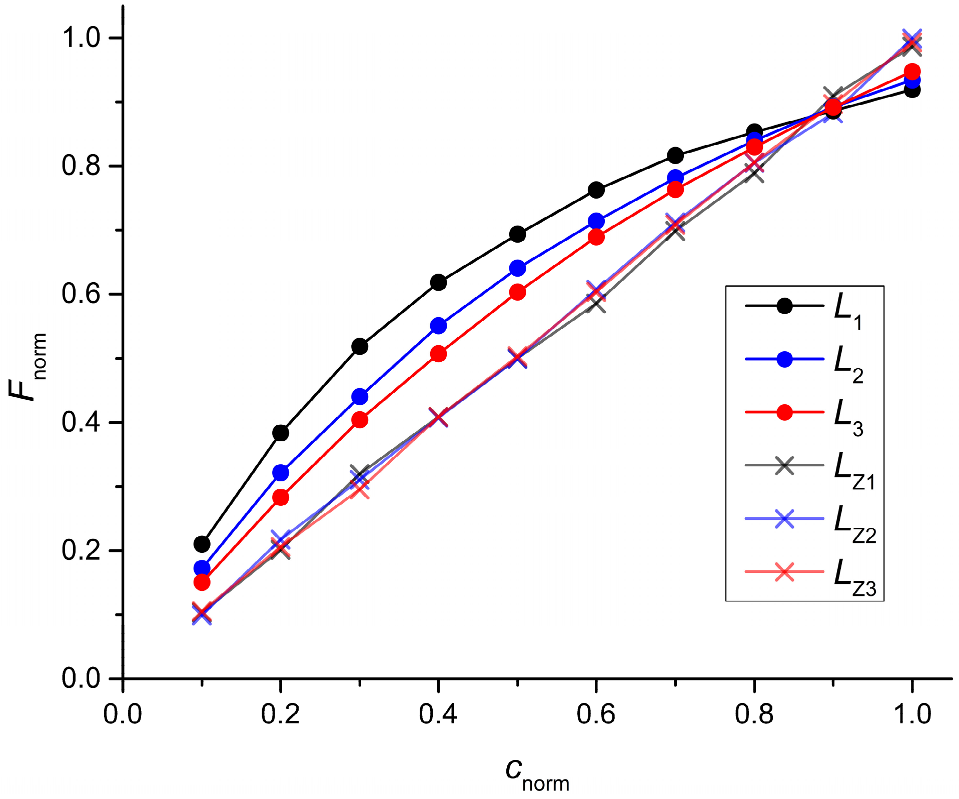

Figure S14. ZINFE (F_Z) method performed on titrations L_1-L_3 in the T microplate.

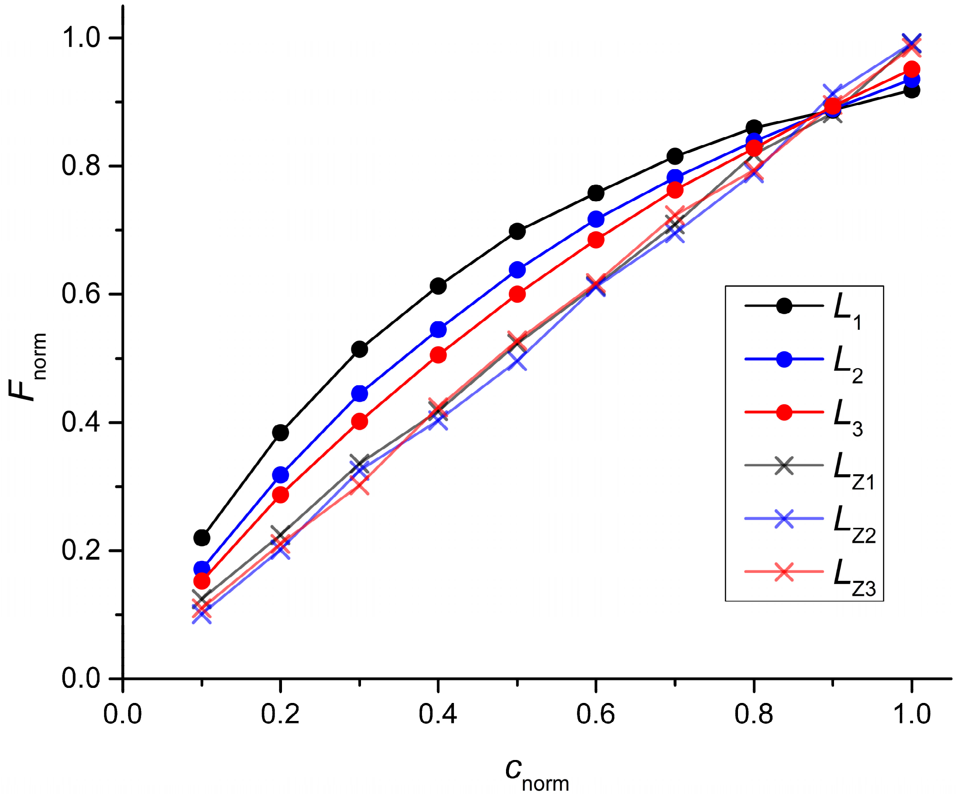

Figure S15. ZINFE (F_Z) method performed on titrations L_1-L_3 in the NT microplate.

5.4 Comparison of IFE correction methods

Table S4. Overview of the least-squares linear fit results for normalized background-corrected fluorescence and absorbance data obtained in non-transparent (NT) microplates.

**Range**[a]	**Series**[b]	**Correction type**[c]	R^2	***b* %**[d]	**LOD %**[e]	z_1 / **mm**	z_2 / **mm**	$A_{max,1cm}$[f] (λ_{ex}, λ_{em})	c_{max}[g] / **mM**
Low	L_8	F_{AD}	0.99986	0.301	1.248	15.5	-	21.67, 7.14	0.353
	L_1	F_0	0.74645	43.953	61.763	15.5	-	2.01, 0.12	
		F_{exp}[h]	0.99473	1.792	5.507	18			
		F_Z	0.99895	3.761	3.433	18	14.6		
		F_N	0.99918	2.324	3.034	18	14.6		
		$F_{N(best)}$	0.99918	2.324	3.034	18	14.6		
High	H_3	F_{AD}	0.99909	4.826	3.194	18	-	111.92, 29.91	5.954
	H_1	F_0	0.92548	113.616	−30.071	18	-	33.94, 2.07	
		F_Z	0.66538	58.591	75.151	20	17		
		F_N	0.94205	110.724	−26.285	20	17		
		$F_{N(best)}$	0.99056	107.481	−10.3477	21	16		

[a] Range corresponds to either lower (L_1 – L_{12}) or higher (H_1 – H_3) concentration series of QS.
[b] The L_1 and H_1 series contain no added absorber (QS only), while the L_8 and H_3 series also contain added absorber (QS and PD).
[c] F_{AD} is the best correction (in terms of R^2 values) performed by the AddAbs method within all measured fluorescence values at variable *z*-positions. F_0 is the uncorrected fluorescence data (technically not a "Correction type") measured at the same *z*-position as F_{AD}.
F_Z, F_N, and $F_{N(best)}$ are ZINFE, NINFE, and best NINFE correction, respectively. The ZINFE corrections shown are the best corrections (in terms of R^2 values) with a positive slope. The NINFE correction is performed using the same pair of *z*-positions as for the ZINFE method. For H_1 series, the slope changes in the NINFE method as a result of numerical optimization of the exponent *N*. The best NINFE correction is the NINFE correction that gives the highest R^2 value out of all pairs of possible *z*-position combinations (also resulting in a negative slope). F_A is the Lakowicz correction obtained using the uncorrected values (F_0) with the same *z*-position as for F_{AD}. $F_{A(best)}$ is the F_A correction that gives the best linearity (in terms of R^2) from the fluorescence data sets at all measured *z*-positions.
[d] Percentage deviation of the slope from the ideal value, defined as $b\ \% = (1 - b)\cdot 100\%$. Values closer to zero indicate a smaller deviation from the ideal value ($b = 1$).
[e] Limit of detection ($\alpha = \beta = 0.05$); the values were normalized as percentage of c_{max}. Values closer to zero indicate higher sensitivity. Negative LOD values are physically meaningless and are the result of a negative slope obtained after numerical optimization of the exponent *N* (NINFE method).
[f] Absorbance values at excitation (λ_{ex}) and emission (λ_{em}) wavelengths normalized to optical path length $l = 1$ cm. Values were estimated from the measured absorbance values of the stock solutions or their diluted aliquots.
[g] Concentrations of QS were estimated from the absorbance at excitation λ_{ex} = 345 nm, ε(QS, 345 nm)= 5700 M^{-1}.
[h] Illustrative example of nonlinear fitting using a single-exponential fit with the formula $F_{norm} = a + b\cdot\ln(c_{norm})$ for the L_1 titration (see Section 6, SI).

5.4.1 Coefficient of determination, R^2

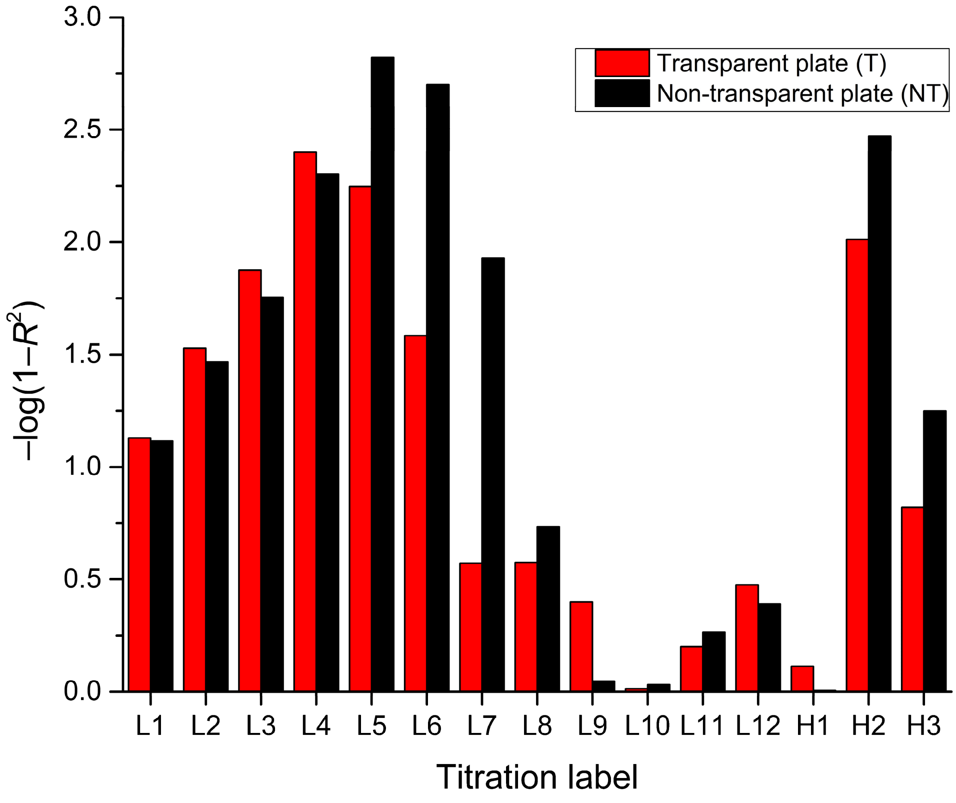

Figure S16. Comparison of $1-R^2$ values obtained for all titration experiments in both the T and NT microplates at z = 34.216 mm. Negative logarithmic values were plotted due to the significant differences in the $1-R^2$ results. Greater values on the y-axis indicate better linearity.

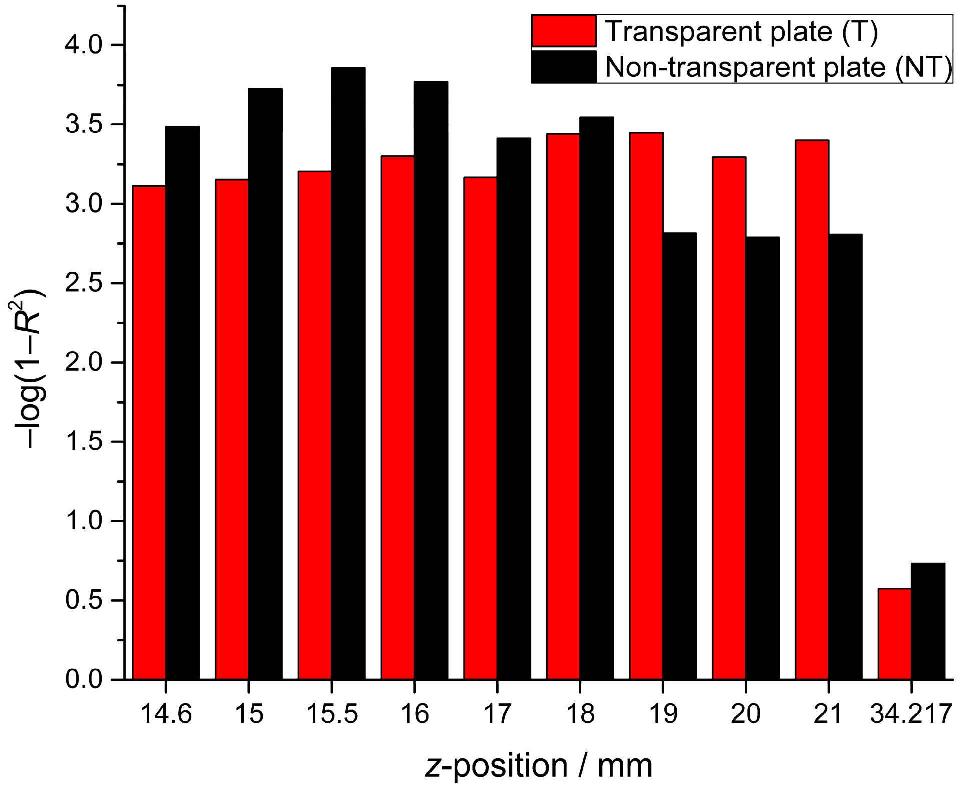

Figure S17. Comparison of $1-R^2$ values obtained for L_8 titration at variable z-position values. Negative logarithmic values were plotted due to the significant differences in the $1-R^2$ results (mainly for z = 34.217 mm). Greater values on the y-axis indicate better linearity.

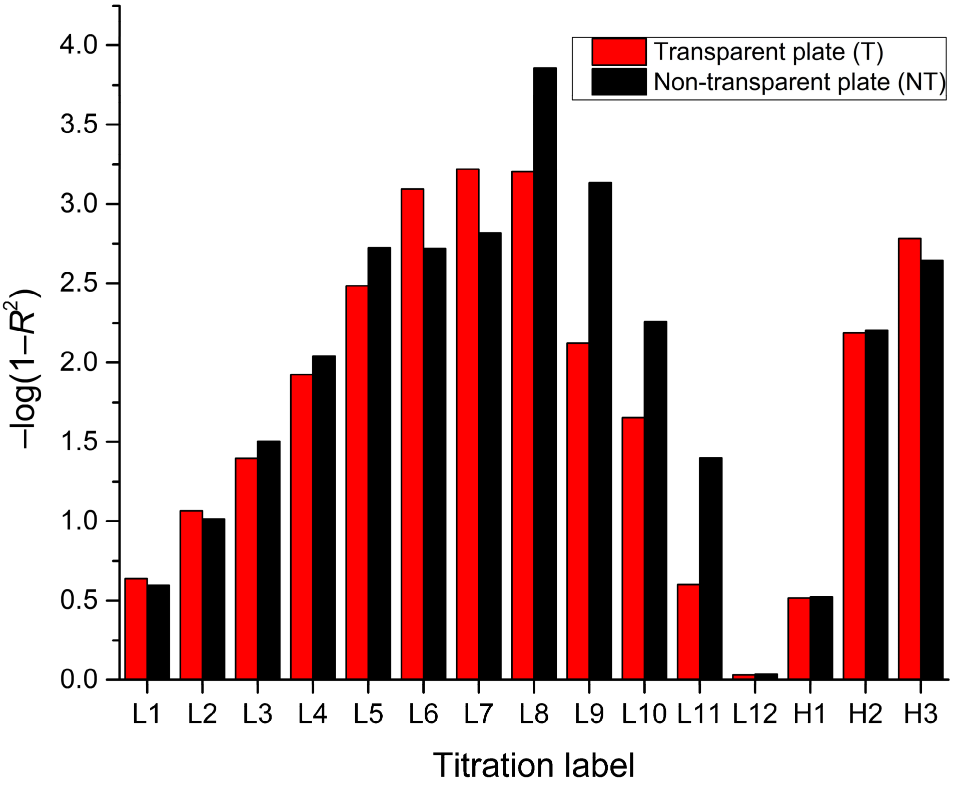

Figure S18. Comparison of $1-R^2$ values obtained for all titration experiments in both the T and NT microplates at z = 15.5 mm. Negative logarithmic values were plotted due to the significant differences in the $1-R^2$ results. Greater values on the y-axis indicate better linearity.

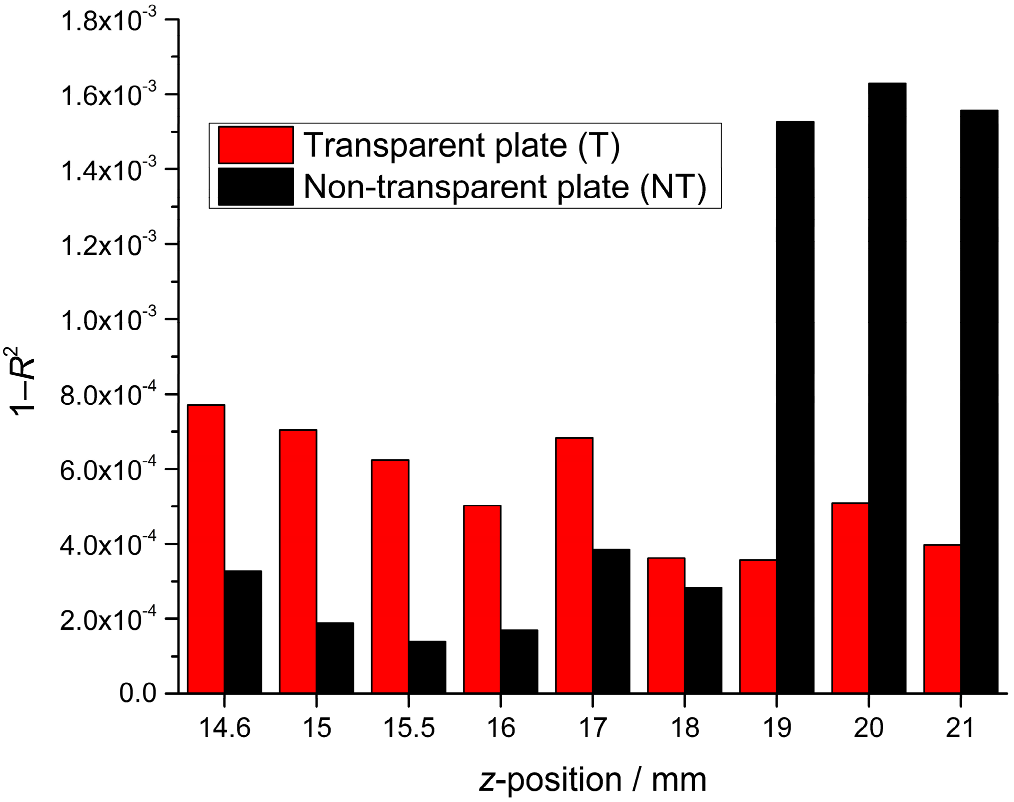

Figure S19. Comparison of $1{-}R^2$ values obtained for L_8 titration at variable *z*-position values, except for $z = 34.217$ mm, as the absolute values are significantly higher. Lower values on the *y*-axis indicate better linearity.

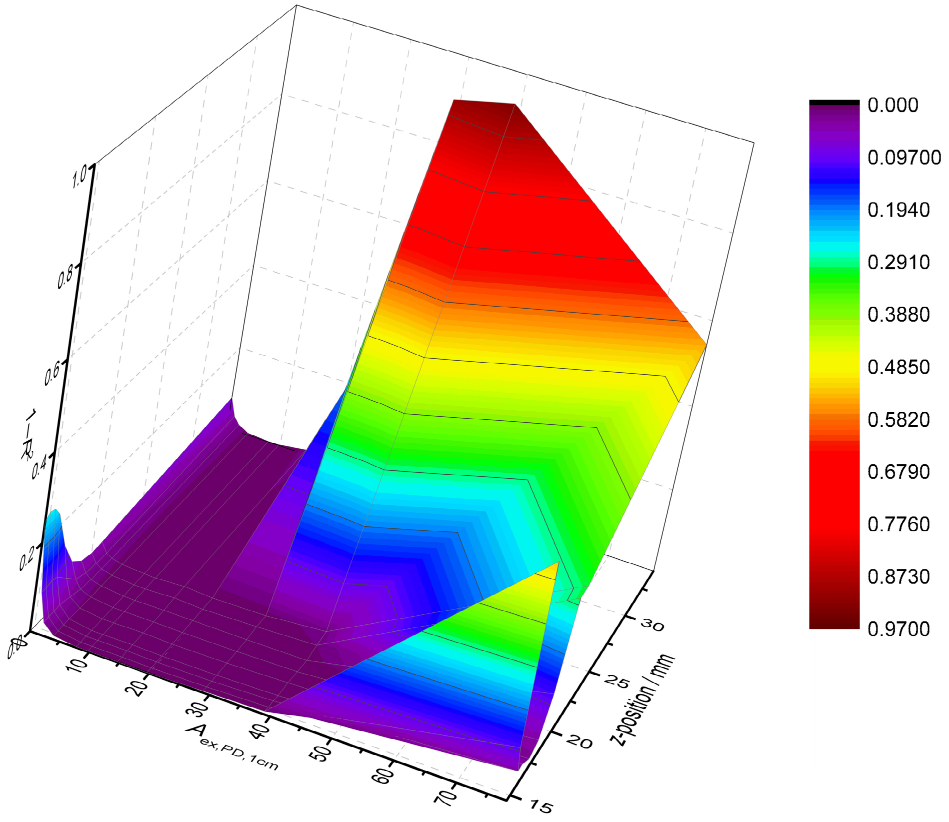

Figure S20. 3D representation of the obtained $1-R^2$ results for the titrations (L_1-L_{11}) in the NT microplate. For clarity, titration L_{12} was removed from the plot due to the very high concentration of added PD ($A_{ex,PD,1cm}$ = 811.7). The dependent variable $1-R^2$ is a function of 2 independent variables: the *z*-position and the absorbance of the added PD. Deep purple zones indicate a region of very linear IFE corrections. The corresponding data for measurements in UV-vis-transparent (T) microplate are shown in the manuscript, Figure 4.

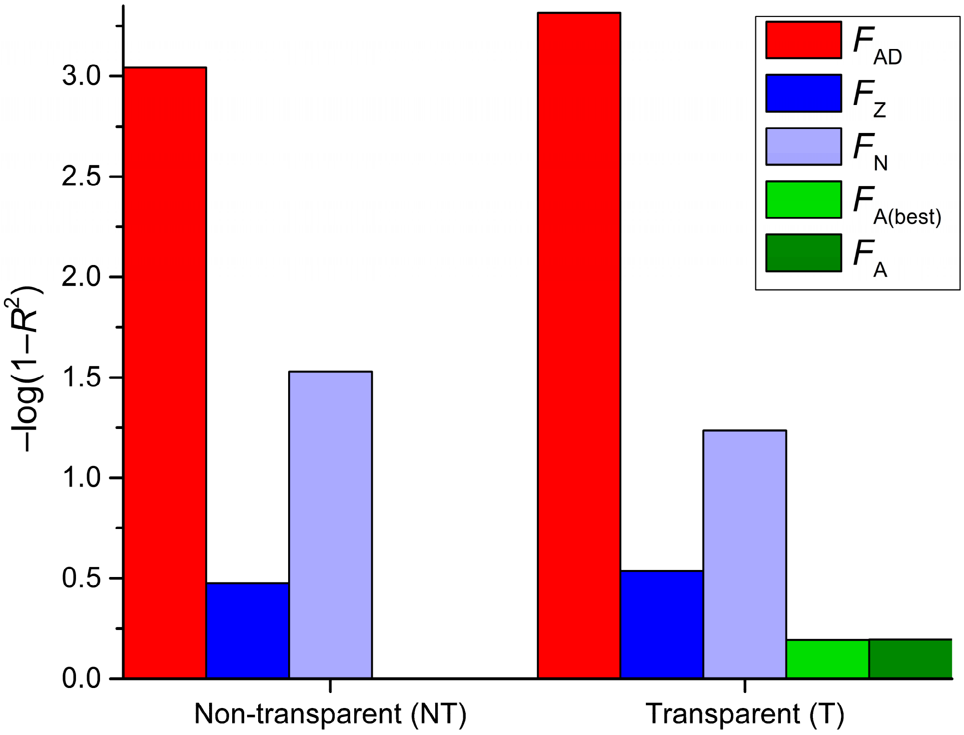

Figure S21. Values of $-\log(1-R^2)$ obtained for the different types of IFE-corrections in T and NT microplates. The values of F_{AD}, F_Z, F_N, $F_{A(best)}$, and F_A correspond to: AddAbs correction, ZINFE correction, NINFE correction, best Lakowicz correction from the whole set of *z*-positions, and Lakowicz correction obtained at the same *z*-position as F_{AD}, respectively. The logarithmic plot was chosen because the values of $1-R^2$ for the different corrections differ by several orders of magnitude. Greater $-\log(1-R^2)$ values indicate better linearity. Because of the very high absorbance values (not directly measurable) in the solution, the Lakowicz method was performed with the estimated absorbance to obtain the values of F_A and $F_{A(best)}$.

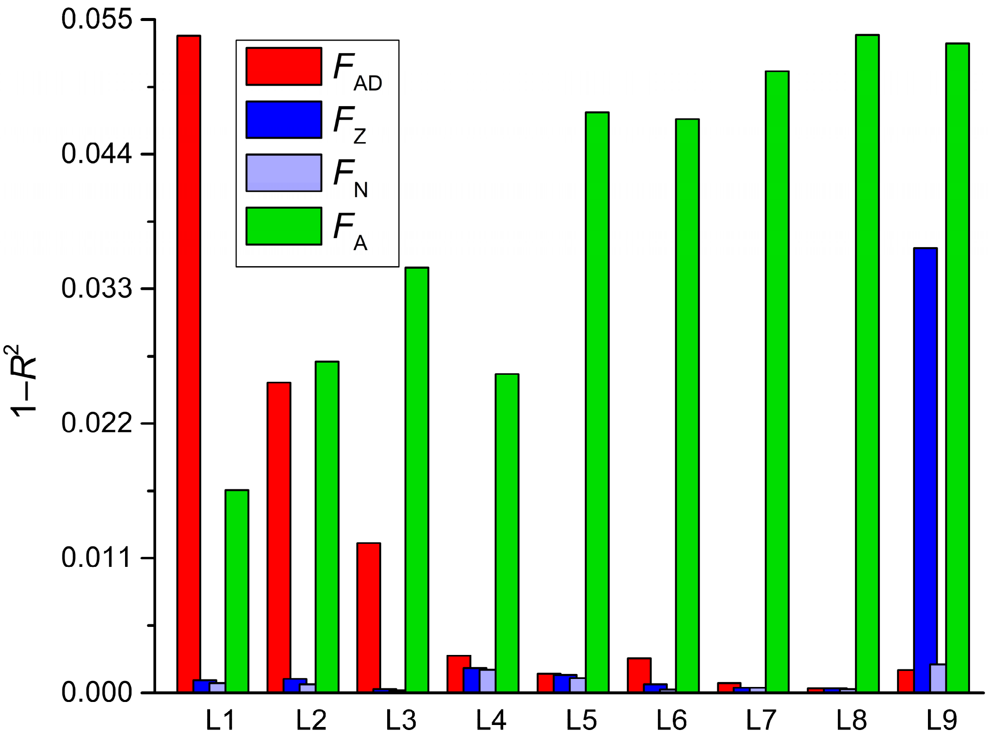

Figure S22. Comparison of calculated $1-R^2$ values in T microplate for different IFE-correction methods performed on the titrations containing the variable amount of absorber and lower concentration of fluorophore (L_1 - L_9). For F_{AD} and F_A, the *z*-position is 19 mm, while the pairs of *z*-positions that give the best corrections for F_Z and F_N are listed in Table S7.

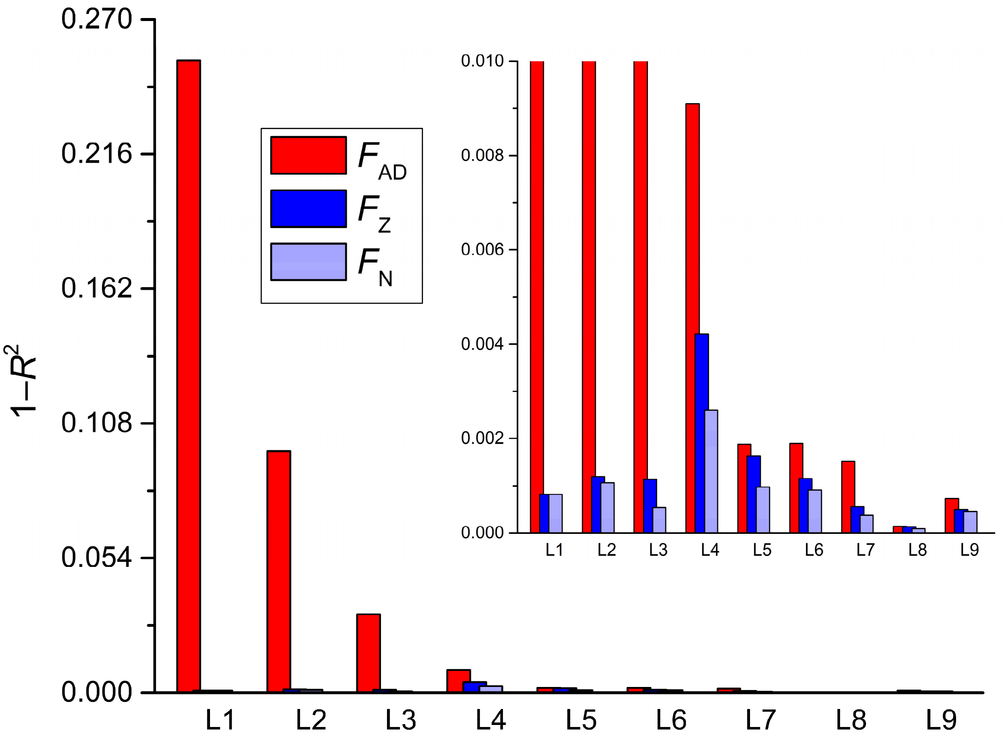

Figure S23. Comparison of calculated $1-R^2$ values in NT microplate for different IFE-correction methods performed on the titrations containing the variable amount of absorber and lower concentration of fluorophore (L_1 - L_9). For F_{AD} and F_A, the z-position is 15.5 mm, while the pairs of z-positions that give the best corrections for F_Z and F_N are listed in Table S7. The inset displays the $1-R^2$ values within a narrower range, improving the visibility of the F_Z and F_N values.

5.4.2 Percent error of the slope of the normalized data, *b* %

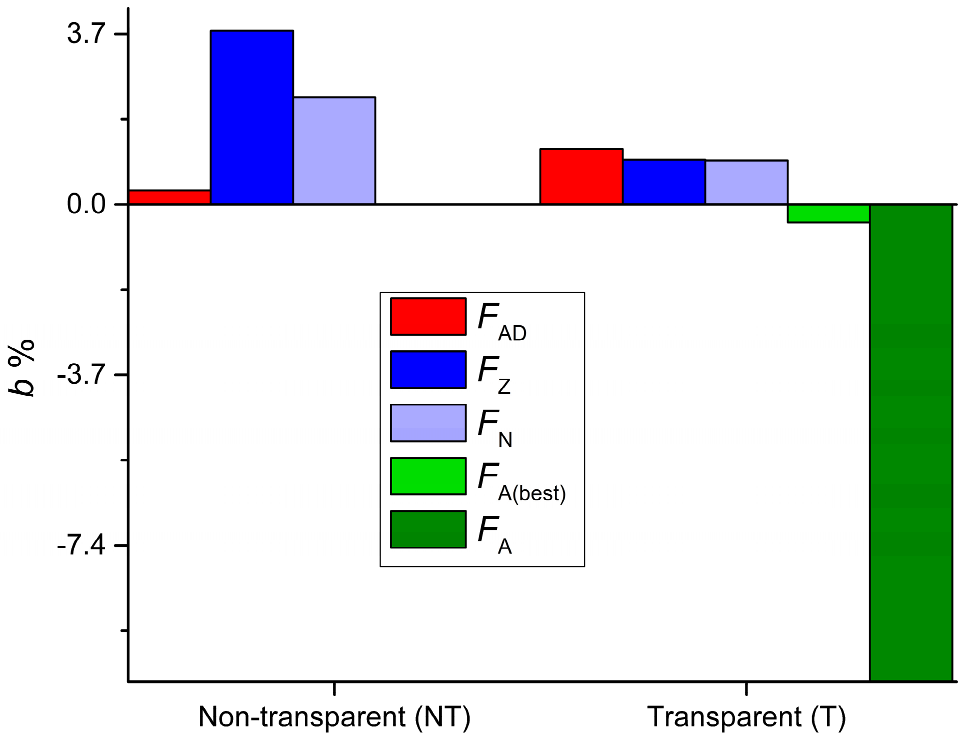

Figure S24. Overview of *b* % values obtained for the different IFE corrections for low fluorophore concentrations (L_x titrations), corresponding to the results in Table 1 (T microplates) and Table S4, SI (NT microplates). The values of F_{AD}, F_Z, F_N, $F_{A(best)}$, and F_A correspond to: AddAbs correction, ZINFE correction, NINFE correction, best Lakowicz correction from the whole set of *z*-positions, and Lakowicz correction obtained at the same *z*-position as F_{AD}, respectively. *b* % values closer to zero indicate less deviation from the ideal physical model.

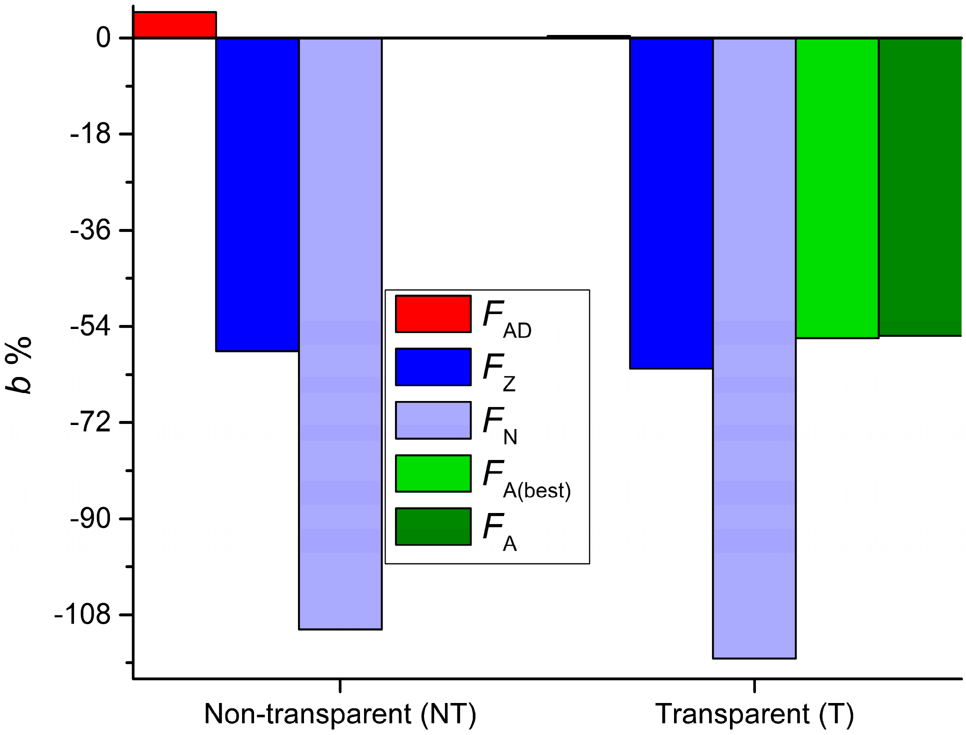

Figure S25. Overview of *b* % values obtained for the different IFE corrections for high fluorophore concentrations (H_x titrations), corresponding to the results in Table 1 (T microplates) and Table S4, SI (NT microplates). The values of F_{AD}, F_Z, F_N, $F_{A(best)}$, and F_A correspond to: AddAbs correction, ZINFE correction, NINFE correction, best Lakowicz correction from the whole set of *z*-positions, and Lakowicz correction obtained at the same *z*-position as F_{AD}, respectively. *b* % values closer to zero indicate less deviation from the ideal physical model.

5.4.3 Percent error of the Limit Of Detection for normalized data, LOD %

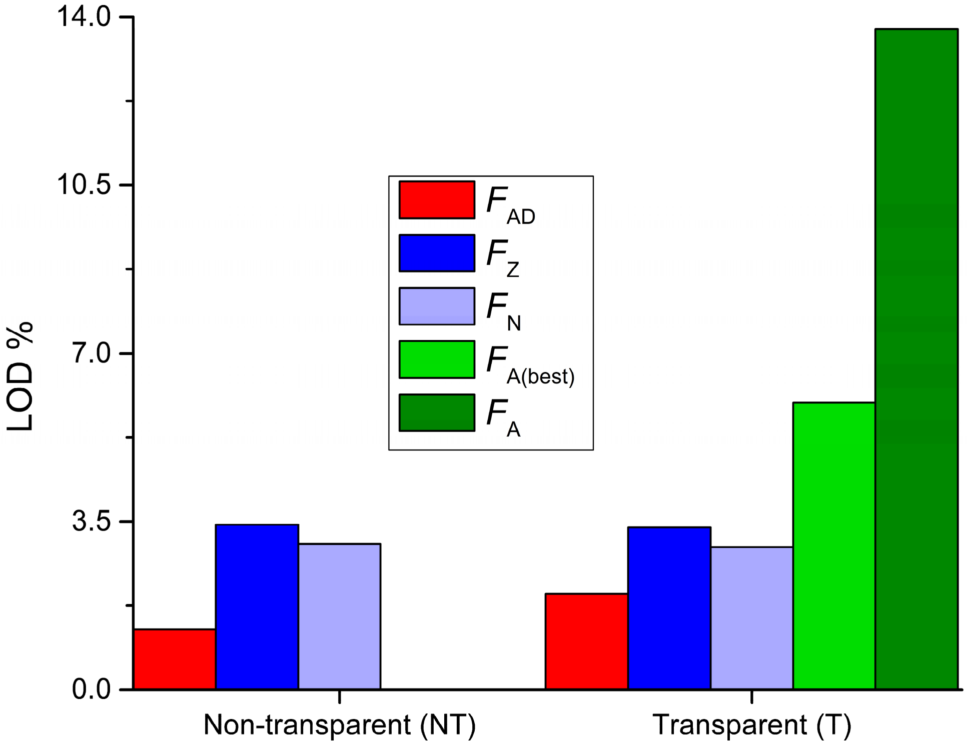

Figure S26. Overview of LOD % values obtained for the different IFE corrections for low fluorophore concentrations (L_x titrations), corresponding to the results in Table 1 (T microplates) and Table S4, SI (NT microplates). The values of F_{AD}, F_Z, F_N, $F_{A(best)}$, and F_A correspond to: AddAbs correction, ZINFE correction, NINFE correction, best Lakowicz correction from the whole set of *z*-positions, and Lakowicz correction obtained at the same *z*-position as F_{AD}, respectively. Lower LOD % values indicate a better detection limit of the correction method.

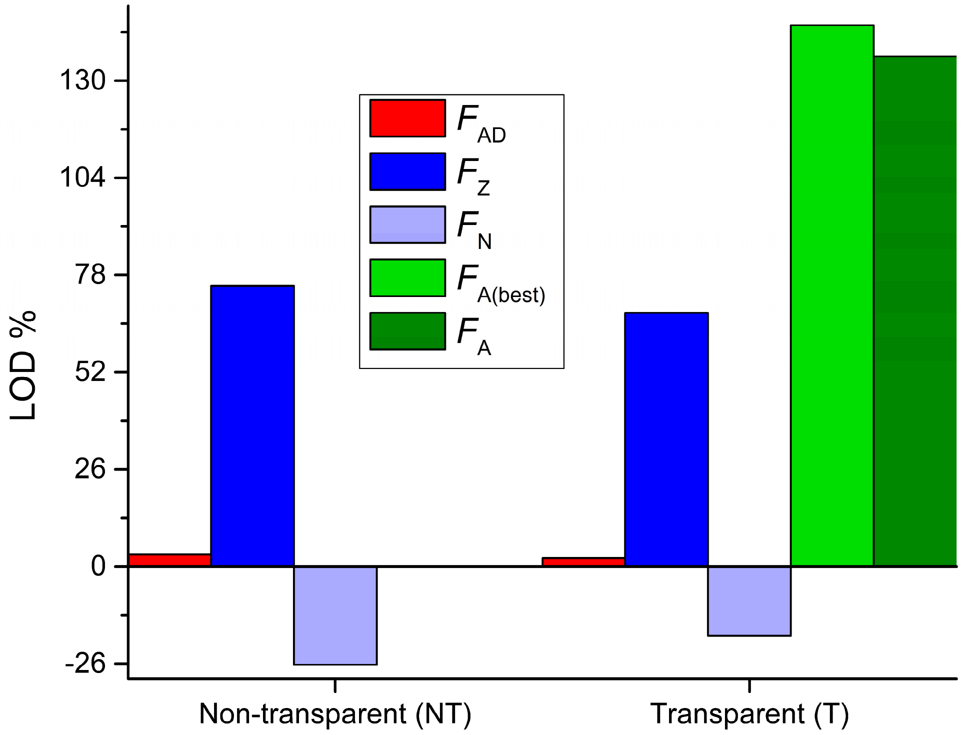

Figure S27. Overview of LOD % values obtained for the different IFE corrections for high fluorophore concentrations (H_x titrations), corresponding to the results in Table 1 (T microplates) and Table S4, SI (NT microplates). The values of F_{AD}, F_Z, F_N, $F_{A(best)}$, and F_A correspond to: AddAbs correction, ZINFE correction, NINFE correction, best Lakowicz correction from the whole set of *z*-positions, and Lakowicz correction obtained at the same *z*-position as F_{AD}, respectively. Lower LOD % values indicate a better detection limit of the correction method. The ZINFE corrections for the LOD % are those with a positive slope constraint. When optimizing the exponent for the H_1 set (NINFE calculation from the ZINFE output data), the positive slope becomes negative, which in turn leads to meaningless (negative) LOD % values.

5.5 AddAbs IFE correction - overview of results for all *z*-positions

Table S5. Results of the AddAbs correction method for all titrations performed at each measured *z*-position. Uncorrected data (L_1 titration) are shown for comparison.

	***z* / mm**	**14.6**			**15**		
**Titration**	**Plate type**	R^2	***b* %**	**LOD %**	R^2	***b* %**	**LOD %**
L_1	T	0.78470	40.684	55.509	0.76611	42.022	58.554
	NT	0.75298	43.544	60.697	0.74084	44.050	62.679
L_2	T	0.92578	25.439	0.962	0.91835	26.391	0.962
	NT	0.90601	27.131	0.971	0.90689	27.499	0.968
L_3	T	0.96231	17.355	20.973	0.96165	17.711	21.161
	NT	0.97063	17.447	18.433	0.96771	17.794	19.359
L_4	T	0.99042	8.621	10.422	0.98953	8.609	10.901
	NT	0.99143	8.761	9.852	0.99142	8.907	9.860
L_5	T	0.99747	4.430	5.338	0.99686	4.690	5.949
	NT	0.99801	3.465	4.738	0.99828	3.396	4.404
L_6	T	0.99887	2.968	3.569	0.99893	2.923	3.468
	NT	0.99816	3.254	4.549	0.99859	2.754	3.978
L_7	T	0.99932	1.824	2.760	0.99951	1.514	2.337
	NT	0.99904	1.982	3.285	0.99874	1.566	3.757
L_8	T	0.99923	1.525	2.942	0.99930	1.458	2.812
	NT	0.99967	0.559	1.918	0.99981	0.465	1.456
L_9	T	0.99706	0.055	5.758	0.99332	0.016	8.689
	NT	0.99618	−1.915	6.559	0.99839	−1.050	4.252
L_{10}	T	0.99610	−1.006	6.630	0.99580	−4.198	6.879
	NT	0.99324	−11.849	8.742	0.99465	−8.247	7.773
L_{11}	T	0.77604	4.563	56.929	0.81227	0.585	50.946
	NT	0.47453	−72.302	111.516	0.89180	−68.375	36.913
L_{12}	T	0.00782	68.750	1,193.680	0.07982	165.405	−359.821
	NT	0.92420	17.143	30.349	0.89726	23.399	35.859
H_1	T	0.71892	151.524	−66.263	0.71201	155.561	−67.396
	NT	0.72260	133.733	−65.659	0.71558	136.732	−66.812
H_2	T	0.99350	6.221	8.571	0.99371	6.215	8.430
	NT	0.99313	5.740	8.815	0.99448	6.027	7.896
H_3	T	0.99836	−0.881	4.291	0.99917	−2.416	3.051
	NT	0.99863	−1.655	3.919	0.99836	−3.403	4.289

(Table S5. continued)

	z / mm	15.5			16		
**Titration**	**Plate type**	R^2	***b* %**	**LOD %**	R^2	***b* %**	**LOD %**
L_1	T	0.76994	41.952	57.928	0.77735	41.266	56.716
	NT	0.74645	43.953	61.763	0.76920	42.064	58.049
L_2	T	0.91418	26.609	0.960	0.91718	26.335	0.959
	NT	0.90312	27.771	0.969	0.90791	26.996	0.966
L_3	T	0.95995	18.226	21.646	0.95914	18.203	21.872
	NT	0.96865	17.971	19.065	0.96645	18.023	19.746
L_4	T	0.98810	9.024	11.632	0.98643	9.402	12.427
	NT	0.99091	9.144	10.150	0.99016	9.277	10.564
L_5	T	0.99672	4.781	6.075	0.99667	4.865	6.126
	NT	0.99812	3.441	4.604	0.99824	3.280	4.444
L_6	T	0.99920	2.633	3.003	0.99926	2.585	2.885
	NT	0.99810	2.851	4.628	0.99849	2.784	4.117
L_7	T	0.99940	1.452	2.599	0.99917	1.243	3.055
	NT	0.99848	1.258	4.137	0.99806	1.277	4.667
L_8	T	0.99938	1.601	2.647	0.99950	1.648	2.375
	NT	0.99986	0.301	1.248	0.99983	−0.179	1.383
L_9	T	0.99246	0.824	9.234	0.99720	1.924	5.616
	NT	0.99927	−1.274	2.871	0.99937	−1.927	2.661
L_{10}	T	0.97779	−3.869	15.972	0.99657	−1.758	6.213
	NT	0.99450	−5.830	7.883	0.99667	−3.066	6.123
L_{11}	T	0.74958	−27.138	61.252	0.94647	−5.886	25.202
	NT	0.96013	−74.019	21.595	0.95883	−29.603	21.958
L_{12}	T	0.06951	−204.762	387.742	0.25021	50.678	183.449
	NT	0.08008	62.740	359.182	0.65847	41.183	76.320
H_1	T	0.69579	159.839	−70.072	0.68768	161.336	−71.418
	NT	0.70038	139.671	−69.313	0.70459	141.951	−68.618
H_2	T	0.99351	6.415	8.563	0.99250	6.325	9.210
	NT	0.99377	6.416	8.391	0.99355	6.306	8.540
H_3	T	0.99835	−1.458	4.311	0.99862	−1.701	3.933
	NT	0.99772	−1.399	5.063	0.99601	−0.525	6.707

(Table S5. continued)

	z / mm	**17**			**18**		
**Titration**	**Plate type**	R^2	***b* %**	**LOD %**	R^2	***b* %**	**LOD %**
L_1	T	0.85734	33.926	43.228	0.92231	25.824	30.757
	NT	0.86645	33.795	41.606	0.92819	25.977	29.476
L_2	T	0.93656	22.546	0.966	0.96455	17.482	0.980
	NT	0.93627	22.289	0.975	0.96451	17.322	0.983
L_3	T	0.96582	16.414	19.937	0.98046	12.637	14.962
	NT	0.97486	15.634	17.018	0.98298	12.599	13.944
L_4	T	0.98538	9.251	12.909	0.99389	7.550	8.310
	NT	0.99192	8.771	9.567	0.99246	7.885	9.239
L_5	T	0.99574	5.480	6.933	0.99808	4.117	4.654
	NT	0.99813	3.155	4.581	0.99820	3.537	4.506
L_6	T	0.99923	2.251	2.950	0.99911	2.749	3.155
	NT	0.99834	2.670	4.323	0.99842	2.955	4.218
L_7	T	0.99923	1.340	2.932	0.99935	2.036	2.709
	NT	0.99738	0.773	5.430	0.99879	2.039	3.690
L_8	T	0.99932	1.292	2.771	0.99964	1.544	2.016
	NT	0.99961	0.192	2.081	0.99972	0.552	1.783
L_9	T	0.99202	2.512	9.507	0.99445	3.238	7.916
	NT	0.99901	−2.270	3.341	0.99931	−0.284	2.776
L_{10}	T	0.99470	0.149	7.738	0.99397	−1.380	8.257
	NT	0.99491	−4.510	7.577	0.99708	−1.452	5.739
L_{11}	T	0.89537	13.804	36.226	0.97865	13.006	15.654
	NT	0.97576	−16.029	16.704	0.96168	3.134	21.153
L_{12}	T	0.42548	−48.376	123.142	0.19783	21.585	213.394
	NT	0.69759	−91.012	69.774	0.58314	−5.149	89.599
H_1	T	0.75322	151.271	−60.658	0.94031	123.963	−26.699
	NT	0.77797	137.779	−56.613	0.92548	113.616	−30.071
H_2	T	0.99223	6.239	9.376	0.99399	6.821	8.239
	NT	0.99343	6.656	8.615	0.99436	7.457	7.984
H_3	T	0.99725	−2.563	5.566	0.99952	0.365	2.329
	NT	0.99699	1.720	5.820	0.99909	4.826	3.194

(Table S5. continued)

	z / mm	19			20		
**Titration**	**Plate type**	R^2	***b* %**	**LOD %**	R^2	***b* %**	**LOD %**
L_1	T	0.94631	21.266	25.241	0.95610	19.611	22.708
	NT	0.95204	21.625	23.786	0.95961	20.056	21.740
L_2	T	0.97467	14.531	0.988	0.97930	13.318	0.990
	NT	0.97537	14.465	0.986	0.97872	13.381	0.987
L_3	T	0.98778	10.269	11.789	0.98994	9.427	10.685
	NT	0.98644	11.144	12.427	0.98710	10.727	12.117
L_4	T	0.99696	5.908	5.849	0.99745	5.132	5.357
	NT	0.99271	7.133	9.082	0.99314	6.646	8.809
L_5	T	0.99847	2.818	4.150	0.99769	1.949	5.095
	NT	0.99794	4.020	4.811	0.99735	4.535	5.468
L_6	T	0.99720	3.311	5.611	0.99567	4.889	6.991
	NT	0.99860	3.213	3.969	0.99808	3.473	4.644
L_7	T	0.99922	2.409	2.963	0.99945	2.219	2.489
	NT	0.99806	3.218	4.673	0.99532	3.893	7.263
L_8	T	0.99964	1.199	2.001	0.99949	1.918	2.391
	NT	0.99847	2.071	4.144	0.99837	2.597	4.281
L_9	T	0.99813	2.093	4.588	0.99646	1.952	6.313
	NT	0.99892	0.457	3.482	0.99548	3.153	7.145
L_{10}	T	0.99545	2.685	7.166	0.98693	3.268	12.195
	NT	0.99833	0.469	4.334	0.99013	7.187	10.583
L_{11}	T	0.84990	1.226	44.536	0.82055	23.264	49.558
	NT	0.88489	−1.902	38.222	0.69453	34.653	70.281
L_{12}	T	0.14437	−37.639	257.985	0.12923	20.883	275.087
	NT	0.07567	77.631	370.389	0.81926	−10.876	49.776
H_1	T	0.00026	100.152	−6519.173	0.33948	90.705	147.820
	NT	0.06012	97.747	419.025	0.35592	91.230	142.558
H_2	T	0.99393	8.048	8.279	0.99295	8.894	8.931
	NT	0.99538	8.366	7.222	0.99587	7.405	6.826
H_3	T	0.99653	4.444	6.249	0.98589	7.784	12.679
	NT	0.99850	7.496	4.114	0.99482	−1.985	7.650

(Table S5. continued)

	z / mm	21			34.217		
**Titration**	**Plate type**	R^2	b %	LOD %	R^2	b %	LOD %
L_1	T	0.95808	19.377	22.168	0.92580	25.960	30.002
	NT	0.95846	20.182	22.062	0.92328	26.852	30.549
L_2	T	0.97946	13.239	0.990	0.97034	16.186	0.987
	NT	0.98057	13.100	0.987	0.96588	17.453	0.982
L_3	T	0.99035	9.340	10.462	0.98664	9.095	12.330
	NT	0.98736	10.659	11.992	0.98240	12.553	14.185
L_4	T	0.99781	5.053	4.961	0.99602	5.682	6.699
	NT	0.99335	6.487	8.668	0.99501	6.546	7.501
L_5	T	0.99773	1.949	5.054	0.99434	0.250	7.992
	NT	0.99694	4.650	5.874	0.99849	1.863	4.123
L_6	T	0.99550	4.096	7.124	0.97399	8.707	17.317
	NT	0.99813	3.209	4.591	0.99801	−0.530	4.735
L_7	T	0.99933	2.757	2.751	0.73171	−62.662	64.170
	NT	0.99449	3.661	7.886	0.98821	−3.417	11.575
L_8	T	0.99960	1.590	2.112	0.73386	−1.839	63.818
	NT	0.99844	2.037	4.183	0.81538	−0.932	50.426
L_9	T	0.99178	0.084	9.650	0.60156	44.097	86.246
	NT	0.99645	3.310	6.323	0.09960	55.133	318.623
L_{10}	T	0.97358	3.136	17.457	0.03033	46.641	599.187
	NT	0.98492	8.461	13.112	0.06865	199.698	−390.326
L_{11}	T	0.56618	18.383	92.762	0.37005	53.216	138.268
	NT	0.69277	9.285	70.571	0.45553	−711.257	115.858
L_{12}	T	0.36449	−6.613	139.930	0.66489	11.374	75.233
	NT	0.02561	129.675	−653.635	0.59354	−1.588	87.697
H_1	T	0.42349	88.606	123.645	0.22959	102.586	−194.125
	NT	0.39889	90.086	130.091	0.01088	99.538	1010.392
H_2	T	0.99380	8.483	8.369	0.99025	11.263	10.513
	NT	0.99507	7.428	7.459	0.99662	3.921	6.167
H_3	T	0.98706	9.524	12.133	0.84862	−3.453	44.759
	NT	0.99572	1.408	6.946	0.94363	−22.187	25.902

5.6 Lakowicz IFE correction - overview of results for all *z*-positions

For the L_5-L_{12} sets, the absorbance values at A_{ex} = 345 nm and A_{em} = 390 nm were too high to be measured directly because the solution contained a significant concentration of the added absorber. As described chapter 4.2, SI, the Lakowicz correction formula can be split into 2 exponential terms, one containing the variable absorbance values (contribution of the fluorophore) and the second containing the baseline absorbance values (from the added absorber). Since the baseline term does not affect the overall correction function and the theoretical quantities of QS are the same for all L_1-L_{12} titrations, we decided to use baseline-corrected absorbance values obtained for the L_1 set for the corrections of the L_5-L_{12} sets.

For the H_1 set (containing only the concentrated solution QS), the absorbance values were measured directly at A_{em} = 390 nm and the absorbance values at A_{em} = 345 nm were calculated using the ratio $A_{345\text{ nm}}$ / $A_{390\text{ nm}}$ = 15.800. This ratio was determined experimentally from the averaged baseline-corrected absorbance measurements of the titration point c_{norm} = 0.1 ($A_{345\text{ nm}}$ = 1.8511 and $A_{390\text{ nm}}$ = 0.11657. For titrations H_2 and H_3, the same estimated baseline-corrected absorbance values were used for the Lakowicz correction as for H_1.

Table S6. Results of Lakowicz correction for all titrations (T plate) performed at each measured *z*-position. The corrections were performed for the L_1-L_4 titrations using the baseline corrected absorbance values at A_{ex} = 345 nm and A_{em} = 390 nm. For the other sets, the baseline-corrected absorbance values were estimated as described above.

z / mm	14.6			15		
**Titration**	R^2	***b* %**	**LOD %**	R^2	***b* %**	**LOD %**
L_1	0.99558	−1.002	7.061	0.99594	−0.336	6.770
L_2	0.98606	−8.789	12.601	0.98750	−8.300	11.922
L_3	0.97818	−11.377	15.826	0.97850	−11.210	15.709
L_4	0.98317	−12.039	13.863	0.98374	−12.018	13.625
L_5	0.95250	−18.953	23.664	0.95285	−18.810	23.575
L_6	0.95036	−19.689	24.220	0.94987	−19.723	24.346
L_7	0.94667	−20.344	25.154	0.94777	−20.436	24.877
L_8	0.94845	−20.378	24.706	0.94785	−20.430	24.858
L_9	0.94831	−21.024	24.742	0.95178	−20.857	23.854
L_{10}	0.93200	−21.968	28.625	0.93064	−23.294	28.930
L_{11}	0.96367	−15.382	20.575	0.94027	−19.147	26.710
L_{12}	0.09681	10.821	323.691	0.13834	41.642	264.473
H_1	0.36161	−55.948	140.806	0.36266	−55.928	140.487
H_2	0.35485	−56.115	142.891	0.35404	−56.129	143.143
H_3	0.34935	−56.220	144.623	0.34760	−56.254	145.182

(Table S6. continued)

z / mm	15.5			16		
**Titration**	R^2	*b* %	LOD %	R^2	*b* %	LOD %
L_1	0.99683	−0.394	5.979	0.99662	−0.716	6.174
L_2	0.98831	−8.151	11.526	0.98798	−8.293	11.691
L_3	0.97857	−10.965	15.684	0.97880	−10.960	15.595
L_4	0.98482	−11.753	13.156	0.98595	−11.498	12.649
L_5	0.95213	−18.780	23.761	0.95294	−18.735	23.550
L_6	0.94805	−19.910	24.806	0.94793	−19.940	24.837
L_7	0.94719	−20.487	25.022	0.94824	−20.526	24.759
L_8	0.94823	−20.379	24.762	0.94522	−20.438	25.511
L_9	0.95843	−20.329	22.071	0.95174	−20.117	23.864
L_{10}	0.92291	−23.252	30.628	0.93284	−22.257	28.434
L_{11}	0.85730	−32.168	43.236	0.95810	−22.610	22.160
L_{12}	0.05668	−129.552	432.312	0.84493	2.549	45.399
H_1	0.36253	−55.930	140.527	0.36293	−55.924	140.404
H_2	0.35438	−56.124	143.036	0.35444	−56.121	143.019
H_3	0.34763	−56.250	145.172	0.34780	−56.246	145.118

(Table S6. continued)

z / mm	17			18		
**Titration**	R^2	*b* %	LOD %	R^2	*b* %	LOD %
L_1	0.99441	−4.211	7.949	0.98782	−8.156	11.766
L_2	0.98468	−10.075	13.220	0.97710	−12.652	16.225
L_3	0.97690	−11.859	16.297	0.97065	−13.863	18.426
L_4	0.98667	−11.540	12.319	0.98012	−12.729	15.094
L_5	0.95214	−18.468	23.758	0.95290	−19.095	23.562
L_6	0.94624	−20.137	25.258	0.94978	−19.806	24.367
L_7	0.94654	−20.559	25.186	0.94827	−20.196	24.753
L_8	0.94542	−20.556	25.463	0.94679	−20.429	25.123
L_9	0.95859	−19.596	22.026	0.95581	−19.396	22.786
L_{10}	0.93911	−21.193	26.985	0.93514	−21.973	27.908
L_{11}	0.90136	−15.975	35.057	0.92736	−16.243	29.658
L_{12}	0.50757	−35.045	104.381	0.40120	1.551	129.465
H_1	0.36273	−55.929	140.463	0.36191	−55.944	140.714
H_2	0.35482	−56.116	142.900	0.35339	−56.141	143.349
H_3	0.34554	−56.292	145.844	0.34877	−56.231	144.809

(Table S6. continued)

z / mm	19			20		
**Titration**	R^2	*b* %	**LOD %**	R^2	*b* %	**LOD %**
L_1	0.98346	−10.352	13.742	0.98029	−11.219	15.026
L_2	0.97297	−14.101	17.664	0.97004	−14.744	18.625
L_3	0.96528	−15.155	20.099	0.96316	−15.616	20.724
L_4	0.97398	−13.797	17.320	0.97015	−14.322	18.589
L_5	0.95260	−19.701	23.640	0.95171	−20.105	23.871
L_6	0.95312	−19.409	23.503	0.95482	−18.654	23.051
L_7	0.94922	−19.995	24.510	0.94562	−20.169	25.413
L_8	0.94625	−20.602	25.258	0.94551	−20.318	25.441
L_9	0.94695	−20.161	25.082	0.93666	−20.521	27.557
L_{10}	0.94100	−20.091	26.535	0.95329	−19.307	23.459
L_{11}	0.93612	−19.640	27.683	0.92310	−11.363	30.586
L_{12}	0.20646	−28.253	207.761	0.28988	−6.690	165.866
H_1	0.36056	−55.964	141.126	0.36003	−55.973	141.288
H_2	0.35263	−56.151	143.587	0.35191	−56.163	143.814
H_3	0.35420	−56.128	143.095	0.35601	−56.092	142.528

(Table S6. continued)

z / mm	21			34.217		
**Titration**	R^2	*b* %	LOD %	R^2	*b* %	LOD %
L_1	0.97846	−11.393	15.722	0.98642	−8.172	12.436
L_2	0.96999	−14.782	18.640	0.97416	−13.334	17.259
L_3	0.96280	−15.672	20.831	0.96677	−15.635	19.647
L_4	0.97022	−14.371	18.566	0.97571	−13.838	16.719
L_5	0.95271	−20.070	23.611	0.95388	−20.726	23.301
L_6	0.95537	−18.976	22.904	0.96219	−16.528	21.008
L_7	0.94695	−19.891	25.084	0.92456	−45.616	30.271
L_8	0.94591	−20.443	25.342	0.84088	−22.532	46.098
L_9	0.92418	−21.609	30.353	0.91000	−2.815	33.328
L_{10}	0.94355	−19.521	25.921	0.08162	16.285	355.466
L_{11}	0.82492	−15.031	48.821	0.79480	−0.642	53.847
L_{12}	0.50696	−25.883	104.509	0.91674	−14.305	31.938
H_1	0.35955	−55.980	141.434	0.35979	−55.977	141.362
H_2	0.35122	−56.177	144.031	0.35102	−56.177	144.094
H_3	0.35627	−56.085	142.449	0.37597	−55.765	136.528

5.7 ZINFE/NINFE correction - overview of best results

Table S7. Best obtained ZINFE and NINFE correction results considering R^2 criterion (R^2 closest to one). Numerical optimization problems occurred for sets L_{10} - L_{12} (described in the manuscript), so these results are not shown.

Sample	Plate type	Correction type	z_1	z_2	R^2	*b* %	LOD %
L_1	T	ZINFE	18	16	0.99899	0.969	3.374
		NINFE	18	15.5	0.99922	−0.949	2.965
	NT	ZINFE	18	14.6	0.99918	−3.761	3.433
		NINFE	18	14.6	0.99918	−2.324	3.034
L_2	T	ZINFE	18	16	0.99885	−1.530	3.598
		NINFE	18	15	0.99930	0.254	2.798
	NT	ZINFE	18	15.5	0.99881	−0.860	3.664
		NINFE	18	15	0.99894	−0.397	3.445
L_3	T	ZINFE	19	15.5	0.99972	−0.618	1.766
		NINFE	19	15	0.99981	−0.225	1.468
	NT	ZINFE	18	16	0.99886	−2.150	4.542
		NINFE	18	15	0.99946	1.225	2.464
L_4	T	ZINFE	21	19	0.99798	−4.380	4.764
		NINFE	21	20	0.99812	−4.890	4.598
	NT	ZINFE	17	16	0.99578	−6.488	6.919
		NINFE	14.6	16	0.99740	−3.605	5.411
L_5	T	ZINFE	19	21	0.99855	−2.998	4.036
		NINFE	19	34.217	0.99881	−3.847	3.662
	NT	ZINFE	15	21	0.99837	−3.312	4.363
		NINFE	19	21	0.99903	−2.383	3.302
L_6	T	ZINFE	15	16	0.99931	−1.389	2.776
		NINFE	15.5	34.217	0.99974	−1.514	1.703
	NT	ZINFE	14.6	16	0.99885	−1.738	3.610
		NINFE	15	14.6	0.99909	−1.377	3.201

(Table S7. continued)

L_7	T	ZINFE	15	16	0.99960	−1.737	2.124
		NINFE	15	16	0.99960	−1.737	2.124
	NT	ZINFE	14.6	19	0.99944	−2.430	2.499
		NINFE	14.6	17	0.99962	−3.255	2.066
L_8	T	ZINFE	18	20	0.99966	−1.654	1.954
		NINFE	18	14.6	0.99973	−1.562	1.733
	NT	ZINFE	15.5	20	0.99987	−0.596	1.207
		NINFE	15.5	15	0.99991	−0.367	1.018
L_9	T	ZINFE	21	16	0.96369	−2.736	5.626
		NINFE	18	21	0.99769	−1.929	5.103
	NT	ZINFE	15.5	19	0.99950	0.499	2.371
		NINFE	16	14.6	0.99955	1.925	2.242
H_1	T	ZINFE	18	20	0.94897	−112.711	−24.575
		NINFE	21	14.6	0.99739	−109.295	−5.419
	NT	ZINFE	15	18	0.93026	−116.365	−29.016
		NINFE	21	16	0.99056	−107.481	−10.348
H_2	T	ZINFE	18	16	0.99533	−8.024	7.259
		NINFE	18	16	0.99534	−7.946	7.254
	NT	ZINFE	20	21	0.99614	−7.397	6.600
		NINFE	15	14.6	0.99772	−7.681	5.063
H_3	T	ZINFE	21	14.6	0.98425	−10.873	13.404
		NINFE	19	14.6	0.99411	−7.402	8.155
	NT	ZINFE	16	18	0.99958	−6.846	2.164
		NINFE	18	16	0.99964	−7.779	2.003

5.8 LOOCV results and residual plots

Table S8. LOOCV results from the titrations that give the best R^2 values for both UV-vis-transparent (T) and non-transparent (NT) microplates and for both the lower (L) and higher (H) QS concentration series. The procedure is performed using ordinary least squares, with all but the *n*th point used for interpolation. The expected value $\hat{c}_{norm}$ is calculated from the slope *b* and the intercept *a*, which are obtained from the linear dependence of the normalized concentration c_{norm} and the normalized fluorescence F_{norm}. The values of the intercept *a* are not listed in the table because they are linearly dependent values $a = 1 - b$ in this particular scaling procedure.

c_{norm}	0.1	0.2	0.3	0.4	0.5	0.6	0.7	0.8	0.9	1	Average ± st. dev.	Set
F_{norm}	0.1033	0.2066	0.3093	0.4120	0.5118	0.6147	0.6997	0.8018	0.9017	0.9930	-	L_8, z = 19 mm, T
$\hat{c}_{norm}$	0.1148	0.2106	0.3082	0.4065	0.5053	0.6037	0.7042	0.8025	0.9010	1.0037	-	
b	0.9817	0.9863	0.9883	0.9890	0.9884	0.9873	0.9888	0.9882	0.9877	0.9938	0.9880 ± 0.0030	
F_{norm}	0.1015	0.2012	0.3054	0.3971	0.5020	0.6078	0.6979	0.8029	0.9020	0.9959	-	L_8, z = 15.5 mm, NT
$\hat{c}_{norm}$	0.1034	0.2028	0.3014	0.4025	0.5015	0.6005	0.7013	0.8001	0.8998	1.0022	-	
b	0.9960	0.9963	0.9982	0.9960	0.9970	0.9965	0.9976	0.9961	0.9960	1.0004	0.9970 ± 0.0014	
F_{norm}	0.0978	0.2026	0.3035	0.4042	0.5014	0.6075	0.7099	0.8011	0.8850	1.0035	-	H_3, z = 18 mm, T
$\hat{c}_{norm}$	0.1062	0.2030	0.3024	0.4019	0.5019	0.6008	0.6998	0.8007	0.9054	0.9981	-	
b	0.9918	0.9962	0.9967	0.9968	0.9963	0.9959	0.9945	0.9962	1.0050	0.9934	0.9963 ± 0.0035	
F_{norm}	0.1287	0.2351	0.3414	0.4389	0.5323	0.6247	0.7082	0.8168	0.8953	0.9959	-	H_3, z = 18 mm, NT
$\hat{c}_{norm}$	0.1512	0.2398	0.3322	0.4275	0.5232	0.6187	0.7154	0.8081	0.9080	1.0022	-	
b	0.9395	0.9497	0.9545	0.9538	0.9523	0.9514	0.9531	0.9491	0.9571	0.9552	0.9516 ± 0.0049	

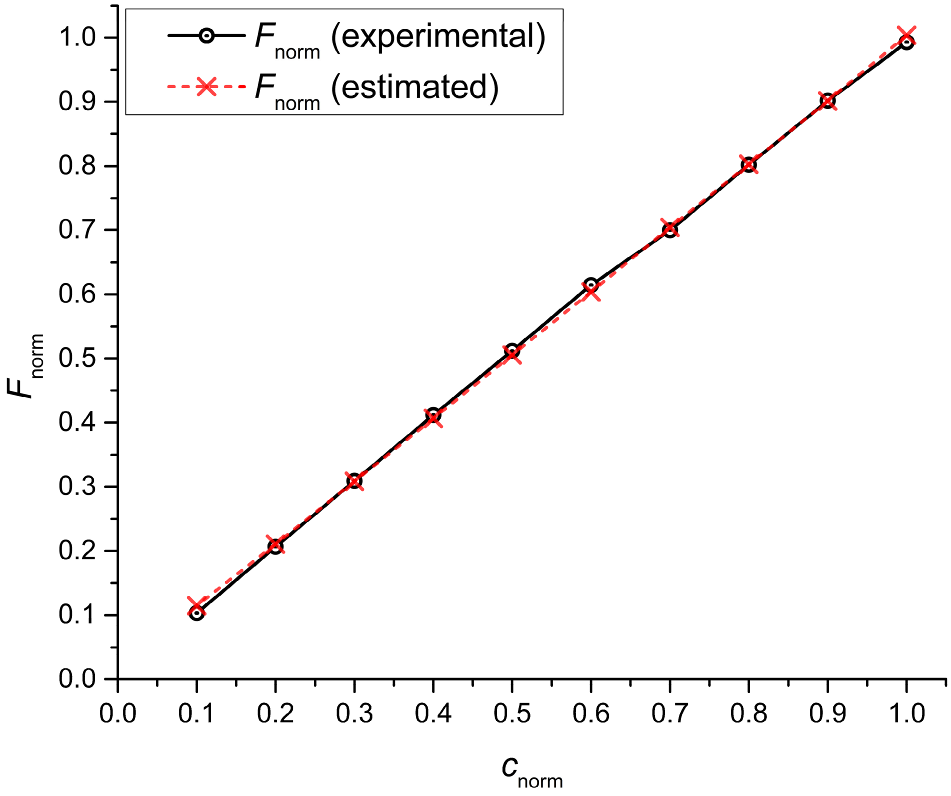

Figure S28. LOOCV results for best AddAbs IFE correction (F_{AD}) in UV-vis-transparent microplate (T), z = 19 mm, L_8 titration.

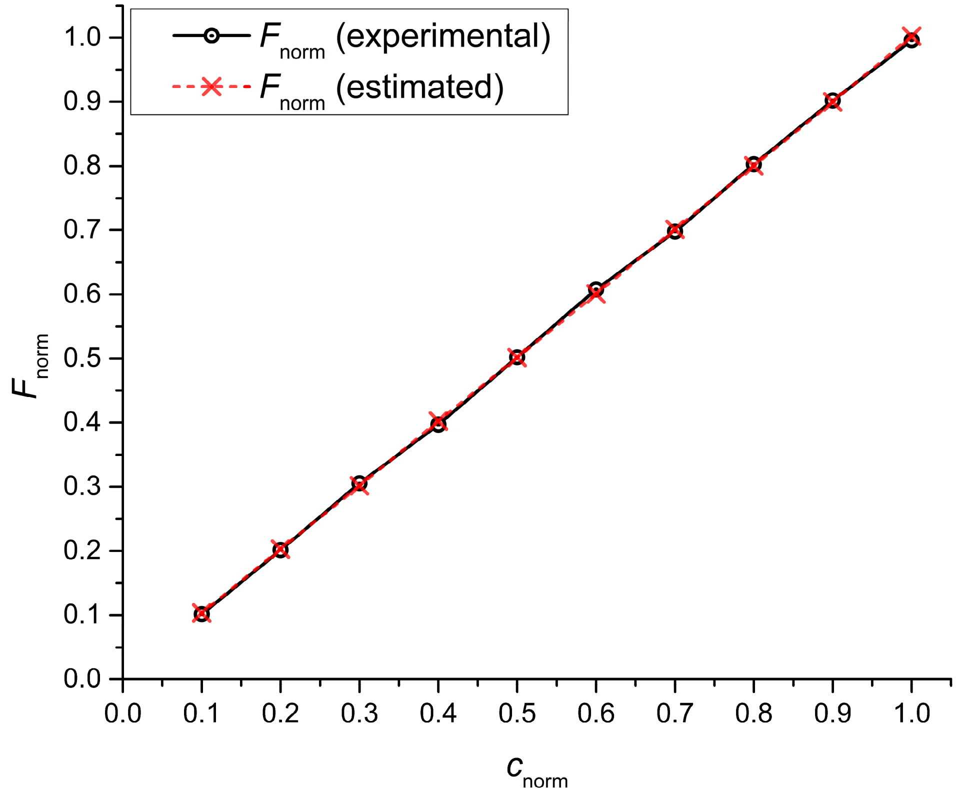

Figure S29. LOOCV results for best AddAbs IFE correction (F_{AD}) in non-transparent microplate (NT), z = 15.5 mm, L_8 titration.

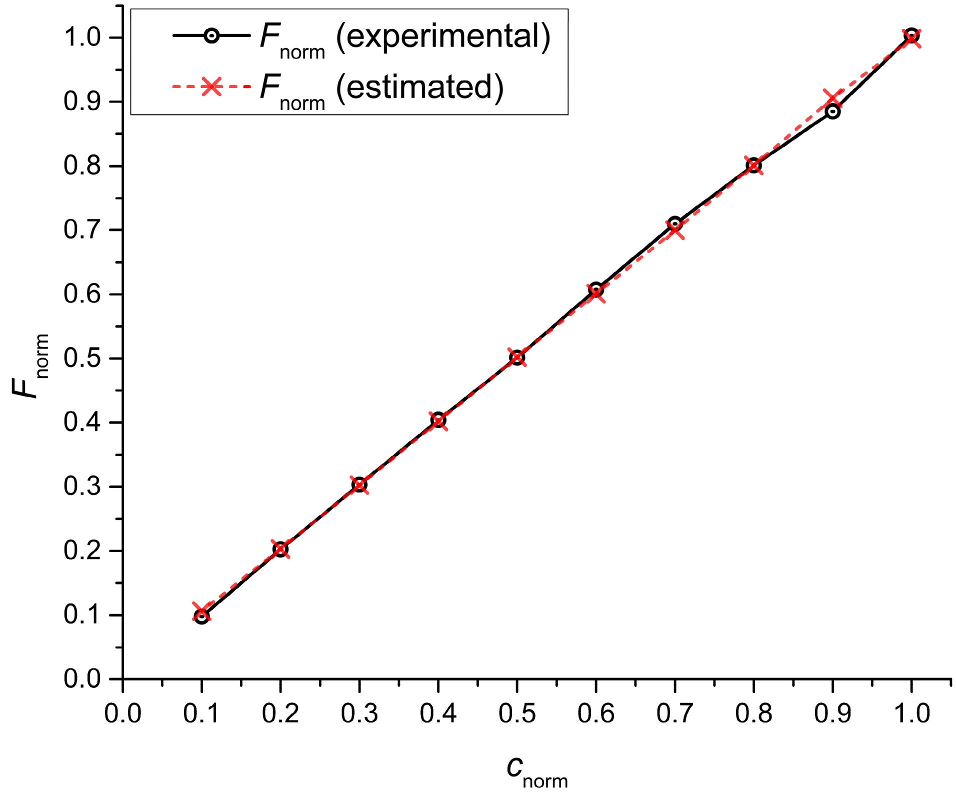

Figure S30. LOOCV results for best AddAbs IFE correction (F_{AD}) in UV-vis-transparent microplate (T), z = 18 mm, H_3 titration.

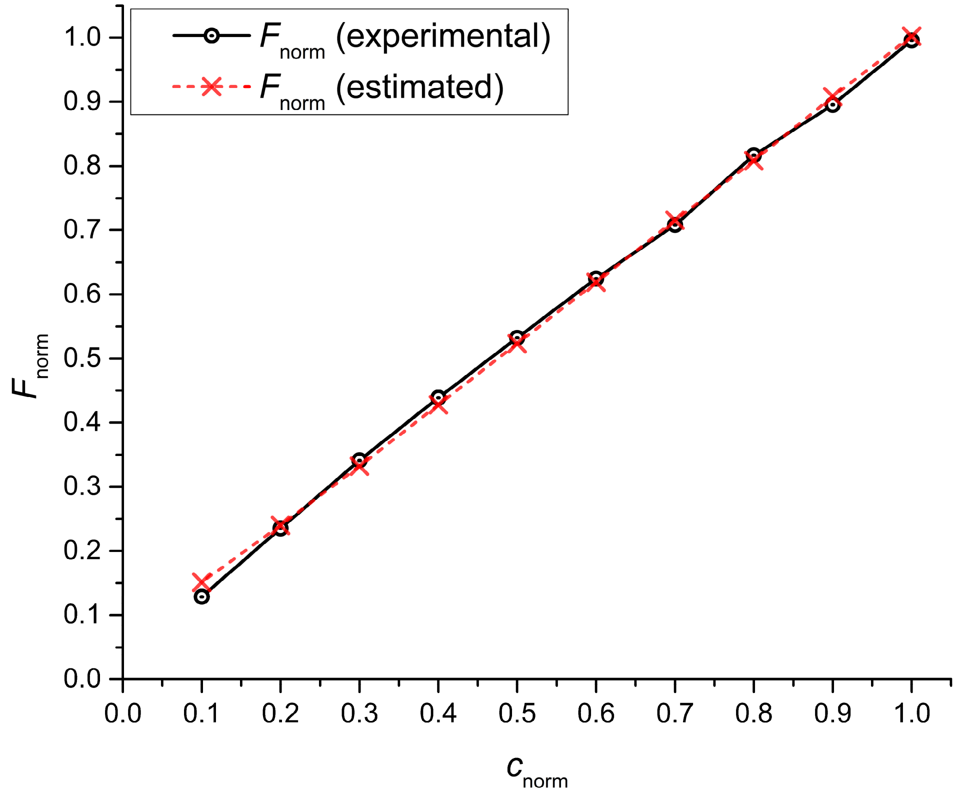

Figure S31. LOOCV results for best AddAbs IFE correction (F_{AD}) in non-transparent microplate (NT), z = 18 mm, H_3 titration.

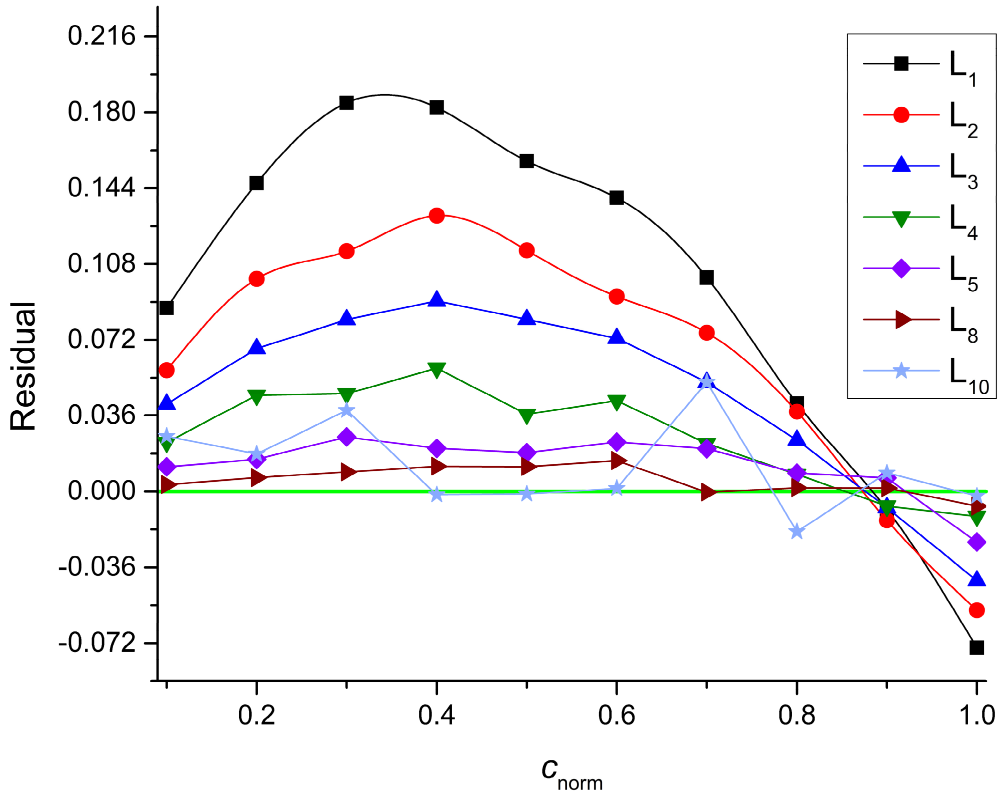

Figure S32. Results of the AddAbs method validation for low fluorophore concentrations in T microplates (z = 19 mm). Residual plots for selected titrations are shown with the ideal residual line (y = 0) highlighted in light green. For visual clarity, results for each titration are not shown.

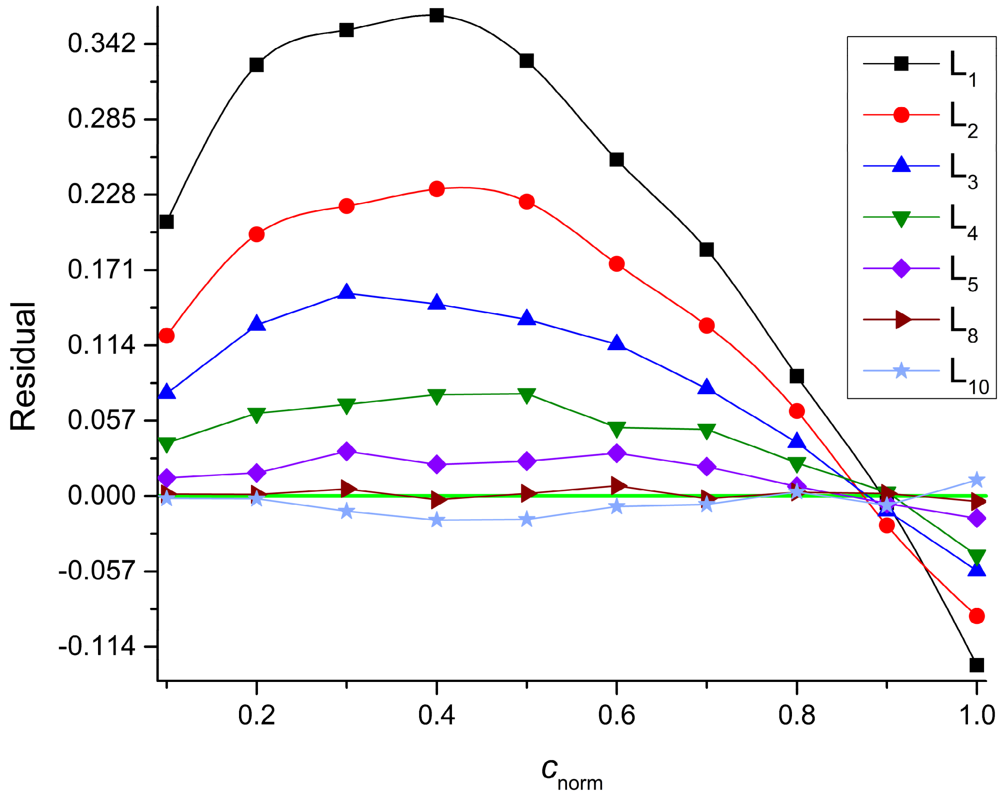

Figure S33. Results of the AddAbs method validation for low fluorophore concentrations in NT microplates (z = 15.5 mm). Residual plots for selected titrations are shown with the ideal residual line (y = 0) highlighted in light green. For visual clarity, results for each titration are not shown.

6 Nonlinear fitting examples

As mentioned previously, for L_1 (uncorrected fluorescence for lower QS concentrations) it might be useful to estimate the concentration of the fluorophore by nonlinear interpolation. The quality of these interpolations depends, among other things, on the z-position. Here we present two examples of the same titration measured at two different z-positions: 14.6 and 18 mm, for the UV-vis-transparent (T) plate. In both cases, the results are obviously worse than those obtained with the AddAbs or ZINFE/NINFE correction methods. In the case of z = 14.6, a clear "plateau" is observed at the last concentration points, while for z = 18 there is still a trend of increasing fluorescence with increasing concentration. Both sets are modeled via a single-exponential fit as $F_{\text{norm}} = a + b \cdot \ln(c_{\text{norm}})$. As can be easily seen from the graphs below, there is a significant deviation from the interpolated curve/line in the case of z = 14.6 mm (Figures S34, S35).

The minimum concentration should give the lowest fluorescence signal unless IFE is extreme. This can be expressed by eq S12.

$$F_{\text{norm,min}} = a + b \cdot \ln\,(c_{\text{norm,min}}) \qquad \text{(S12)}$$

Eq S12 can be transformed to eq S13.

$$c_{\text{norm,min}} = \mathrm{e}^{\frac{F_{\text{norm,min}} - a}{b}} \qquad \text{(S13)}$$

Assuming that the minimum normalized fluorescence signal is much closer to zero than the value of the normalized axis intercept, the following approximation for the minimum normalized concentration is obtained, as shown in eq S14.

$$c_{\text{norm,min}} \approx \mathrm{e}^{\frac{-a}{b}} \qquad \text{(S14)}$$

For the semi-logarithmic interpolation, the Limit Of Detection percent (LOD %) is calculated as described via equation S8 (chapter 4.5, SI).

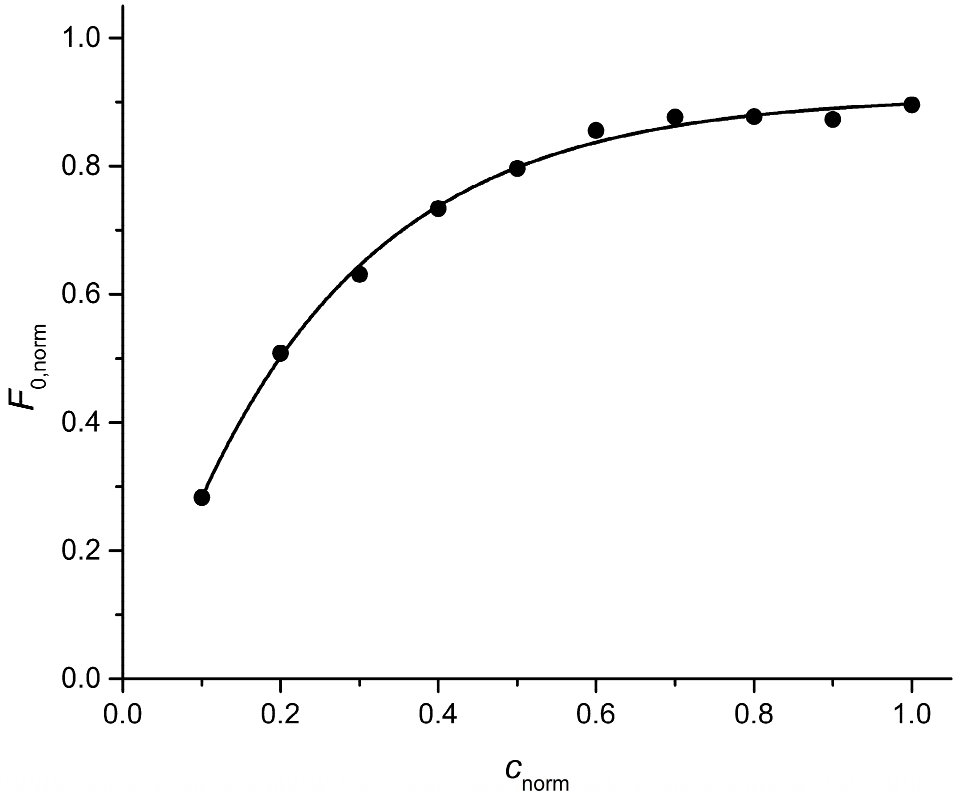

Figure S34. Exponential fit ($F_{norm} = a + b \cdot \ln(c_{norm})$) for IFE-uncorrected fluorescence measured at z = 14.6 mm for L_1 titration in the UV-vis-transparent (T) microplate.

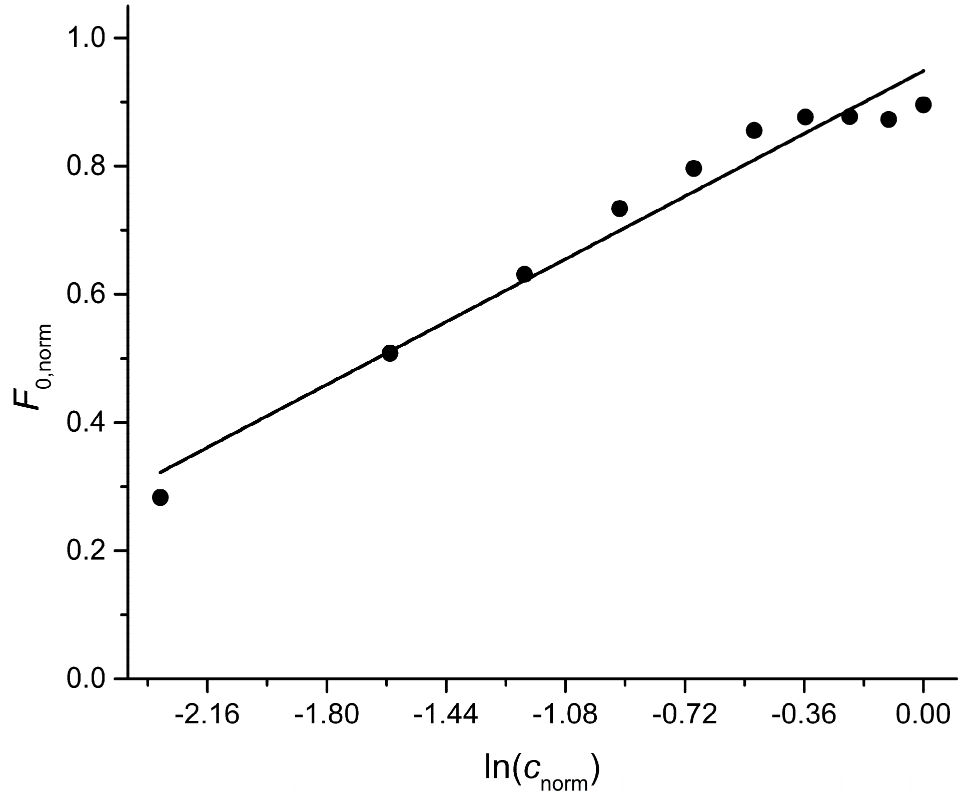

Figure S35. Semi-logarithmic plot of the normalized fluorescence as a function of $\ln(c_{\mathrm{norm}})$ at z = 14.6 mm for L_1 titration in the UV-vis-transparent (T) microplate: $F_{\mathrm{norm}} = a + b \cdot \ln(c_{\mathrm{norm}})$, a = 0.9486, b = 0.2721, R^2 = 0.9677, s_y = 0.0387, $c_{\mathrm{norm,min}}$ = 0.0306.

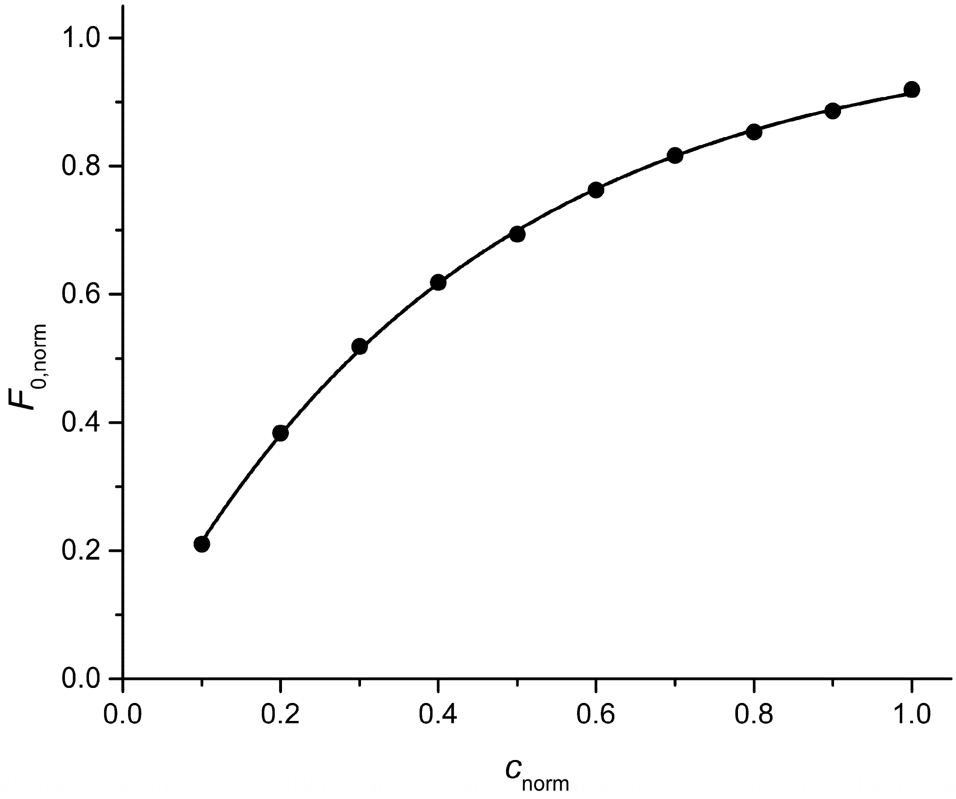

Figure S36. Exponential fit ($F_{norm} = a + b \cdot \ln(c_{norm})$) for IFE-uncorrected fluorescence measured at z = 18 mm for L_1 titration in the UV-vis-transparent (T) plate.

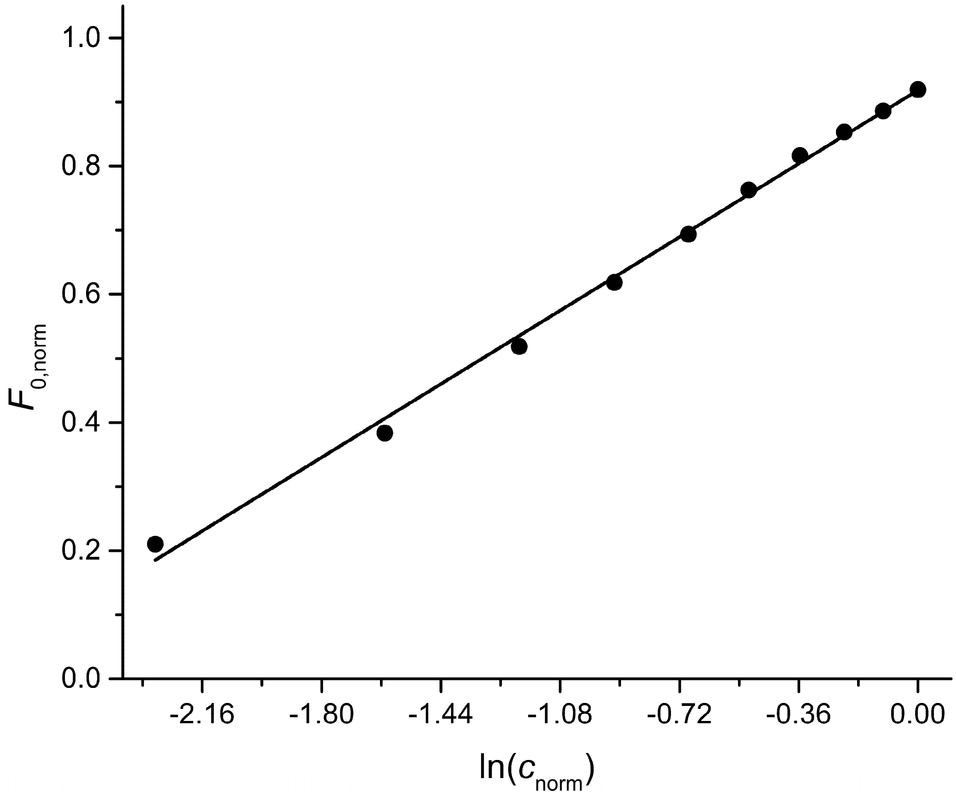

Figure S37. Semi-logarithmic plot of the normalized fluorescence as a function of ln(c_{norm}) at z = 18 mm for L_1 titration in the UV-vis-transparent (T) plate: $F_{norm} = a + b \cdot \ln(c_{norm})$, a = 0.9185, b = 0.3185, R^2 = 0.9966, s_y = 0.0145, $c_{norm,min}$ = 0.0551.

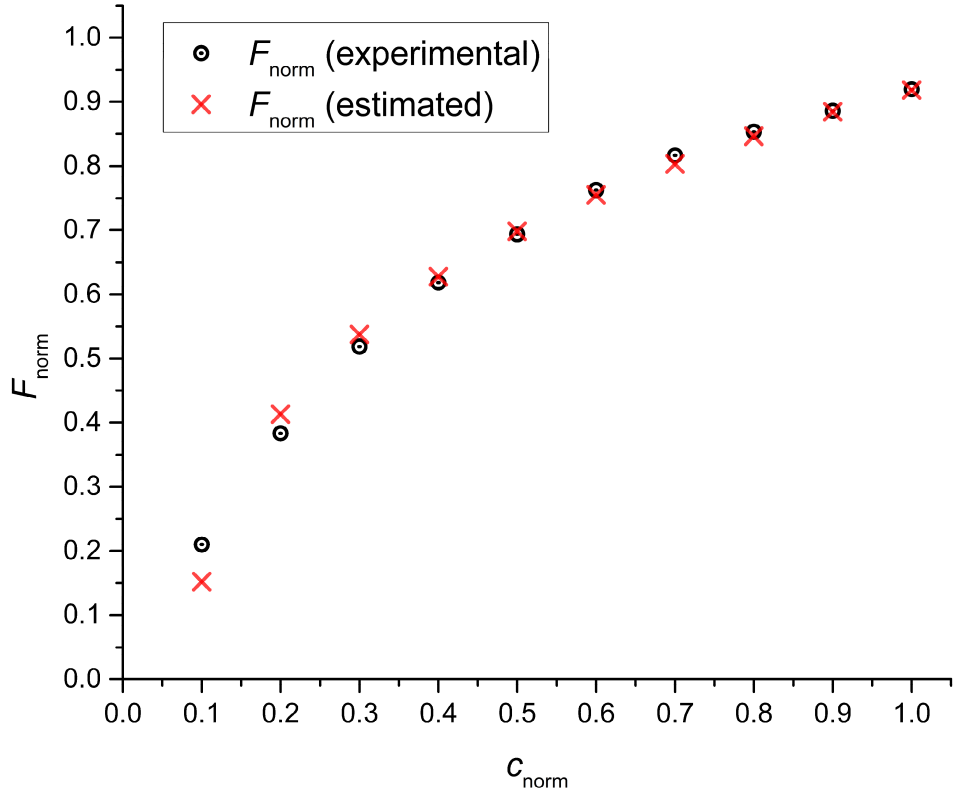

Figure S38. LOOCV results from the exponential fit for IFE-uncorrected fluorescence values measured at z = 18 mm for L_1 titration in the UV-vis-transparent (T) microplate.

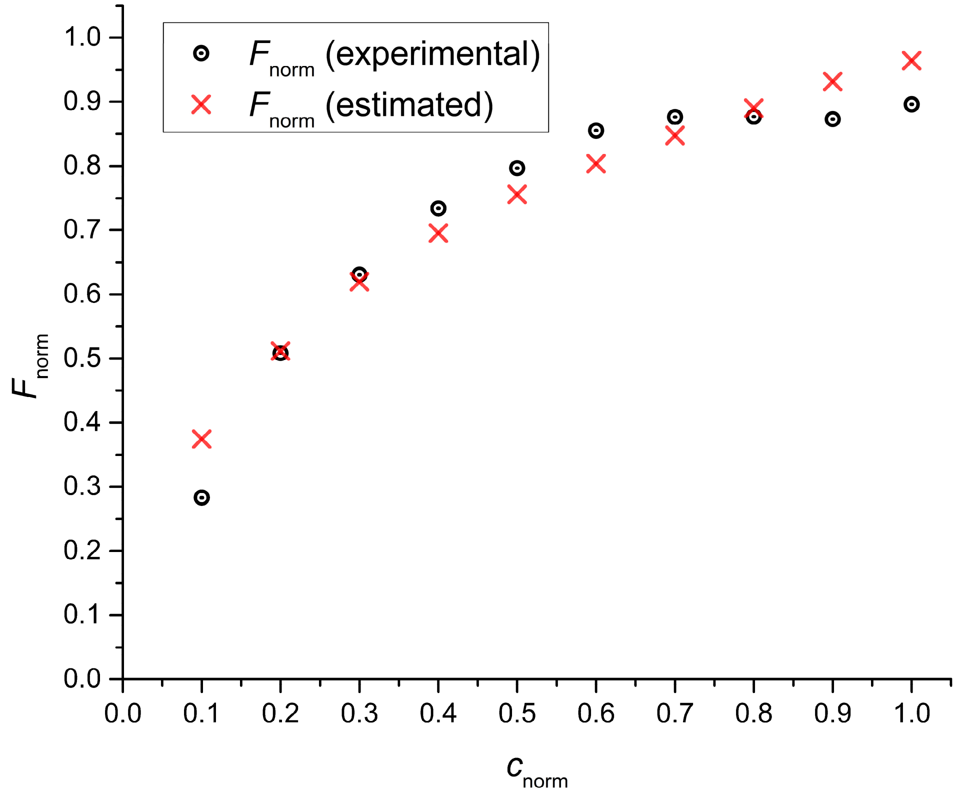

Figure S39. LOOCV results from the exponential fit for IFE-uncorrected fluorescence values measured at z = 14.6 mm for L_1 titration in the UV-vis-transparent (T) microplate.

Table S9. LOOCV results obtained by the exponential fit for IFE -uncorrected fluorescence values (see Table S8 for calculation details).

c_{norm}	**0.1**	**0.2**	**0.3**	**0.4**	**0.5**	**0.6**	**0.7**	**0.8**	**0.9**	**1**	**Average ± st. dev.**	**Set**
F_{norm}	0.3742	0.5115	0.6195	0.6953	0.7558	0.8036	0.8475	0.8900	0.9314	0.9643	-	L_1, z = 18 mm, T
b	0.2436	0.2715	0.2730	0.2731	0.2712	0.2690	0.2695	0.2736	0.2803	0.2833	0.2708 ± 0.0106	
a	0.9352	0.9485	0.9482	0.9455	0.9438	0.9410	0.9436	0.9511	0.9610	0.9643	0.9482 ± 0.0088	
F_{norm}	0.1520	0.4129	0.5376	0.6276	0.6982	0.7549	0.8030	0.8462	0.8847	0.9182	-	L_1, z = 14.6 mm, NT
b	0.3366	0.3135	0.3169	0.3182	0.3186	0.3180	0.3173	0.3176	0.3183	0.3183	0.3193 ± 0.0062	
a	0.9270	0.9175	0.9191	0.9192	0.9190	0.9174	0.9162	0.9171	0.9182	0.9182	0.9189 ± 0.0030	

7 Propagation of error

7.2 Addabs IFE correction

The standard deviation of the i-th background-corrected measurement $s(\mathrm{BC})_i$ can be calculated simply by combining the contributions of the standard deviation of the measured sample, s_i, and the standard deviation of the background (absorber without fluorophore), $s(\mathrm{B})_i$, as shown in eq S15 and eq S16.

$$s(\mathrm{BC})_i{}^2 = s_i{}^2 + s(\mathrm{B})_i{}^2 \quad \text{(S15)}$$

$$s(\mathrm{BC})_i = \sqrt{s_i{}^2 + s(\mathrm{B})_i{}^2} \quad \text{(S16)}$$

7.3 Lakowicz IFE correction

IFE correction method proposed by Lakowicz (F_A) is described via the eq S17.

$$F_\mathrm{A} = F_0 10^{[(A_\mathrm{ex}+A_\mathrm{em})/2]} \quad \text{(S17)}$$

This correction is a function of 3 variables: F_0 (measured, uncorrected fluorescence), A_ex (absorbance at excitation wavelength), A_em (absorbance at emission wavelength). Each of the 3 variables were measured and their standard deviation is calculated from the replicated measurements as shown in eq S18.

$$s = \sqrt{\frac{1}{n-1}\sum_{i=1}^{n}(x_i - \bar{x})^2} \quad \text{(S18)}$$

Partial derivatives of said function with respect to all 3 variables are shown in eqs S19-S21.

$$\frac{\partial F_\mathrm{A}(F_0, A_\mathrm{ex}, A_\mathrm{em})}{\partial F_0} = 10^{[(A_\mathrm{ex}+A_\mathrm{em})/2]} \quad \text{(S19)}$$

$$\frac{\partial F_{\mathrm{A}}(F_0,A_{\mathrm{ex}},A_{\mathrm{em}})}{\partial A_{\mathrm{ex}}} = F_0 \ln(10)\, 2^{\left[\frac{1}{2}(A_{\mathrm{ex}}+A_{\mathrm{em}}-2)\right]} 5^{\left[\frac{1}{2}(A_{\mathrm{ex}}+A_{\mathrm{em}})\right]} \tag{S20}$$

$$\frac{\partial F_{\mathrm{A}}(F_0,A_{\mathrm{ex}},A_{\mathrm{em}})}{\partial A_{\mathrm{em}}} = \frac{\partial F_{\mathrm{A}}(F_0,A_{\mathrm{ex}},A_{\mathrm{em}})}{\partial A_{\mathrm{ex}}} \tag{S21}$$

Error estimate (including the correlation) is calculated as shown in eq S22.

$${s_{F_{\mathrm{A}}}}^2 = \left(\frac{\partial F_{\mathrm{A}}}{\partial F_0} s_{F_0}\right)^2 + \left(\frac{\partial F_{\mathrm{A}}}{\partial A_{\mathrm{ex}}} s_{A_{\mathrm{ex}}}\right)^2 + \left(\frac{\partial F_{\mathrm{A}}}{\partial A_{\mathrm{em}}} s_{A_{\mathrm{em}}}\right)^2$$

$$+2\left(\frac{\partial F_{\mathrm{A}}}{\partial F_0}\frac{\partial F_{\mathrm{A}}}{\partial A_{\mathrm{ex}}}\right)\mathrm{cov}(F_0, A_{\mathrm{ex}}) + 2\left(\frac{\partial F_{\mathrm{A}}}{\partial F_0}\frac{\partial F_{\mathrm{A}}}{\partial A_{\mathrm{em}}}\right)\mathrm{cov}(F_0, A_{\mathrm{em}}) + 2\left(\frac{\partial F_{\mathrm{A}}}{\partial A_{\mathrm{ex}}}\frac{\partial F_{\mathrm{A}}}{\partial A_{\mathrm{em}}}\right)\mathrm{cov}(A_{\mathrm{ex}}, A_{\mathrm{em}}) \tag{S22}$$

7.4 ZINFE/NINFE correction

The ZINFE and NINFE corrections (F_{Z} and F_{N}, respectively) are shown via eq S23. The difference between these two corrections is that the exponent N is determined directly from the experimental values in the case of the ZINFE method, whereas N is optimized numerically in the case of the NINFE method. The error in the exponent is mainly due to the error in the height of the fluid in the microplate well. F_1 and F_2 are measured fluorescence values at 2 different z-positions.

The partial derivatives of the ZINFE/NINFE correction function with respect to the 3 variables F_1, F_2, N are shown in eqs. S24-S26.

$$F_{\mathrm{Z/N}} = F_{0(z1)}\left(\frac{F_{0(z1)}}{F_{0(z2)}}\right)^N \tag{S23}$$

$$\frac{\partial F_{\mathrm{Z/N}}(F_{0(z1)},F_{0(z2)},N)}{\partial F_{0(z1)}} = (N+1)\left(\frac{F_{0(z1)}}{F_{0(z2)}}\right)^N \tag{S24}$$

$$\frac{\partial F_{\mathrm{Z/N}}(F_{0(z1)},F_{0(z2)},N)}{\partial F_{0(z2)}} = -N\left(\frac{F_{0(z1)}}{F_{0(z2)}}\right)^{(1+N)} \tag{S25}$$

$$\frac{\partial F_{\mathrm{Z/N}}(F_{0(z1)},F_{0(z2)},N)}{\partial N} = F_{0(z1)}\left(\frac{F_{0(z1)}}{F_{0(z2)}}\right)^{N} \ln\left(\frac{F_{0(z1)}}{F_{0(z2)}}\right) \tag{S26}$$

Variance (including the correlation) is calculated as shown in the eq S27.

$$s_{F_{\mathrm{Z/N}}}{}^{2} = \left(\frac{\partial F_{\mathrm{Z/N}}}{\partial F_{0(z1)}} s_{F_{0(z1)}}\right)^{2} + \left(\frac{\partial F_{\mathrm{Z/N}}}{\partial F_{0(z2)}} s_{F_{0(z2)}}\right)^{2} + \left(\frac{\partial F_{\mathrm{Z/N}}}{\partial N} s_{N}\right)^{2}$$

$$-2\mathrm{cov}\left(F_{0(z1)}, F_{0(z2)}\right)(N+1)N\left(\frac{F_{0(z1)}}{F_{0(z2)}}\right)^{N}\left(\frac{F_{0(z1)}}{F_{0(z2)}}\right)^{(1+N)} \tag{S27}$$

8 Preliminary tests of the AddAbs method using the 90-degree angle fluorimeter setup

8.1 Testing method

Preliminary tests of the AddAbs method were performed using the fluorometric cuvette in a typical 90-degree angle fluorimeter setup. To prove the broad applicability of the method, two different concentration series of the QS fluorophore were measured with and without the presence of the PD absorber (as for measurements in microplates). The values for $A_{345nm,QS,1cm}$ were 1.841 and 24.35 for the most concentrated ($c_{norm} = 1$) solutions of the low and high concentration series, respectively (the values for $A_{360nm,QS,1cm}$ are 1.466 and 19.39, respectively). The low concentration series contained x100 diluted saturated PD absorber solution, while the high concentration series contained x60 diluted absorber stock solution. The solutions were manually pipetted into plastic tubes to achieve a total volume of 2 mL. A fused silica ultra-micro fluorescence cell (model QS105.250, Hellma, Germany) with an optical path length of 10 × 2 mm was used for the measurements.

For the 90-degree angle fluorimeter configuration, the fluorescence must occur in the central region of the cuvette to be measured. The corresponding effective optical paths for the cuvette used in this work can be approximated as 10 mm / 2 = 5 mm and 2 mm / 2 = 1 mm. For the lower QS concentration series, the measurements were performed according to the standard procedure, using the longer effective optical path of approximately 5 mm for excitation. In contrast, for the higher QS concentration series, measurements were performed by rotating the cuvette 90 degrees, resulting in a shorter effective optical path of approximately 1 mm for the incident light. This approach was chosen because of the extreme pIFE observed at high concentrations of QS and PD, which results in insufficient light reaching the center of the cuvette to produce a reliable signal. Therefore, the effective optical path can be reduced to increase the range of acceptable optical sample densities.

The cuvette with a chamber volume of 100 µL was washed three times with 300 µL of the sample before each measurement. Measurements were performed using the RSM 1000F (Olis, USA) rapid-scanning fluorimeter with a LED actinic source with an excitation maximum of $\lambda_{max} \approx 360$ nm. Each emission spectrum represents an average of 1250 fluorescence spectra measured in 20 s. The voltage on the photomultiplier was optimized and adjusted separately for each titration series to avoid signal clipping. In parallel, aliquots of 200 µL for each sample were manually pipetted into the UV-vis-transparent (T) plate and fluorescence was measured at both 345 and 360 nm excitation to account for possible differences caused by different excitation wavelengths.

8.2 AddAbs IFE-correction results

The AddAbs IFE-correction method produced satisfactory results for both the high and low QS concentration series ($R^2 > 0.98$), as shown in Figures S43 and S44. In these figures, the fluorescence values obtained in the cuvette at the emission maxima (at $c_{norm} = 1$) are shown. Moreover, virtually

identical results were obtained for the low and high concentration series, regardless of the excitation wavelength at 345 or 360 nm (Figures S45 and S46). A closer look at Figures S43-S46 shows that the main difference between the measurements with the different instruments is the nonlinearity of the IFE-uncorrected fluorescence. One of the fundamental differences is that in the case of the standard fluorimeter, the excitation light must penetrate deep into the solution (i.e., to the center of the cuvette) to produce a detectable signal. In the case of the microplate reader, the fluorescence contribution from the optically densest excitation region is still likely to be significant. For the same reason, the reduction in sensitivity due to the addition of chromophores is comparatively less remarkable for measurements in microplates. In the experiments presented here, the IFE-induced variability (nonlinearity) of fluorescence is much more pronounced when fluorescence is measured in microplates.

In our estimation, the front-face configuration is more likely to give better results than the 90-degree configuration because the excitation light does not have to penetrate very deeply into the cuvette to produce a detectable signal. The front-face fluorescence measurements have similarities to measurements made with a microplate reader, as seen in Figures S40-S42, which show different setups for fluorescence measurements. The 90-degree configuration may require high-bandwidth measurements and/or switching to other excitation wavelengths to obtain meaningful signals. In these cases, the AddAbs method could still be used to reduce the residual spectral variability caused by different sample concentrations. The AddAbs method may be particularly suitable for situations where polychromatic light sources are used, and no monochromator is available. In such cases, light filtering can be conveniently performed directly in the solutions by using the added absorber as a liquid optical filter.

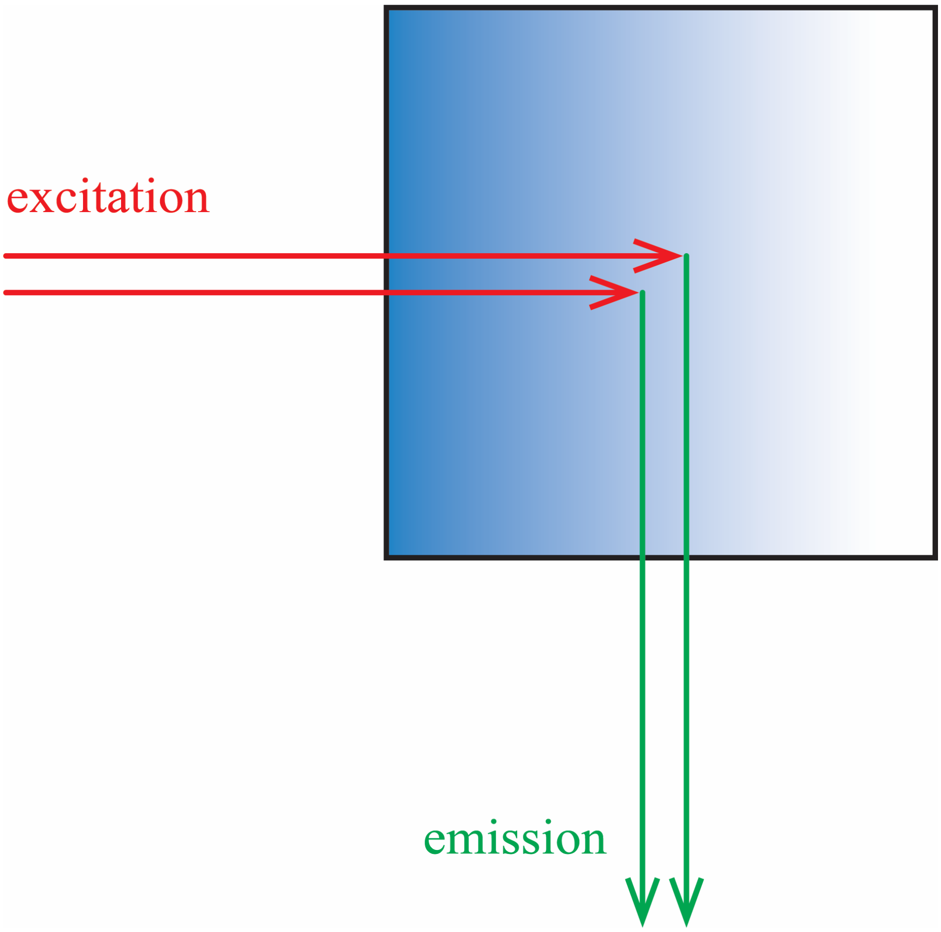

Figure S40. 90-degree configuration for fluorescence measurements performed in rectangular cuvettes.

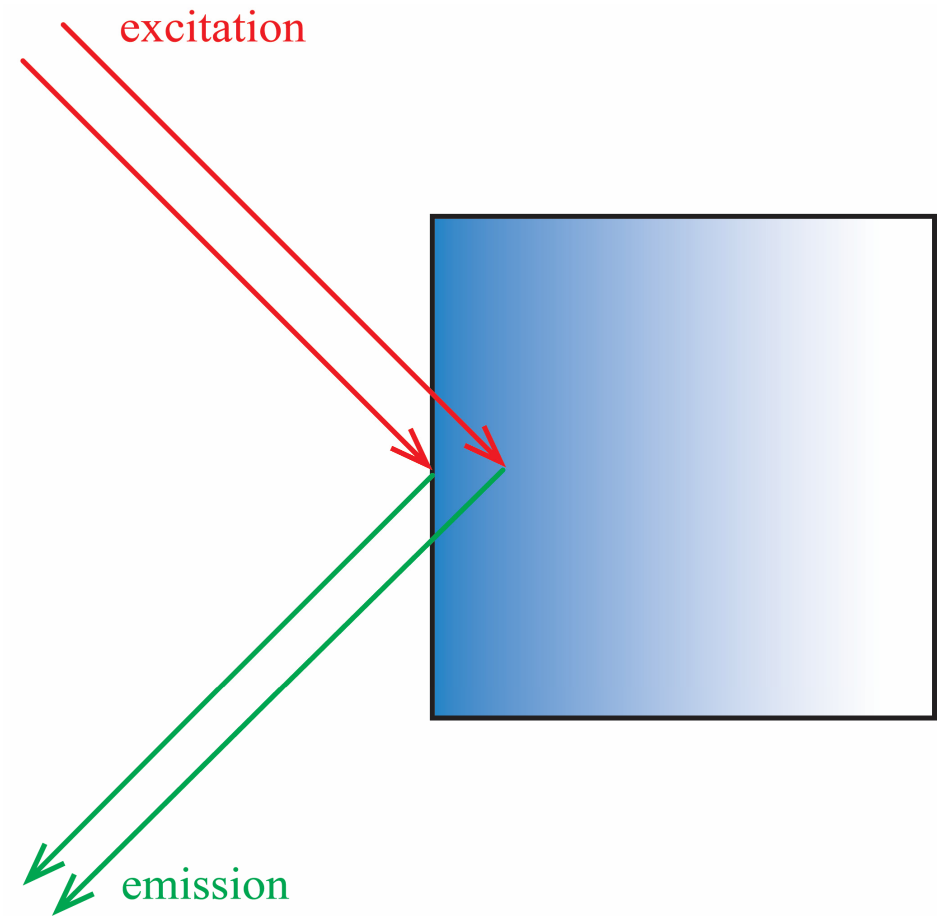

Figure S41. Front-face configuration for fluorescence measurements performed in rectangular cuvettes.

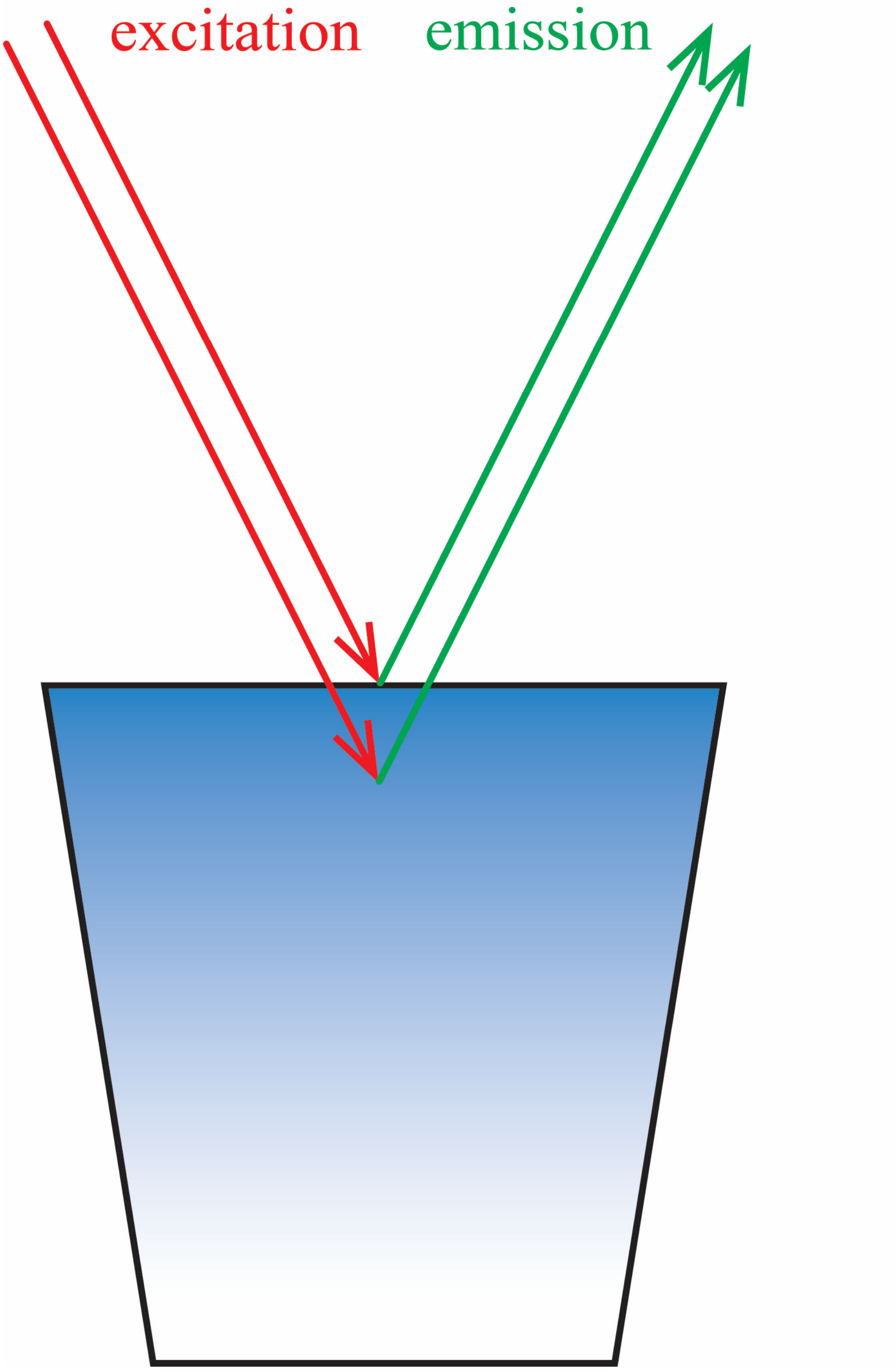

Figure S42. Top-reading configuration for fluorescence measurements performed in microplate wells.

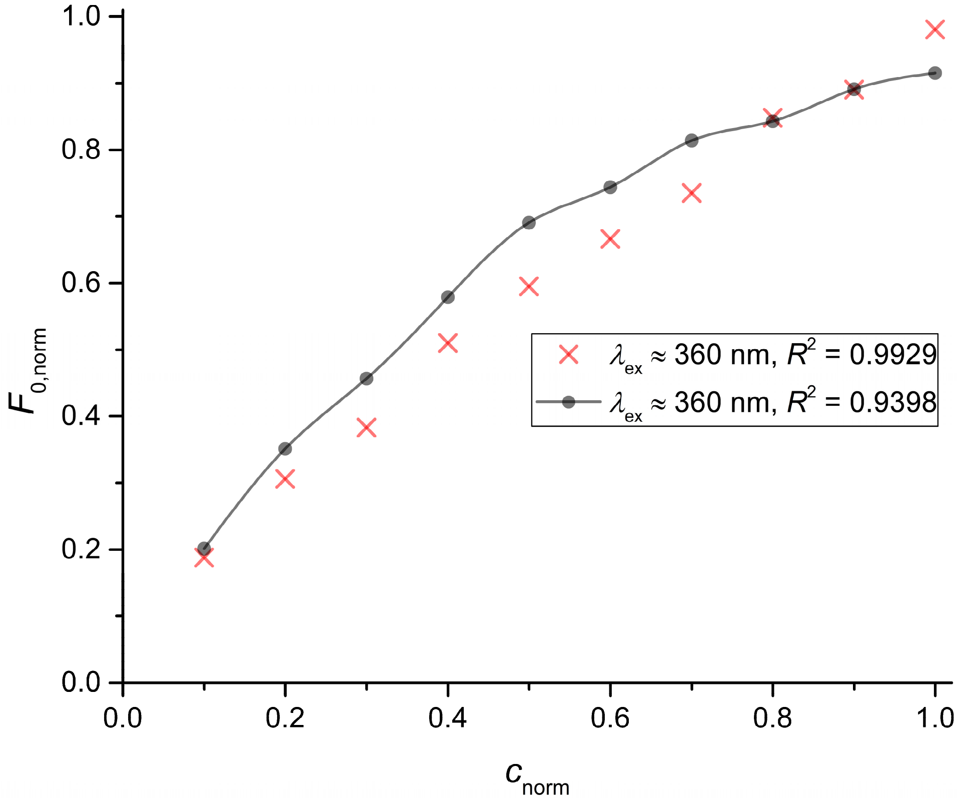

Figure S43. AddAbs correction results (red) obtained for the low QS concentration series using a standard fluorimeter with 90-degree angle configuration. IFE-uncorrected results (black) are shown for comparison, $\lambda_{ex} \approx 360$ nm, QS absorbance $A_{360nm,max,1cm} = 1.466$, $l_{ex,cuvette\ center} \approx 5$ mm.

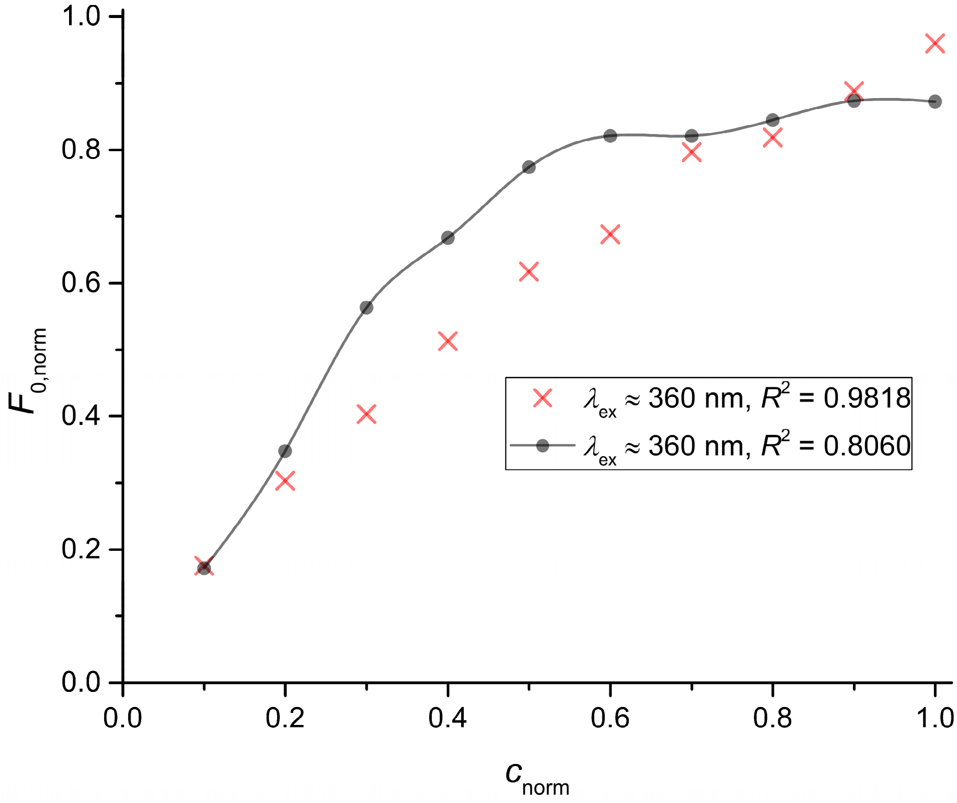

Figure S44. AddAbs correction results (red) obtained for the high QS concentration series using a standard fluorimeter with 90-degree angle configuration. IFE-uncorrected results (black) are shown for comparison, $\lambda_{ex} \approx 360$ nm, QS absorbance $A_{360nm,max,1cm} = 19.39$, $l_{ex,cuvette\ center} \approx 1$ mm.

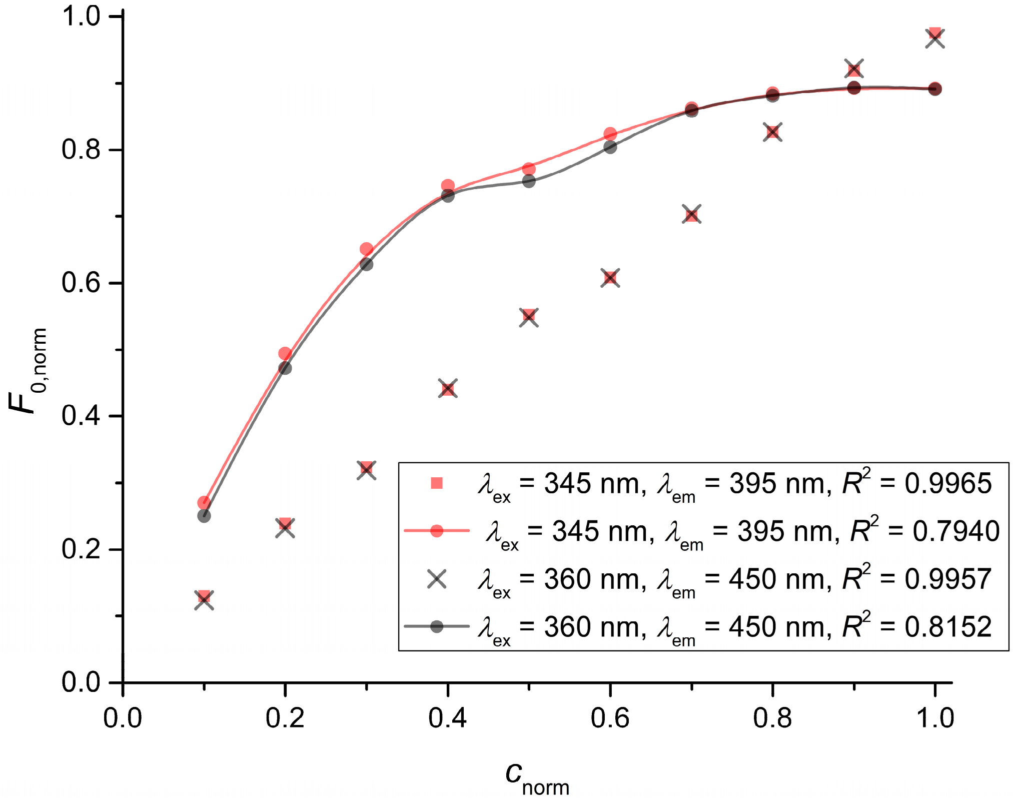

Figure S45. AddAbs correction results (red squares, black crosses) obtained for the low QS concentration series using a microplate reader. IFE-uncorrected results (black and red dots) are shown for comparison, QS absorbance: $A_{345\text{nm,max,1cm}}$ = 1.841, $A_{360\text{nm,max,1cm}}$ = 1.466, z = 17 mm.

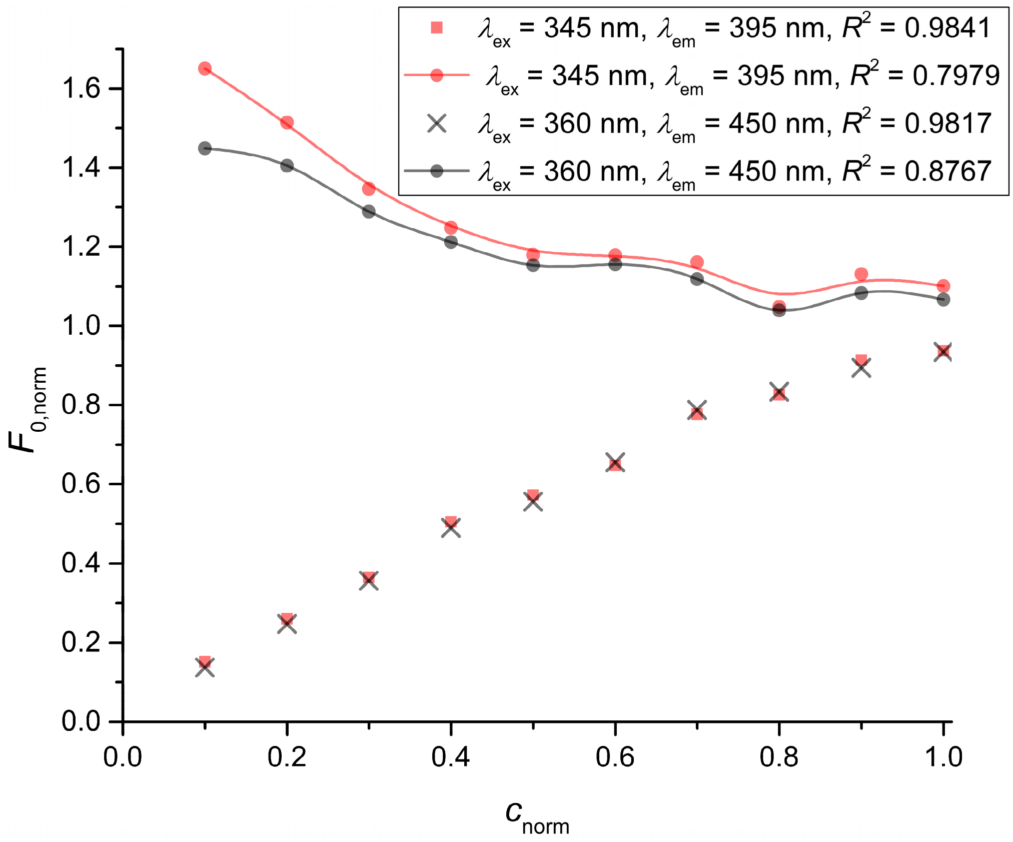

Figure S46. AddAbs correction results (red squares, black crosses) obtained for the high QS concentration series using a microplate reader. IFE-uncorrected results (black and red dots) are shown for comparison, QS absorbance: $A_{345nm,max,1cm}$ = 24.35, $A_{360nm,max,1cm}$ = 19.39, z = 17 mm.

9 References

(1) Miller, J. N.; Miller, J. C. *Statistics and Chemometrics for Analytical Chemistry*, 6. ed.; Prentice Hall: Harlow, 2010.

(2) Taylor, J. R. *An Introduction to Error Analysis: The Study of Uncertainties in Physical Measurements*, 2nd ed.; University Science Books: Sausalito, Calif, 1997.

(3) Brunetti B, D. E. About Estimating the Limit of Detection by the Signal to Noise Approach. *Pharm Anal Acta* **2015**, *06* (04). https://doi.org/10.4172/2153-2435.1000355.

(4) Weitner, T.; Friganović, T.; Šakić, D. Inner Filter Effect Correction for Fluorescence Measurements in Microplates Using Variable Vertical Axis Focus. *Anal. Chem.* **2022**, *94* (19), 7107–7114. https://doi.org/10.1021/acs.analchem.2c01031.

(5) Larsson, T.; Wedborg, M.; Turner, D. Correction of Inner-Filter Effect in Fluorescence Excitation-Emission Matrix Spectrometry Using Raman Scatter. *Analytica Chimica Acta* **2007**, *583* (2), 357–363. https://doi.org/10.1016/j.aca.2006.09.067.

(6) Tucker, S. A.; Amszi, V. L.; Acree, W. E. Primary and Secondary Inner Filtering. Effect of $K_2Cr_2O_7$ on Fluorescence Emission Intensities of Quinine Sulfate. *J. Chem. Educ.* **1992**, *69* (1), A8. https://doi.org/10.1021/ed069pA8.

10 Conflicts of interest

There are no conflicts of interest to declare.

11 Acknowledgements

This work was supported by funding from the Croatian Science Foundation grant UIP-2017-05-9537 – Glycosylation as a factor in the iron transport mechanism of human serum transferrin (GlyMech). Additional support was provided by the European Regional Development Fund grants for ‘Croatian National Centre of Research Excellence in Personalized Healthcare’ (contract #KK.01.1.1.01.0010), ‘Centre of Competences in Molecular Diagnostics’ (contract #KK.01.2.2.03.0006), ‘Strengthening of Scientific Research and Innovation Capacities of the Faculty of Pharmacy and Biochemistry at the University of Zagreb’ (contract #KK.01.1.1.02.0021), and ‘Development of methods for production and labelling of glycan standards for molecular diagnostics’ (contract #KK.01.1.1.07.0055).
